# Folding and duplex formation in mixed sequence recognition-encoded *m*-phenylene ethynylene polymers†

**DOI:** 10.1039/d1sc02288a

**Published:** 2021-07-06

**Authors:** Giulia Iadevaia, Jonathan A. Swain, Diego Núñez-Villanueva, Andrew D. Bond, Christopher A. Hunter

**Affiliations:** Yusuf Hamied Department of Chemistry, University of Cambridge Lensfield Road Cambridge CB2 1EW UK herchelsmith.orgchem@ch.cam.ac.uk

## Abstract

Oligomers equipped with complementary recognition units have the potential to encode and express chemical information in the same way as nucleic acids. The supramolecular assembly properties of *m*-phenylene ethynylene polymers equipped with H-bond donor (**D** = phenol) and H-bond acceptor (**A** = phosphine oxide) side chains have been investigated in chloroform solution. Polymerisation of a bifunctional monomer in the presence of a monofunctional chain stopper was used for the one pot synthesis of families of *m*-phenylene ethynylene polymers with sequences **ADnA** or **DAnD** (*n* = 1–5), which were separated by chromatography. All of the oligomers self-associate due to intermolecular H-bonding interactions, but intramolecular folding of the monomeric single strands can be studied in dilute solution. NMR and fluorescence spectroscopy show that the 3-mers **ADA** and **DAD** do not fold, but there are intramolecular H-bonding interactions for all of the longer sequences. Nevertheless, 1 : 1 mixtures of sequence complementary oligomers all form stable duplexes. Duplex stability was quantified using DMSO denaturation experiments, which show that the association constant for duplex formation increases by an order of magnitude for every base-pairing interaction added to the chain, from 10^3^ M^−1^ for **ADA·DAD** to 10^5^ M^−1^ for **ADDDA·DAAAD**. Intramolecular folding is the major pathway that competes with duplex formation between recognition-encoded oligomers and limits the fidelity of sequence-selective assembly. The experimental approach described here provides a practical strategy for rapid evaluation of suitability for the development of programmable synthetic polymers.

## Abstract 2

One pot oligomerisation reactions give access to families of oligomers that allow facile analysis of folding propensity and assessment of suitability for sequence-selective duplex formation.

## Graphical abstract

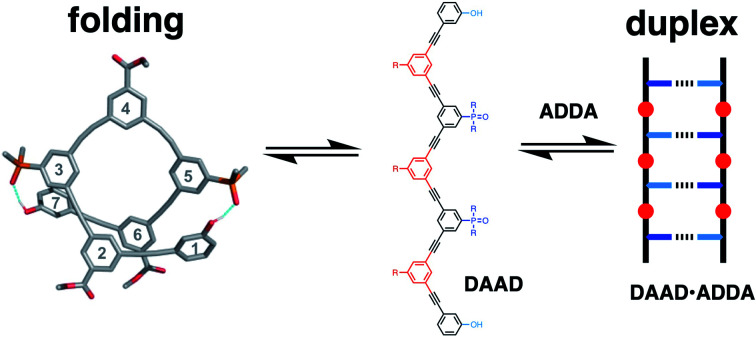

## Introduction

Linear oligomers of different monomeric building blocks are the key functional molecules of biological systems.^1–6^ Properties are encoded by the sequence of monomers in the polymer chain, and in principle, it should be possible to encode function in synthetic copolymers in the same way. Progress has been made in the development of synthetic oligomers that fold into well-defined three-dimensional structures based on the sequence of building blocks,^7–12^ or that form duplexes in a sequence-selective manner.^13–20^ Some of these synthetic systems also exhibit properties that resemble those found in biopolymers, such as substrate binding, self-assembly and catalysis.^21–27^ One of the most important functional properties found in biomolecules is the ability of nucleic acid oligomers to template the synthesis of a specific sequence of another oligomer, either protein or nucleic acid. This process is the molecular basis of evolution and is directly related to the duplex structure formed by nucleic acids.^28–30^ We have therefore been interested in the development of synthetic oligomeric systems that form duplexes in a sequence-selective manner, in the hope of recapitulating some of the functional properties of nucleic acids in synthetic polymers.

**Fig. 1:**
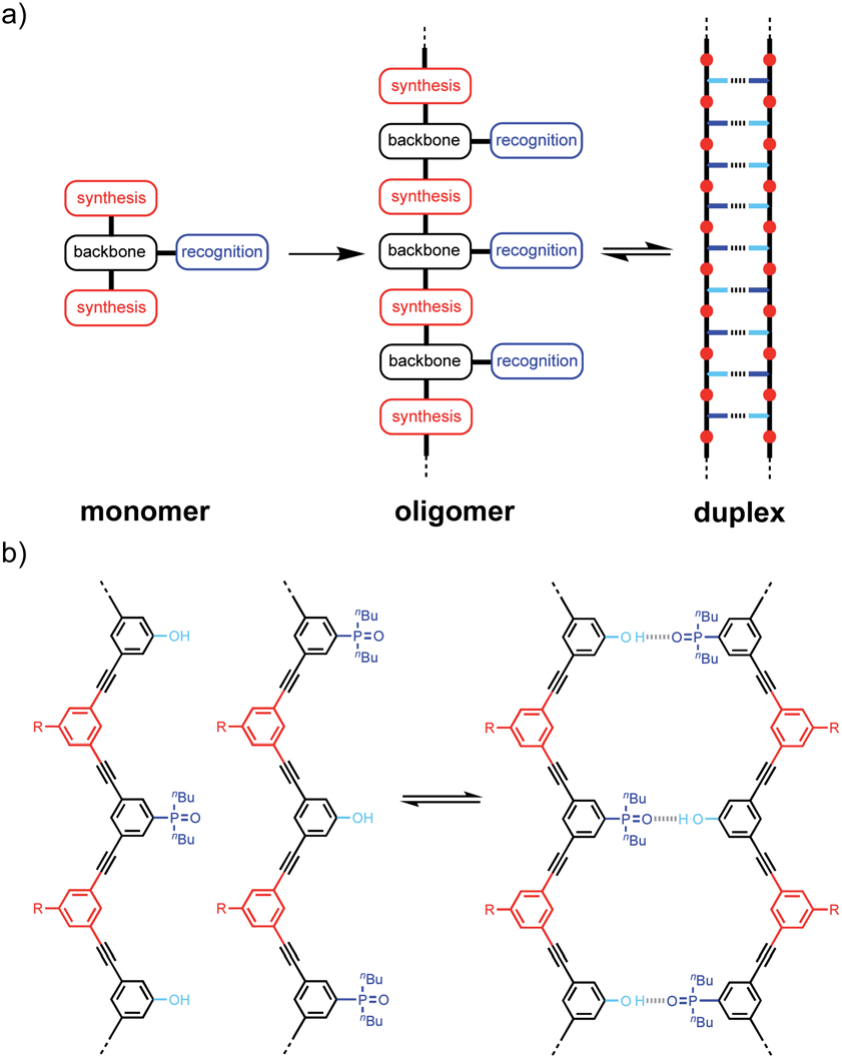
(a) A blueprint for duplex forming molecules, which require efficient coupling chemistry for the synthesis of oligomers (red), recognition modules for intermolecular binding (blue), and a backbone to link these components (black). (b) Proposed duplex formed by phenylacetylene oligomers equipped with phenol and phosphine oxide recognition modules (R is a solubilizing group).

The approach is illustrated in Fig. 1a. In principle, any polymerizable monomers that are equipped with complementary recognition units could be used to form oligomers capable of duplex formation. Fig. 1b shows one of the synthetic architectures that we have investigated.^31^ The monomers are dialkynyl benzenes that can be polymerised with di-iodobenzene linkers using Sonogashira coupling. The recognition units that are used to form the base-pairs that lead to duplex assembly are phenol and phosphine oxide side chains.

We have shown that if two complementary homo-oligomers are used, a wide range of different embodiments of the blueprint in Fig. 1a all lead to duplex formation.^32–37^ However for mixed sequence oligomers, the behaviour is less predictable. Intramolecular interactions between two complementary recognition units on the same oligomer lead to folding of single strands, and these folding equilibria compete with duplex formation.^38^ For many of the systems we have studied, H-bonding interactions between neighbouring recognition units (1,2-folding)^38^ or next neighbours (1,3-folding)^34^ leads to stable folded structures. 1,2-Folding prevents duplex formation in mixed sequence oligomers, and 1,3-folding erodes the sequence selectivity of duplex formation. Folding is an important property of single-stranded nucleic acids and determines the function of many RNA molecules in biology, but short oligomers do not fold, and 1,5-hairpin loops are the smallest folded structures,^39^ so that sequence-selective duplex formation is not significantly compromised.

The key parameter that determines the folding propensity of an oligomer is backbone conformation, and even for the relatively simple structures shown in Fig. 1b, we do not have reliable tools for *ab initio* prediction of conformational equilibria. Here we describe a synthetic strategy for rapidly accessing mixed sequence oligomers in order to characterise the competing equilibria of intramolecular folding and duplex formation. The approach presented here is based on a one-pot synthesis of a family of multiple mixed sequence oligomers. The sequences of these oligomers allow direct characterisation of all possible intramolecular folding equilibria from 1,2-folding up to 1,5-folding, thus providing an efficient and straightforward method for assessing the potential of a prospective backbone architecture for exploitation in sequence-selective duplex formation.

## Results and discussion

### Oligomer synthesis

**Fig. 2:**
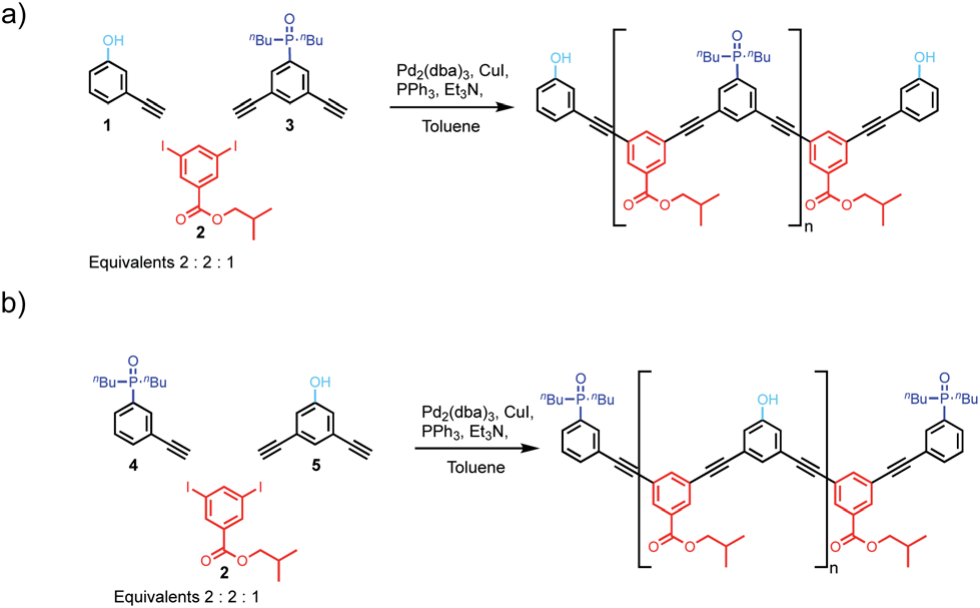
(a) Oligomerisation of the mono-alkyne phenol module and di-alkyne phosphine oxide module to yield **DAnD** oligomers. (b) Oligomerisation of the mono-alkyne phenol module and di-alkyne phosphine oxide module to yield **ADnA** oligomers.

We have previously reported the one-step synthesis of homo-sequence oligomers *via* statistically controlled oligomerisation followed by chromatographic separation to isolate pure oligomers of different lengths.^31^ To produce mixed sequence oligomers, the same approach can be used, except that mono-alkyne chain stoppers carrying one of the recognition units were mixed with di-alkynes carrying the other recognition unit, so that oligomerisation reactions with the di-iodo linker gave either **DAnD** or **ADnA** oligomers (Fig. 2).

Acceptor oligomers bearing donor capping groups were synthesised by oligomerisation of recognition modules **1** and **3** with the solubilising module **2** under Sonogashira conditions (Fig. 2).^40^ The ratio of mono-alkyne chain stopper to di-alkyne chain extender was chosen to reflect the intended composition of the desired product, in this case the 3-mer, *i.e.* an oligomer with three recognition units. The product mixture was dissolved in ethanol and analysed by LCMS. Oligomers up to the 8-mer were observed. The LCMS method was transferred to preparative HPLC, and the oligomers were separated (ESI Fig. S1†). Samples of **DAnD** oligomers from the 3-mer (**6**) to the 6-mer (**9**) were isolated (Table 1), and the most abundant oligomer was the 3-mer, as expected. The 7-mer was characterised by mass spectrometry, but the amount isolated was insufficient for characterisation by any other technique. The overall yield of oligomers with respect to the solubilising module was 29%.

**Table 1:** Isolated oligomers

Product	Sequence	Mass (mg)	% by mol fraction
6 (3-mer)	**DAD**	17.2	50
7 (4-mer)	**DAAD**	11.9	22
8 (5-mer)	**DAAAD**	16.7	23
9 (6-mer)	**DAAAAD**	4.7	5
10 (3-mer)	**ADA**	24.2	50
11 (4-mer)	**ADDA**	18.1	28
12 (5-mer)	**ADDDA**	11.5	15
13 (6-mer)	**ADDDDA**	4.4	5
14 (7-mer)	**ADDDDDA**	2.8	2

Similarly, donor oligomers bearing acceptor capping groups were synthesised by oligomerisation of recognition modules **4** and **5** with the solubilising module **2** under Sonogashira conditions (Fig. 2). Oligomers up to the 8-mer were observed by LCMS, and they were separated by preparative HPLC. Samples of **ADnA** oligomers from the 3-mer (**10**) to 7-mer (**14**) were isolated (Table 1). The overall yield with respect to the solubilising module was 34%, and the most abundant oligomer was the 3-mer, matching the initial stoichiometry of the starting materials used in the reaction.

### Oligomer characterisation

**Fig. 3:**
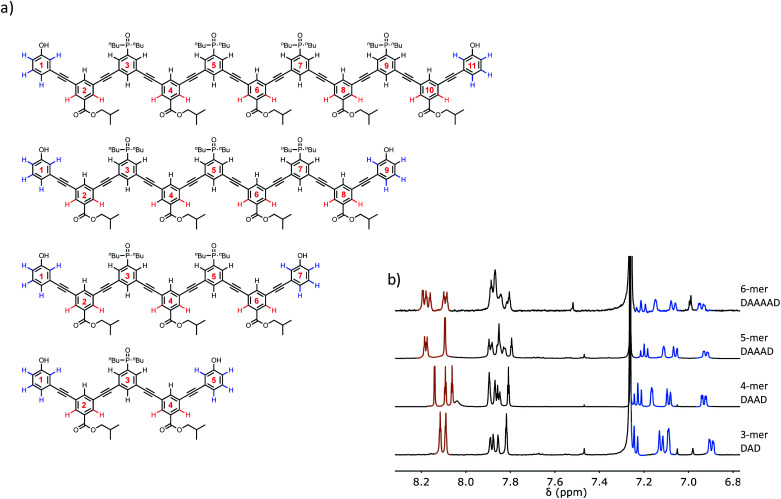
(a) Chemical structures of the **DAnD** oligomers. The residue numbering scheme is also shown. (b) Partial 500 MHz ^1^H NMR spectra recorded in CDCl_3_ at 298 K. The signals in the ^1^H NMR spectra are assigned to the chemical structures using colour coding.

**Fig. 4:**
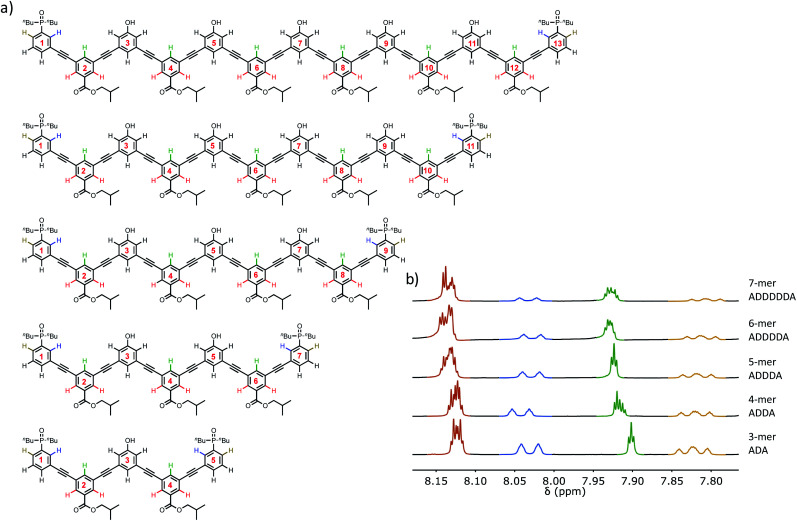
(a) Chemical structures of the **ADnA** oligomers. The residue numbering scheme is also shown. (b) Partial 500 MHz ^1^H NMR spectra recorded in THF-*d*_8_ at 298 K. The signals in the ^1^H spectra are assigned to the chemical structures using colour coding.

The structures of the oligomers were confirmed by mass spectrometry and ^1^H NMR spectroscopy. Fig. 3 and 4 show that distinct ^1^H NMR signals could be assigned to protons due to the terminal recognition modules, the internal recognition modules, and the solubilising modules. The ratios of the integrals of the signals due to the terminal recognition modules and solubilising modules were used to confirm oligomer length.

### Self-association of 3-mers

**Fig. 5:**
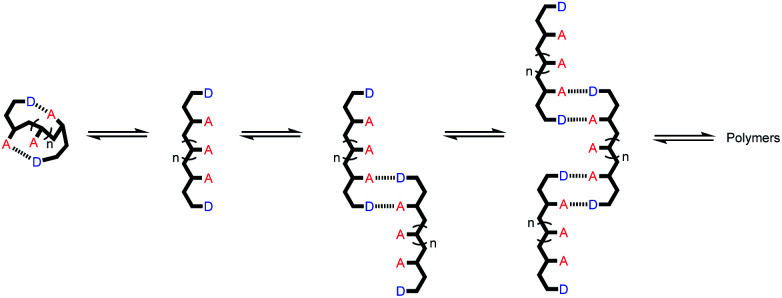
Possible equilibria for folding and self-association of **DAnD** oligomers.

The **DAnD** and **ADnA** oligomers could fold *via* intramolecular interactions or self-associate *via* the doubly H-bonded **AD·AD** duplex motif shown in Fig. 5 to give supramolecular polymers. We first studied the 3-mers ADA and DAD, which can dimerise *via* the **AD·AD** motif, but cannot polymerise. The association constants for dimerization of DAD to give **DAD·DAD** and dimerization of ADA to give **ADA·ADA** were measured by ^31^P and ^1^H NMR dilution experiments in CHCl_3_, and the results are summarised in Table 2.

**Table 2:** Association constants (*K*_a_) and limiting chemical shifts (ppm) measured in CDCl_3_ at 298 K by ^31^P NMR titration and dilution experiments

	*K*_a_ (M^−1^)	*δ*_free_	*δ*_bound_	Δ*δ*
**A·D** a	63 ± 23	40.3	44.3	4.1
**AA·DD** b	240 ± 80	40.2	44.4	4.2
**AD·AD** b	130 ± 30	40.2	44.1	3.9
**DAD·DAD**	490 ± 1	40.0	44.3	4.3
**ADA·ADA**	1360 ± 70	40.2	42.2	2.0

a The previously reported value (30 M^−1^) was obtained by fitting the titration data to a 1 : 1 binding isotherm.^38^ However, fitting to an isotherm that allows for a weak second binding interaction is significantly better and gives the value reported here.

b Values reported previously.^31,38^

**Fig. 6:**
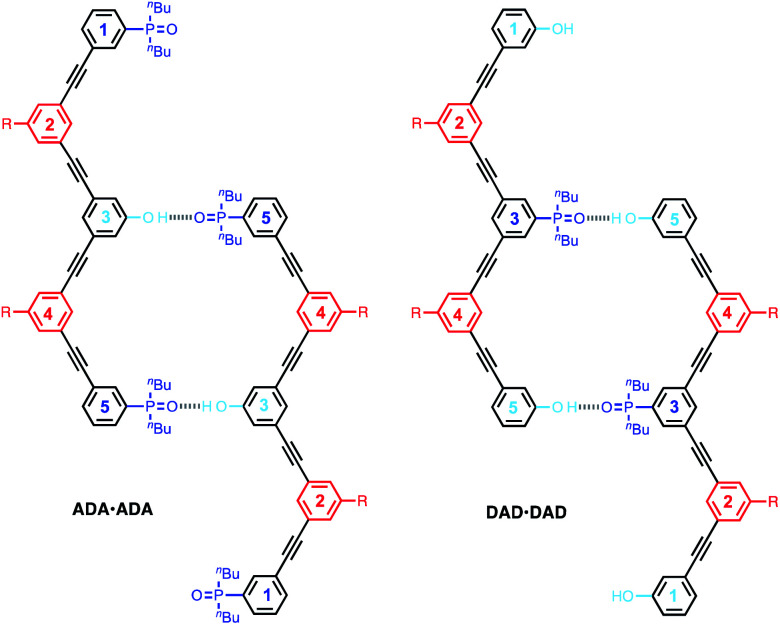
Self-association of **ADA** and **DAD**.

The limiting free ^31^P NMR chemical shifts of the signals due to the phosphine oxide groups are about 40 ppm in both cases, which is the same as the value measured for free 1-mer **A**, 2-mer **AA** and 2-mer **AD** (Table 2). This result indicates that the 3-mers **ADA** and **DAD** do not form intramolecular H-bonds in the monomeric state, which is consistent with previous experiments on the **AD** 2-mer. In other words, the 1,2-folded state is not populated to any significant extent for these oligomers.^38^ The complexation-induced change in chemical shift for formation of the **DAD·DAD** complex is about 4 ppm, which is the same as the value measured for the fully H-bonded complexes **A·D**, **AA·DD** and **AD·AD** (Table 2). The complexation-induced change in chemical shift for formation of the **ADA·ADA** complex is half of this value (2 ppm), because in the doubly H-bonded duplex, there are two terminal phosphine oxides that do not make any H-bonds (Fig. 6). The observed complexation-induced change in chemical shift for formation of **ADA·ADA** is therefore the population weighted average due to one bound and one free phosphine oxide group.

The association constant for formation of the **AA·DD** duplex is about double the value for the **AD·AD** duplex, due to the difference in the degeneracies of the complexes. The association constant measured for formation of the **DAD·DAD** duplex is slightly higher than the value for the **AD·AD** duplex, and this is due to the presence of additional non-bonded terminal bases. Fig. 6 illustrates H-bonding interactions involving the phenol residues labelled 5 on both chains. However, complexes can also be formed by H-bonding with the phenol residues labelled 1, leading to four degenerate complexes, *i.e.* using phenol residues 5 + 5, 1 + 5, 5 + 1, or 1 + 1. The association constant measured for formation of the **ADA·ADA** duplex is similar to, but somewhat higher than the value measured for **DAD·DAD**.

**Fig. 7:**
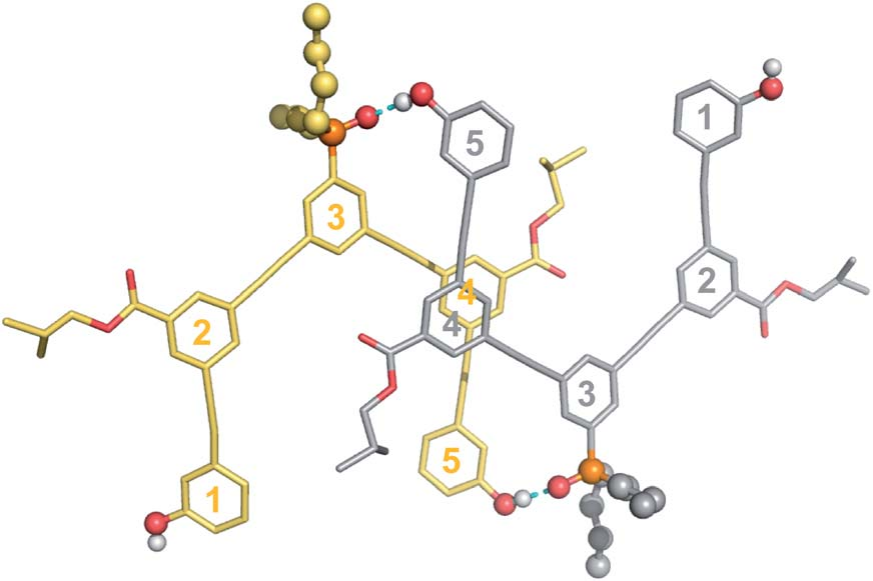
Single-crystal X-ray structure of **DAD**, which forms a doubly H-bonded duplex in the solid state. The recognition units are highlighted as balls, and the H-bonds are shown in blue. The residue numbering scheme from Fig. 3 is shown. Hydrogen atoms have been omitted for clarity.

Single crystals of **DAD** were obtained by slow evaporation from 10% CH_2_Cl_2_ in acetonitrile, and the X-ray crystal structure was determined. DAD forms a duplex in the solid state, and in agreement with the solution-phase NMR experiments, there is no intramolecular H-bonding in the crystal structure (Fig. 7). The solid state structure of the duplex shows that in addition to the two phenol-phosphine oxide H-bonds between residues 3 and 5, there is a stacking interaction between two of the solubilising modules, which are labelled residue 4 in both oligomers. Closer examination of the ^1^H NMR signals from the dilution experiments shows that the complexation-induced changes in chemical shift for the protons on residues 2 and 4 are very small (+0.1 ppm), which suggests that the stacking interaction observed in the crystal probably does not persist in chloroform solution.

### Self-association and folding of longer oligomers

Self-association of the other oligomers was also studied in CHCl_3_ by NMR dilution experiments. These systems all have two AD motifs and so can polymerise as illustrated in Fig. 5. The NMR dilution data for **DAAD** and **DAAAD** fit well to an isodesmic polymerisation isotherm, but for **DAAAAD**, there was insufficient material to obtain a complete isotherm. The self-association constants are reported in Table 3, and the values are similar to the values measured for the doubly H-bonded duplexes reported in Table 2. This result suggests that the longer **DAnD** oligomers all polymerise *via* the **AD·AD** duplex motif. However, the limiting free chemical shifts of the signals due to the phosphine oxides are significantly higher than the value for a free phosphine oxide, which is 40 ppm (see Table 2). This observation suggests that there is intramolecular H-bonding between the recognition modules in the monomeric state of these longer oligomers. Although a complete binding isotherm could not be obtained for **DAAAAD**, the ^31^P NMR spectrum of a 50 μM solution of this oligomer was recorded. Under these conditions, self-association should be minimal, so the observed chemical shifts are likely to be representative of the monomeric state. For this oligomer, the two signals due to the phosphine oxide groups appear at 41.0 and 41.4 ppm, suggesting that **DAAAAD** also folds to some extent due to intramolecular H-bonding.

**Table 3:** Association constants (*K*_a_) and limiting chemical shifts (ppm) measured in CDCl_3_ at 298 K by ^31^P NMR dilution experiments

	*K*_a_ (M^−1^)	Signal^d^	*δ*_free_	*δ*_bound_	Δ*δ*
**DAAD·DAAD** a	1200 ± 600	P3, P5	41.3	42.9	1.7
**DAAAD·DAAAD** a	420 ± 140	P5	40.9	42.4	0.5
		P3, P7	41.7	42.4	1.5
**DAAAAD·DAAAAD** b	—	P3, P9	41.4	—	—
		P5, P7	41.0	—	—
**ADDA·ADDA** c	22 000 ± 13 000	P1, P7	41.9	43.7	1.8
**ADDDA·ADDDA** c	14 000 ± 3000	P1, P9	43.0	44.8	1.8

a Fit to an isodesmic polymerisation isotherm.

b Insufficient material was isolated to obtain a complete isotherm.

c Fit to a dimerisation isotherm.

d The residue numbering scheme in Fig. 3 and 4 is used for the ^31^P signals.

The behaviour of the longer **ADnA** oligomers is quite different, with self-association constants that are more than an order of magnitude higher. This result indicates that rather than polymerising *via* the doubly H-bonded **AD·AD** duplex motif, these oligomers form dimers with more than two intermolecular H-bonds. The dilution data fit well to a dimerization isotherm, and the results are reported in Table 3 (**ADDDDA** was not sufficiently soluble to obtain a complete isotherm). The limiting free ^31^P NMR chemical shifts of the signals due to the phosphine oxide groups are significantly higher than 40 ppm, which suggests that these oligomers also fold due to intramolecular H-bonding interactions between recognition modules in the monomeric state.

**Fig. 8:**
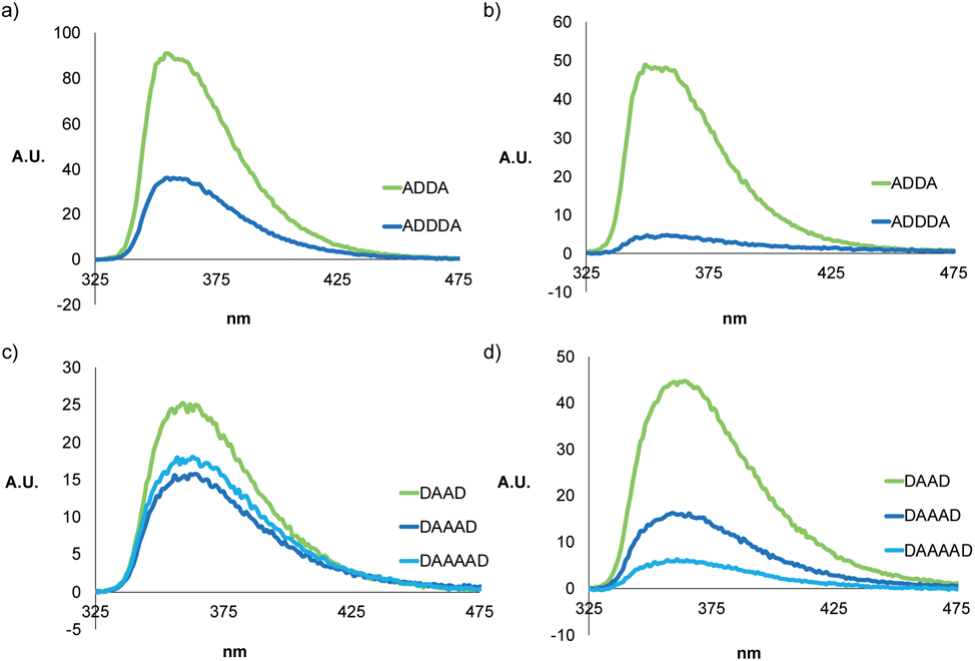
Fluorescence spectra of 10 μM solutions of **ADnA** in (a) CHCl_3_ and (b) MeCN/CHCl_3_ (88 : 12 v/v), and **DAnD** in (c) CHCl_3_ and (d) MeCN/CHCl_3_ (88 : 12 v/v).

A different class of phenylacetylene oligomers have been studied by Moore *et al.*, and they report that oligomers with eight or more phenyl rings fold into helices.^41–43^ In acetonitrile, stacking of the aromatic rings drives folding and can be monitored by fluorescence quenching and changes in the ^1^H NMR chemical shifts of the signals due to the aromatic protons. In chloroform, the Moore oligomers exist as random coils, and the fluorescence emission intensity increases with the length of the oligomer. We therefore investigated the folding properties of the monomeric forms of the mixed sequence phenol-phosphine oxide oligomers described here by measuring the fluorescence spectra in dilute solution, where there is no self-association. Spectra were recorded in chloroform and in chloroform–acetonitrile mixtures, and the results are shown in Fig. 8. The fluorescence intensity is lower for longer oligomers, which is consistent with the folding observed in the NMR experiments.

**Fig. 9:**
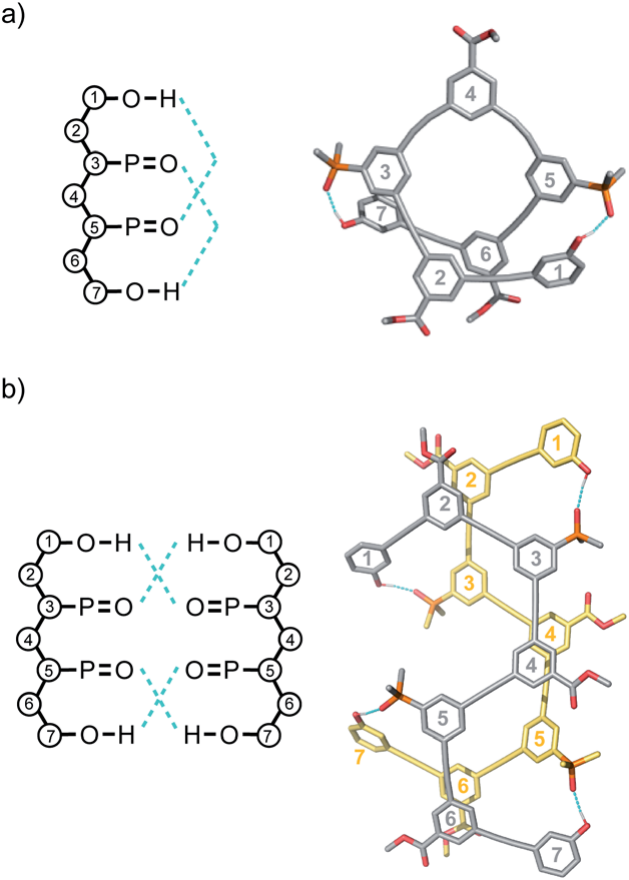
Lowest energy structures from molecular mechanics conformational searches (MMFFs with chloroform solvation) for (a) **DAAD** and (b) **DAAD·DAAD**. H-bonds are indicated in blue.

**Fig. 10:**
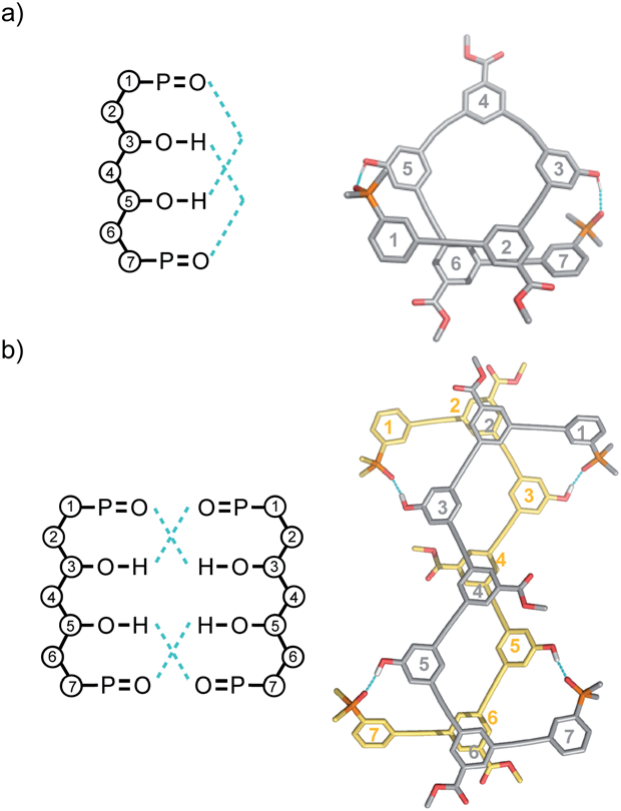
Lowest energy structures from molecular mechanics conformational searches (MMFFs with chloroform solvation) for (a) **ADDA** and (b) **ADDA·ADDA**. H-bonds are indicated in blue.

**Fig. 11:**
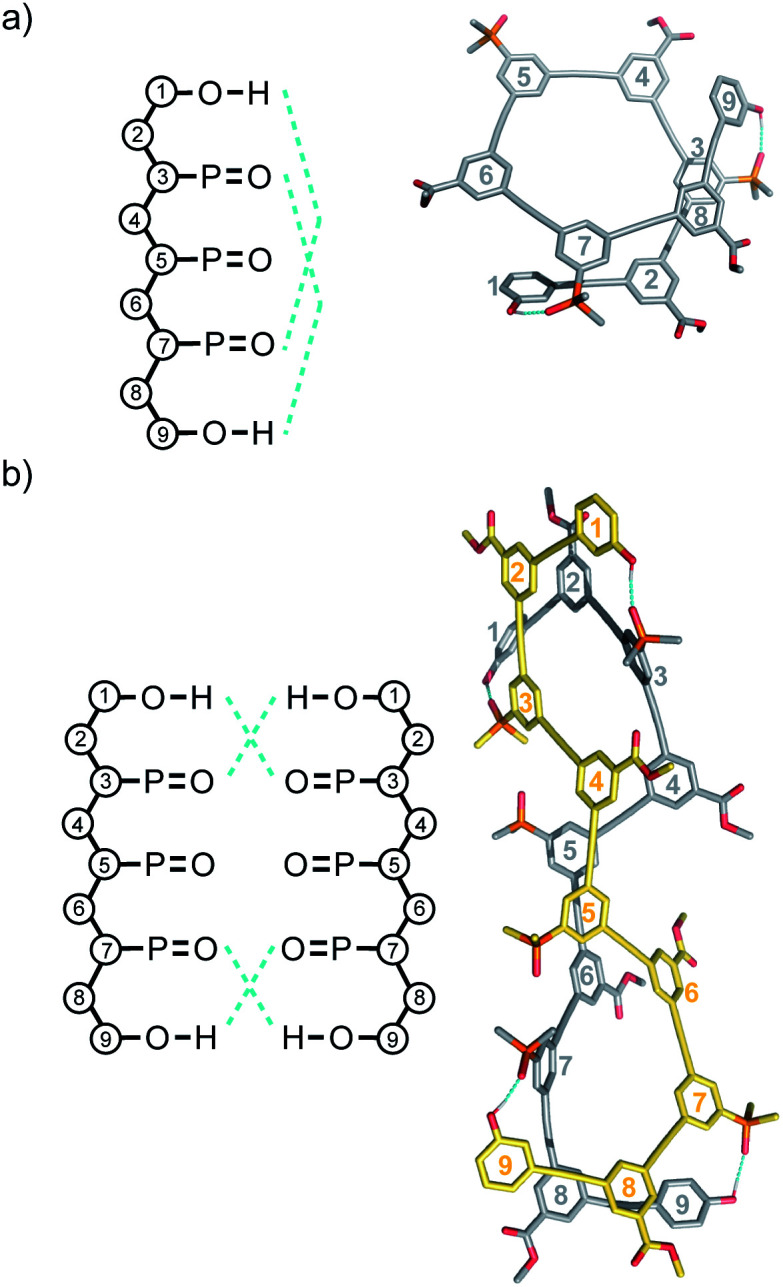
Lowest energy structures from molecular mechanics conformational searches (MMFFs with chloroform solvation) for (a) **DAAAD** and (b) **DAAAD·DAAAD**. H-bonds are indicated in blue.

**Fig. 12:**
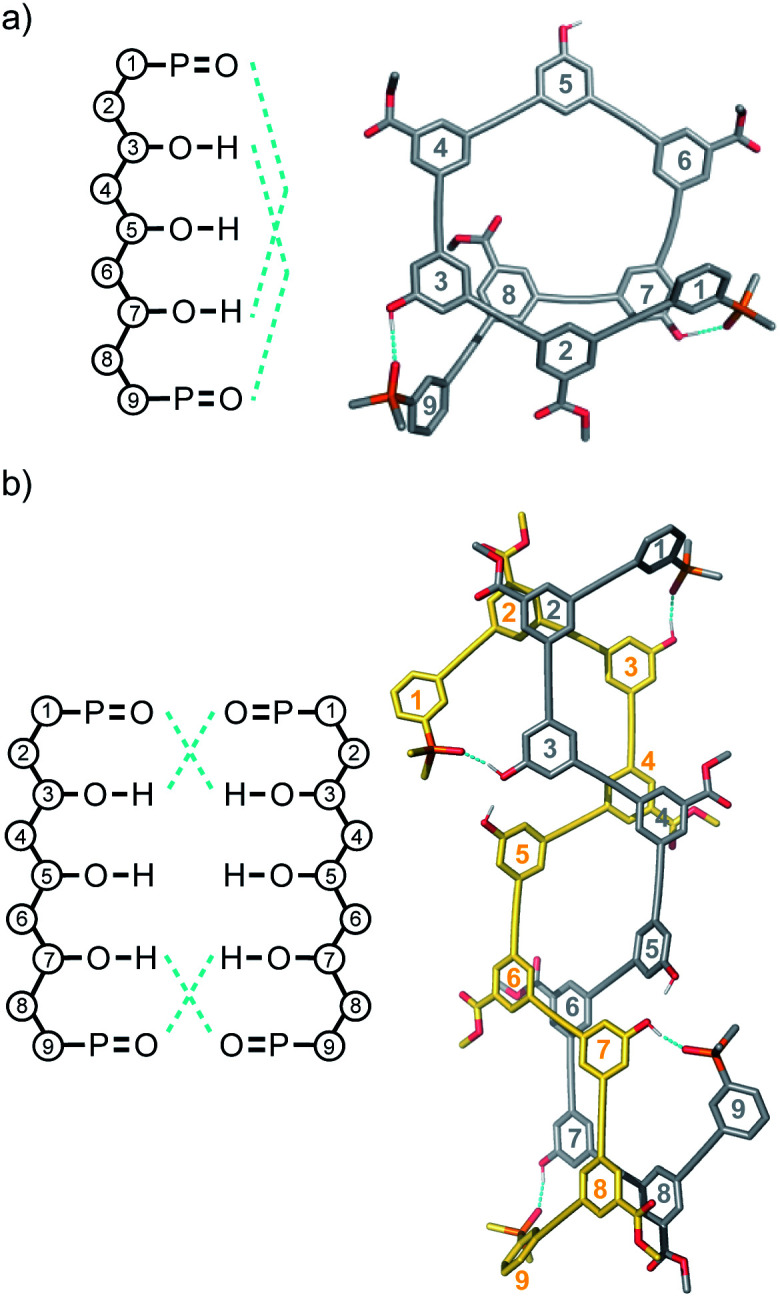
Lowest energy structures from molecular mechanics conformational searches (MMFFs with chloroform solvation) for (a) **ADDDA** and (b) **ADDDA·ADDDA**. H-bonds are indicated in blue.

Molecular mechanics calculations were used to investigate the three-dimensional structures of the oligomers in order to obtain information about the folding propensity of the single strands and the binding modes in the self-associated complexes. A conformational search using the MMFFs forcefield with chloroform solvation was performed for the single strands of the 4-mers and 5-mers. A helical folded conformation with intramolecular H-bonding was the lowest energy structure for all of these oligomers. Two intramolecular phenol-phosphine oxide H-bonds are formed in a helically folded structure for **DAAD** (Fig. 9a), and the same motif was found for **ADDA** (Fig. 10a). For **DAAAD**, the minimum energy structure has H-bonds between the phenols on rings 1 and 9 and the phosphine oxides on rings 3 and 7 (Fig. 11a). The phosphine oxide on ring 5 is not involved in H-bonding, which is consistent with the lower ^31^P NMR chemical shift observed for this group in the monomeric state (40.9 ppm for P5 compared with 41.7 ppm for P3/P7, see Table 3). The same helically folded motif was found for ADDDA (Fig. 12a). The molecular mechanics results suggest that folded structures are accessible for all the longer oligomers, in agreement with the NMR and fluorescence experiments. Molecular mechanics conformational searches were also carried out for the self-associated complexes, **DAAD·DAAD**, **ADDA·ADDA**, **DAAAD·DAAAD** and **ADDDA·ADDDA**. In all cases, the minimum energy structure was a duplex with 4 intermolecular H-bonds, which is made possible by the oligomers adopting a criss-cross conformation (Fig. 9b, 10b, 11b and 12b). The possibility of accessing duplex structures where more than two H-bonds are formed explains the very high association constants observed for the **ADnA** oligomers (Table 3). However, it is not clear from the modelling why the **DAnD** oligomers should prefer polymerisation over duplex formation.

### Duplex formation between sequence-complementary oligomers

**Fig. 13:**
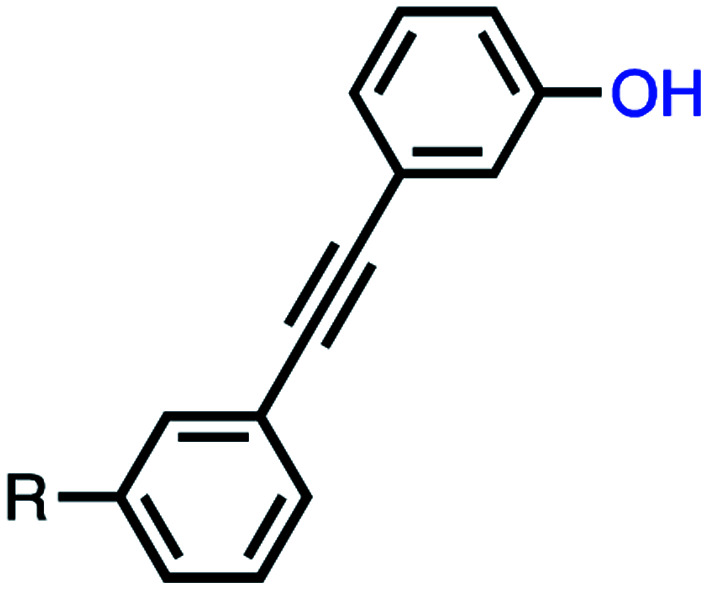
Phenol 1-mer (R = *S*-β-citronelloxy).

Measurement of the association constants for duplex formation by titration experiments is complicated by the multiple competing folding and self-association equilibria in these systems. However, we have previously shown that DMSO denaturation experiments can be used to determine association constants for duplex formation.^31^ Provided the duplex is the most stable species in a 1 : 1 mixture of two oligomers, the denaturation experiment avoids competition with intramolecular folding and self-association, because there will always be an excess of DMSO to prevent formation of these species when the duplex dissociates. Increasing amounts of DMSO were added to 1 : 1 mixtures of the sequence complementary oligomers, **AAA·DDD**, **ADA·DAD**, **DAAD·ADDA** and **DAAAD·ADDDA** in chloroform. In order to analyse the denaturation data, the association constant for the complex formed between DMSO and a phenol monomer is required. DMSO was titrated into a solution of the phenol 1-mer shown in Fig. 13 in chloroform, and the ^1^H-NMR data were fit to a 1 : 1 binding isotherm to determine a value of 27 ± 1 M^−1^ for the association constant. The high concentrations of DMSO used in the denaturation experiment can affect the ^31^P chemical shifts of the signals due to the unbound phosphine oxides. DMSO was therefore titrated into the **AAA** 3-mer,^31^ which cannot form any H-bonds, and the observed ^31^P NMR chemical shifts were used to correct the denaturation data for any non-specific effects due the change in solvent (see ESI†). We have shown previously the denaturation of the duplexes of the homo-oligomers is not an all-or-nothing process. The denaturation data were therefore fit to an isotherm that allows for population of partially denaturated species in addition to the duplex and the fully denatured state (see ESI†), and the results are reported in Table 4.

**Table 4:** Association constants (*K*_a_) and limiting chemical shifts (ppm) measured in CDCl_3_ at 298 K by ^31^P NMR denaturation experiments

	*K*_a_ (M^−1^)	Signal^a^	*δ* in 1.8 M DMSO	Δ*δ*
**AAA·DDD**	1900	P1, P5	40.2	2.9
		P3	40.0	2.9
**ADA·DAD**	2000	P1, P5	40.1	3.0
		P3	40.2	2.9
**ADDA·DAAD**	43 000	P1, P7	40.3	3.1
		P3, P5	40.1	2.7
**ADDDA·DAAAD**	120 000	P1, P9	40.3	3.3
		P3, P5, P7	40.0	2.3

a See Fig. 3 and 4 for ^31^P labelling scheme.

The duplex formed between the two homo-oligomers **AAA** and **DDD** was previously characterised in toluene and was included in this study in chloroform to provide a benchmark for the other duplexes. Neither intramolecular H-bonding nor oligomerisation is possible for the homo-oligomers, so this system provides a good test of the reliability of the denaturation experiment. The association constant determined for **AAA·DDD** is 2100 M^−1^, an order of magnitude higher than the value measured for **AA·DD** (240 M^−1^), indicating that a fully bound duplex with three intermolecular H-bonds is formed. The association constant for **ADA·DAD** is similar to the value measured for **AAA·DDD**, which indicates there are also three intermolecular phenol-phosphine oxide H-bonds in this duplex. There is an increase of another order of magnitude in the value of the association constant for the **ADDA·DAAD** duplex, which indicates the formation of four intermolecular H-bonds in this duplex. The increase in the association constant for the **ADDDA·DAAAD** duplex is a further order of magnitude, indicating formation of five intermolecular H-bonds in this complex. Table 4 also reports the ^31^P-NMR chemical shifts in 1.8 M DMSO measured at the end of the denaturation experiments. In all cases, the value is around 40 ppm, which corresponds to chemical shift of a free phosphine oxide (see Table 2), indicating that DMSO fully dissociates the complexes and prevents any intramolecular folding or self-association. The denaturation induced changes in ^31^P NMR chemical shift are similar (2–3 ppm) for all of the duplexes.

The association constants in Tables 2 and 4 can be used in eqn (1) to determine the effective molarity (EM) for the intramolecular H-bonding interactions that zip up the duplex.

1 *K*_N_ = 2*K*_1_(*K*_1_EM)^*N*−1^

where *N* is in the number of H-bonded base-pairs in the duplex, *K*_N_ is the association constant for duplex formation from two monomeric unfolded complementary oligomers, and the statistical factor of 2 reflects the two-fold degeneracy of these symmetrical duplexes.^21^

The average value of EM in chloroform is 60 mM, which is similar to the value previously measured in toluene solution using homo-oligomers (50 mM).

**Fig. 14:**
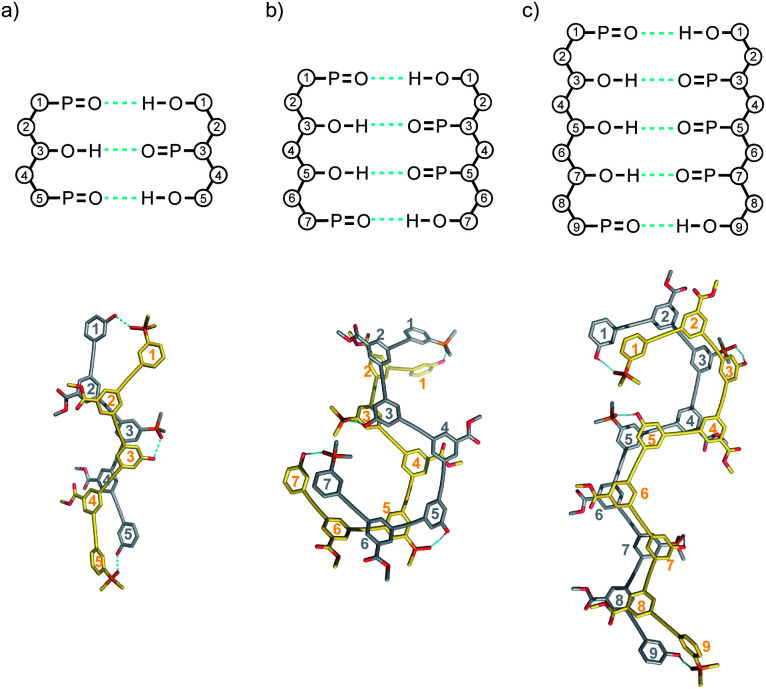
Lowest energy structures from molecular mechanics conformational searches (MMFFs with chloroform solvation) for (a) **ADA·DAD** and (b) **ADDA·DAAD** and (c) **ADDDA·DAAAD**. H-bonds are indicated in blue.

Molecular mechanics calculations were used to investigate the three-dimensional structures of the duplexes. A conformational search using the MMFFs forcefield with chloroform solvation was performed for **ADA·DAD**, **ADDA·DAAD** and **ADDDA·DAAAD**, and the results are illustrated in Fig. 14. In all cases, the lowest energy structure is the fully H-bonded duplex with all of the phenols and phosphine oxides paired in the correct order. The oligomer backbones adopt zig–zag rather than linear conformations, and in all of the structures, there is stacking of the phenyl rings carrying the solubilising groups along the length of the duplex.

## Conclusions

Polymers equipped with complementary recognition units have a rich supramolecular chemistry, where structure and function can be encoded in the sequence of building blocks. We have prepared a family of *m*-phenylene ethynylene oligomers which carry phenol and phosphine oxide recognition units as side chains. H-bonding interactions between these groups leads to folding, self-association of single strands, and duplex formation between complementary oligomers in non-polar solvents. By oligomerising a monomer equipped with one of the recognition units in the presence of a capping agent equipped with the other recognition unit, it was possible to prepare libraries of mixed sequence oligomers in a single reaction. These oligomers were separated by chromatography, and the properties of **DAnD** and **ADnA** sequences up to the 6-mer (*n* = 4) were investigated. NMR dilution experiments indicate that there is no 1,2-folding due to intramolecular H-bonding interactions between neighbouring recognition units in the 3-mers (**ADA** and **DAD**). In contrast, all of the longer oligomers show significant upfield shifts of the ^31^P NMR signals in the monomeric state in dilute solution, indicating that they fold to a significant extent due to 1,3 and 1,4 H-bonding interactions between the phenol and phosphine oxide recognition units. At higher concentrations, self-association of the **ADnA** oligomers leads to polymers that are held together by 2 intermolecular H-bonds, whereas the **DAnD** oligomers form dimeric structures with 4 intermolecular H-bonds. Despite these competing equilibria, 1 : 1 mixtures of pairs of sequence-complementary oligomers all form stable duplexes, and these systems were characterised by DMSO denaturation experiments in chloroform solution. The stability of the duplex increases by an order of magnitude for each recognition unit added to the oligomer up to an association constant of 10^5^ M^−1^ measured for **ADDDA·DAAAD**. These systems therefore appear to have potential for further investigation, and the prospect of using the sequence of one oligomer to template the synthesis of a complementary copy is one appealing possibility.

The approach to oligomer synthesis described here provides a rapid method for assessing new backbones for their potential for sequence-selective duplex formation and template synthesis. A major obstacle that we have encountered is intramolecular folding interactions between recognition units on the same strand, which compete with duplex formation. Oligomerisation of a monomer, which carries one type of recognition unit, in the presence of end capping groups, which carry the complementary recognition unit, gives access to a family of oligomers in one step and avoids multiple stepwise syntheses of different sequences. Assessment of the conformational properties of this family of oligomers under sufficiently dilute conditions to preclude intermolecular interactions reveals the presence of any intramolecular interactions between recognition units on the same strand, providing a direct read-out of the folding properties of the backbone. For example in the oligomers described here, the ^31^P NMR spectra of dilute solutions reveal whether the phosphine oxide recognition units are H-bonded to the phenol recognition units. In this case, there are no intramolecular interactions in the **ADA** or **DAD**, but H-bonds were detected in the longer oligomers, which shows that three or more bases are required to form stem-loop structures using this backbone. Suppression of such folded structures is likely to be key in the design of recognition-encoded oligomers that form duplexes with high sequence fidelity.

The building blocks used in the oligomerisation reactions described here afford a limited number of different sequences, which makes chromatographic separation viable. However, it is unlikely that separation of more complex mixtures would be practical, so solid phase methods similar to the approaches used in peptide and oligonucleotide synthesis will be required to access more diverse sequence space. The major challenge of working with longer mixed sequence oligomers is that the number of different sequences increases in a combinatorial manner with chain length, so careful experiment design will be required. An alternative is to develop evolutionary methods for searching sequence space based on replication, which would solve both the synthesis and sequence selection problems.^44^

## Data availability

All supporting data is provided in the ESI.†

## Author contributions

CAH devised the experiments, JAS, DNV and GI carried out the experiments and analysed the data, ADB solved the X-ray crystal structure, CAH, JAS, DNV and GI wrote the manuscript.

## Conflicts of interest

There are no conflicts to declare.

## Supplementary Material

SC-012-D1SC02288A-s001

SC-012-D1SC02288A-s002
