# Supplementary material for: Folding and duplex formation in mixed sequence recognition-encoded m-phenylene ethynylene polymers
Source: Chem Sci. 2021 Jul 6;12(30):10218–26. doi: 10.1039/d1sc02288a (PMC8336474; doi:10.1039/d1sc02288a)
Supplement: SC-012-D1SC02288A-s001 [file SC-012-D1SC02288A-s001.pdf]

Electronic Supplementary Material (ESI) for Chemical Science.
This journal is © The Royal Society of Chemistry 2021

Supporting information

Folding and duplex formation in mixed sequence recognition-encoded *m*-phenylene ethynylene polymers

Giulia Iadevaia, Jonathan A. Swain, Diego Núñez-Villanueva, Andrew D. Bond and Christopher A. Hunter*

Yusuf Hamied Department of Chemistry, University of Cambridge, Lensfield Road, Cambridge
CB2 1EW (UK) E-mail: herchelsmith.orgchem@ch.cam.ac.uk

Table of contents

Synthesis……………………………………………………………………………...2
HPLC Separation of Oligomers…………………………………………………3
Binding studies……………………………………………………………………………34
NMR dilution experiments……………………………………………………...34
NMR titration experiments……………………………………………………...37
NMR duplex denaturation experiments………………………………………...37
X-ray crystal structure of **DAD**……………………………………………………………47
Molecular Mechanics ……………………………………………………………..50

Synthesis

All the reagents were obtained from commercial sources (Sigma-Aldrich, Alfa Aesar, Fisher Scientific and Fluorochem) and were used without further purification. Thin layer chromatography was carried out using with silica gel 60F (Merck) on glass. ^{1}H and ^{13}C NMR spectra were recorded on either a Bruker 400 MHz AVIII400 or Bruker 500 MHz AVIII500 DCH cryoprobe spectrometer at 298 K unless specifically stated otherwise. Residual solvent was used as an internal standard. All chemical shifts are quoted in ppm on the δ scale and the coupling constants expressed in Hz. Signal splitting patterns are described as follows: s (singlet), d (doublet), t (triplet), sept (septet), m (multiplet). ES+ mass spectra were obtained on a Waters LCT premier mass spectrometer. FTIR spectra were recorded on a PerkinElmer Spectrum One FT-IR spectrometer. Synthesis of compounds **1, 2 3, 4** and **5** has been previously reported.[1]

1) J. A. Swain, G. Iadevaia and C. A. Hunter, *J. Am. Chem. Soc.*, 2018, **140**, 11526-11536

HPLC Separation of Oligomers

The samples were analyzed by reverse phase HPLC using an Agilent LC-MSD ionTrap model XCT LCMS equipment in Electrospray mode. This system is composed of a modular Agilent 1200 Series HPLC system connected to an Agilent/Bruker ionTrap model XCT with MSMS capabilities. The modular Agilent 1200 Series HPLC system is composed of a HPLC high pressure binary pump, autosampler with injector programming capabilities, column oven with 6 μL heat exchanger and a Diode Array Detector with a semimicro flow cell (6 mm path length, 1.7 μl volume) to reduce peak dispersion when using short columns as in this case. The flow-path was connected using 0.12 mm ID stainless steel tubing to minimize peak dispersion. The outlet of the Diode Array Detector flowcell is connected via a switching valve to the IonTrap, the switching valve allowing directing the first segment of the chromatography corresponding to solvent front to waste. After removing the contamination ions associated with the solvent front, the switching valve directs the solvent to the electronspray ion source. While the solvent rate of the method is 1mL/min, the ion source has a dead volume passive splitting union installed which splits the flow rate entering the ion source to <100 μL/min, the rest of the flow rate is directed to waste. This reduction in flow rate enhances the electrospray signal and reduces the contamination in the ion source. The Electrospray was set to +ve mode. The capillary needle has an orthogonal-flow sprayer design with respect to the ion transmission. The capillary needle voltage was set to +3500 V and the end plate offset was set to -500 V. The solvent eluting from the HPLC column entering the ESI capillary needle in the Ion Source Interface was nebulised with the assistance of N2 at 15 psi. Drying N2 gas heated to 325 °C and flowing at 5 L/min was used for the ESI desolvation stage. The ion transport and focusing region of the LC/MSD Trap is enclosed in the vacuum manifold, formed by a rough pump and two turbopumps. The ions formed on the Ion Source Interface enter and are guided through the glass capillary, where the capillary exit is set to -178 V. The bulk of the drying gas is removed by the rough pump before the skimmer which is set to -178 V. The ions then pass into an octopole ion guide (Octopole 1 set to -12 V DC followed by Octopole 2 set to -3 V DC set to a radio frequency of 200 Vpp) that focuses and transports the ions from a relatively high pressure position directly behind the skimmer to the focusing/exit lenses (Lens 1 set to +5 V followed by Lens 2 set to +60 V) coupling the ion transport to the ion trap. The selected ions entered the ion trap which had been set

to a value of 109.9. For efficient trapping and cooling of the ions generated by the electrospray interface, helium gas is introduced into the ion trap. The fractions were isolated using an Agilent HP-1100 preparative HPLC system. This is composed by a high pressure mixing binary pump capable of flow rates up to 50 mL/min at 400 bar back-pressure, with dual injector autosamplers lops (500 uL and a 5 mL loop), a variable detector (190 nm to 600 nm) and a fraction collector. UV/vis absorption was measured at 290 nm (8 nm bandwidth) with reference 550 nm (100 nm bandwidth). The software for the fraction collector can be set to automatically collect on peak recognition.

DAD oligomerization

1 (47.3 mg, 0.400 mmol), **3** (57.3 mg, 0.200 mmol) and **2** (172 mg, 0.400 mmol) were placed in a flask and degassed with N_2 for 30 minutes. $Pd_2(dba)_3$ (7.30 mg, 8.00 μmol) and CuI (1.50 mg, 8.00 μmol) and PPh_3 (10.5 mg, 40.0 μmol) were placed in a separate flask and degassed with N_2. Degassed Et_3N (167 μL, 1.20 mmol) was added and the contents of this flask transferred to the first using degassed toluene (8 mL). The reaction was stirred overnight at room temperature, in the dark under N_2. The solvent was removed by rotary evaporation under reduced pressure yielding a brown solid (465 mg). The sample was dissolved in EtOH and sonicated, before filtering. Preparative HPLC separation of the oligomerisation mixture was completed using a HIRBP-6988 prep column (250 x 25 mm, 5 μm particle size) with a water/THF solvent system (THF: 60% for 48 mins, 65% for 31 mins) at 15 mL min^{-1}.

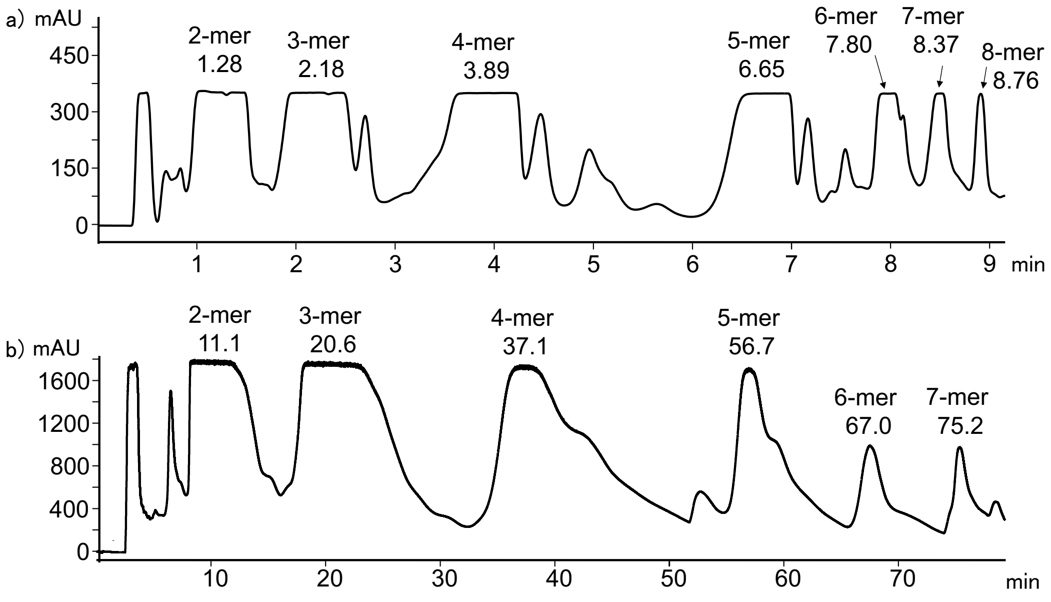

Figure S1 (a) LCMS analysis of DA_nD oligomerisation mixture using a Hichrom C_8C_{18} column (50 x 4.6 mm, 5 μm particle size) with a water/THF (0.1% formic acid) solvent system (THF: 60% for 5 mins, 60-70% over 2 mins 70% for 3 mins) at a flow rate of 1 mL min^{-1}. (b) Preparative HPLC (bottom) separation of DA_nD oligomerisation mixture using a HIRBP-6988 prep column (250 x 25 mm, 5 μm particle size) with a water/THF solvent system (THF: 60% for 48 mins, 65% for 31 mins) at 15 mL min^{-1}. Both samples were prepared in EtOH. UV/vis absorption was measured at 290 nm. Peaks identified by MS are labelled with retention time in minutes.

DAD 3-mer

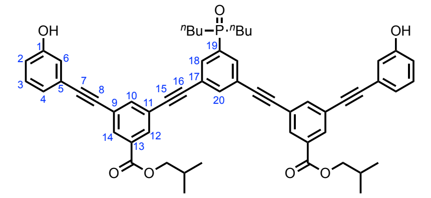

TLC R_f: 0.40 (MeOH:CH_2Cl_2 1:9);

^{1}H NMR (500 MHz, $CDCl_3$): δ 8.12 (t, J = 1.5 Hz, 2H, 12-H), 8.09 (t, J = 1.5 Hz, 2H, 14-H), 7.91 – 7.84 (m, 3H, 18, 20-H), 7.82 (t, J = 1.5 Hz, 2H, 10-H), 7.24 (t, J = 8.0 Hz, 2H, 3-H), 7.12 (dt, J = 7.5, 1.0 Hz, 2H, 4-H), 7.09 (dd, J = 2.5, 1.5 Hz, 2H, 6-H), 6.90 (ddd, J = 8.0, 2.5, 1.0 Hz, 2H, 2-H), 6.30 (bs, 2H, OH), 4.14 (d, J = 6.5 Hz, 4H, ^{i}Bu), 2.14 – 2.10 (m, 2H, ^{i}Bu), 2.07-1.81 (m, 4H, ^{n}Bu), 1.71-1.55 (m, 2H, ^{n}Bu), 1.49-1.35 (m, 6H, ^{n}Bu), 1.06 (d, J = 6.5 Hz, 12H, ^{i}Bu), 0.91 (t, J = 7.0 Hz, 6H, ^{n}Bu);

^{13}C NMR (126 MHz, $CDCl_3$): δ 165.2 (C=O), 156.0 (1-C), 138.4 (10-C), 137.6 (20-C), 133.7 (d, J = 88.0 Hz, 19-C), 133.0 (18-C) 132.6 (12-C), 132.0 (14-C), 131.3 (13-C), 129.7 (3-C), 124.2 (4-C), 124.1 (17-C), 123.7 (11-C), 123.2 (9-C), 118.3 (6-C), 116.5 (2-C), 90.9 (-C≡), 90.4 (-C≡), 89.9 (-C≡), 87.2 (-C≡), 71.6 (^{i}Bu), 29.2 (d, J = 68.5 Hz, ^{n}Bu), 27.9 (^{i}Bu), 24.1 (d, J = 14.5 Hz, ^{n}Bu), 23.5 (d, J = 4.0 Hz, ^{n}Bu) 19.2 (^{i}Bu), 13.6 (^{n}Bu);

^{31}P NMR (202 MHz, $CDCl_3$-d_3): δ 43.7 (phosphine oxide)

FT-IR (ATR): 3148 (br), 2923, 2224, 1722, 1591, 1232, 767 ν_{max}/cm^{-1};

HRMS (ES+): $C_{56}H_{56}O_7P$ calcd. 871.3758 found 871.3719, Δ = -4.50 ppm.

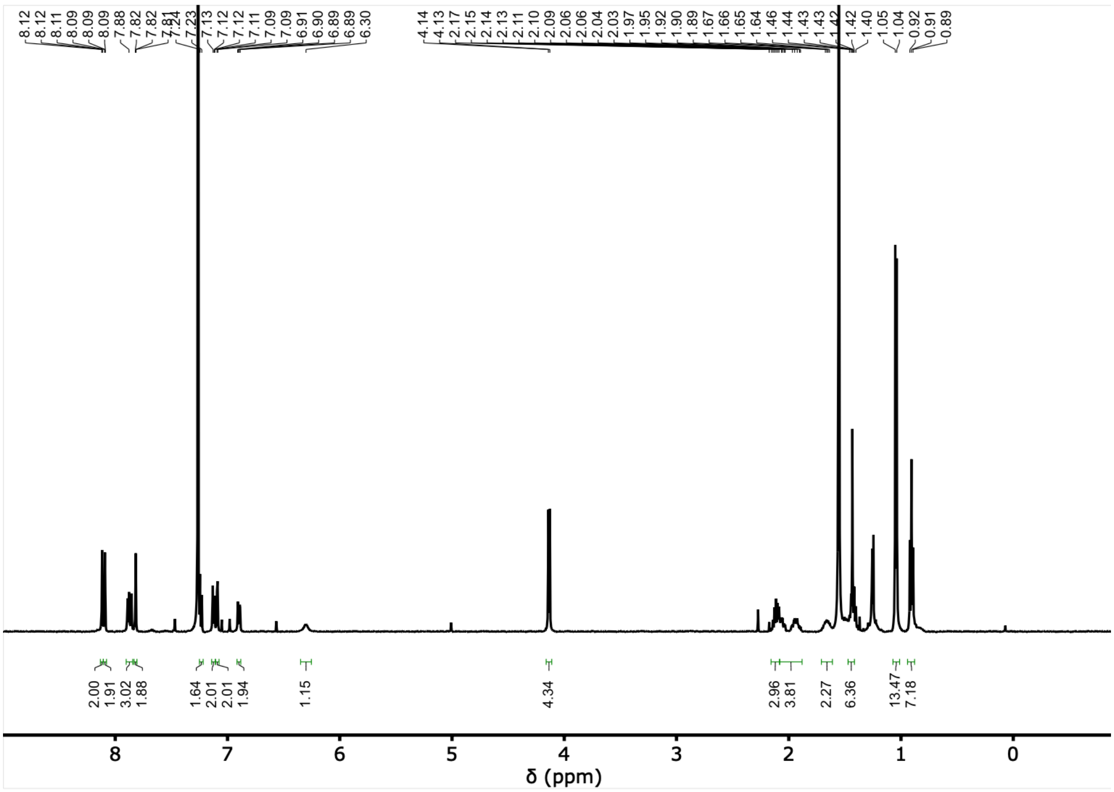

Figure S2 ^{1}H NMR (500 MHz, $CDCl_3$) of DAD

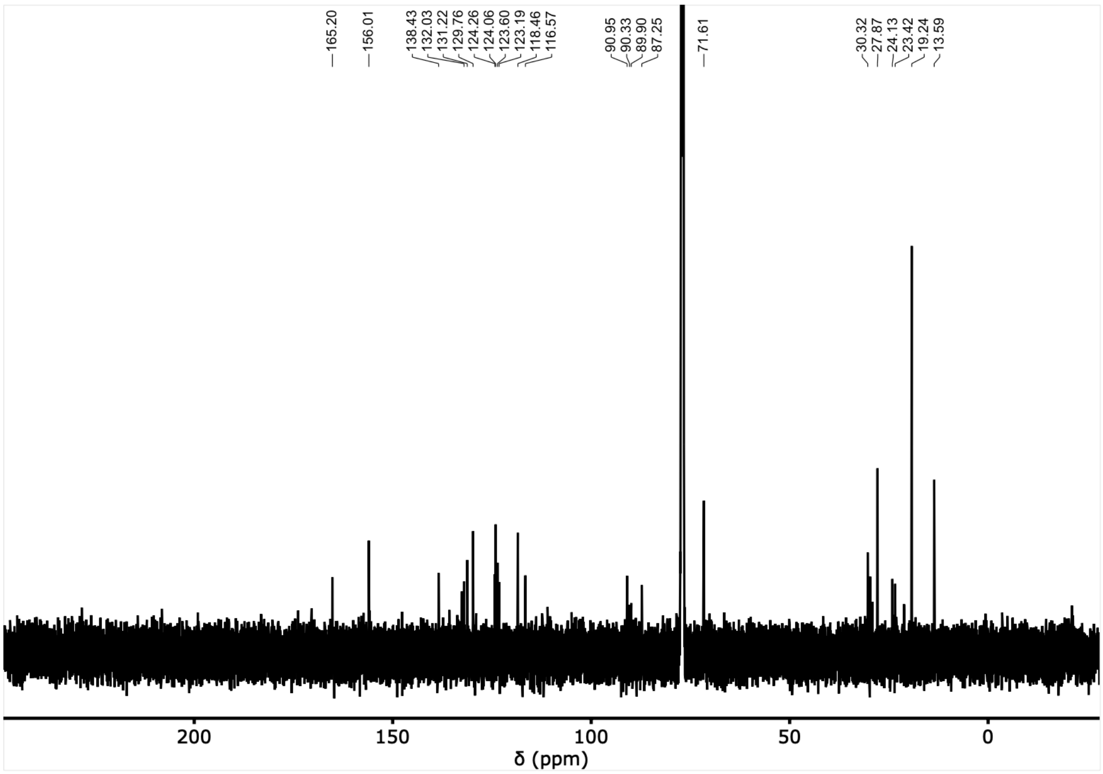

Figure S3 ^{13}C NMR (126 MHz, $CDCl_3$) of DAD

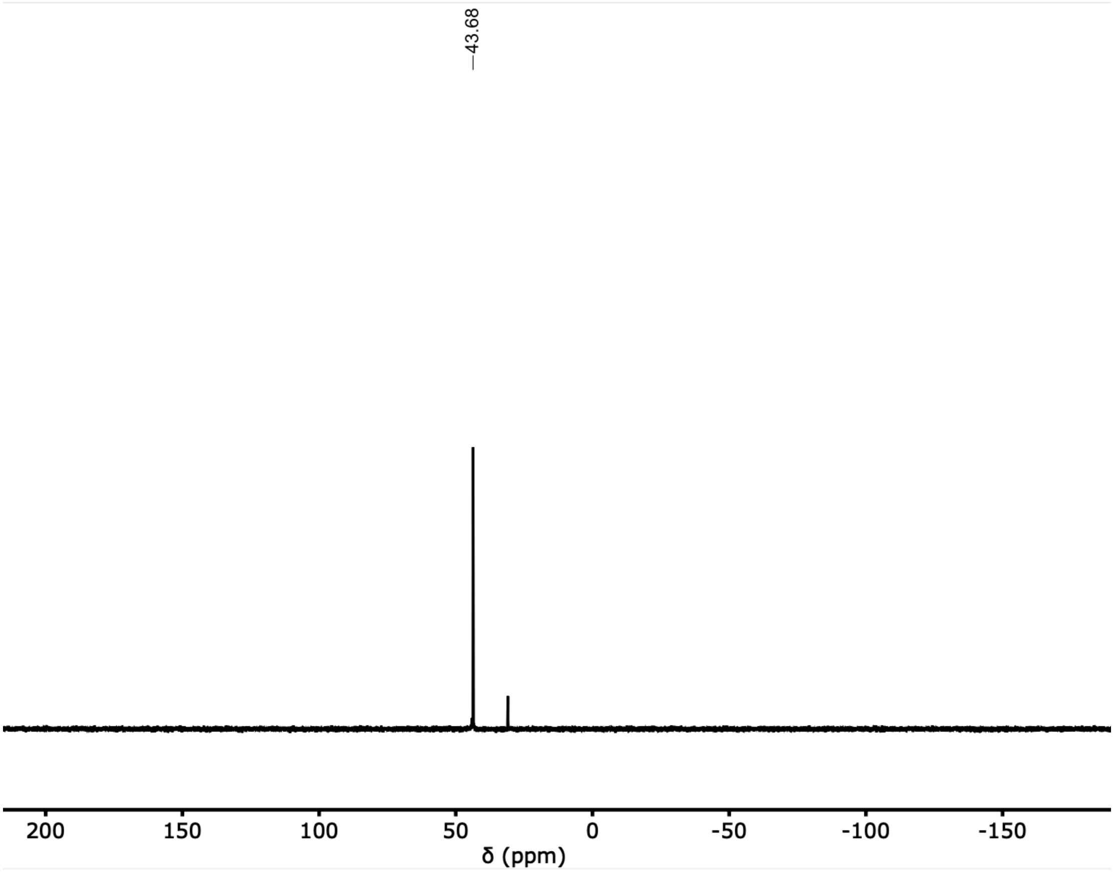

Figure S4 ^{31}P NMR (202 MHz, $CDCl_3$-d_3) of DAD

DAAD 4-mer

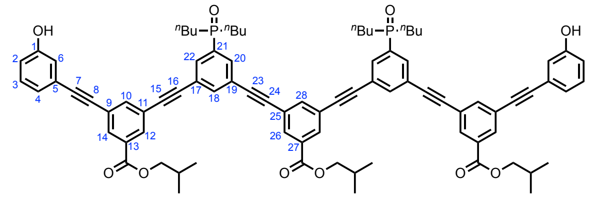

TLC R_f: 0.40 (MeOH:CH_2Cl_2 1:9);

^{1}H NMR (500 MHz, $CDCl_3$): δ 8.14 (d, *J* = 1.5 Hz, 2H, 26-H), 8.09 (t, *J* = 1.5 Hz, 2H, 12-H), 8.06 (t, *J* = 1.5 Hz, 2H, 14-H), 8.04 (bs, 2H, OH), 7.92 – 7.82 (m, 7H, 18, 20, 22, 28-H), 7.81 (t, *J* = 1.5 Hz, 2H, 10-H), 7.24 (t, *J* = 8.0 Hz, 2H, 3-H), 7.17 (dd, *J* = 2.5, 1.5 Hz, 2H, 6-H), 7.09 (dt, *J* = 7.5, 1.0 Hz, 2H, 4-H), 6.93 (ddd, *J* = 8.0, 2.5, 1.0 Hz, 2H, 2-H), 4.15 (d, *J* = 6.5 Hz, 2H, ^{i}Bu), 4.12 (d, *J* = 6.5 Hz, 4H, ^{i}Bu), 2.14 – 2.10 (m, 3H, ^{i}Bu), 2.07-1.81 (m, 8H, ^{n}Bu), 1.71-1.55 (m, 4H, ^{n}Bu), 1.49-1.35 (m, 12H, ^{n}Bu), 1.06 (d, J = 6.5 Hz, 6H, ^{i}Bu), 1.04 (d, J = 6.5 Hz, 12H, ^{i}Bu), 0.90 (t, *J* = 7.0 Hz, 12H, ^{n}Bu);

^{13}C NMR (126 MHz, $CDCl_3$): δ 165.2 (13-C=O), 165.0 (27-C=O), 156.8 (1-C), 138.7 (28-C), 138.5 (10-C), 137.6 (18-C), 133.7 (d, *J* = 88.0 Hz, 21-C), 133.1 (22-C), 133.0 (20-C), 132.5 (12-C), 131.8 (14-C), 131.4 (26-C), 131.2 (13-C), 129.6 (3-C), 124.4 (4-C), 124.2 (d, J = 13.0 Hz, 17-C), 124.0 (d, *J* = 13.0 Hz, 19-C), 123.5 (11-C), 123.4 (25-C), 123.4 (5-C) 123.1 (9-C), 118.7 (6-C), 116.8 (2-C), 91.3 (-C≡), 90.0 (-C≡), 89.7 (-C≡), 89.1 (-C≡), 88.7 (-C≡), 87.0 (-C≡), 71.7 (^{i}Bu), 71.6 (^{i}Bu), 29.2 (d, *J* = 68.5 Hz, ^{n}Bu), 27.9 (^{i}Bu), 24.1 (d, *J* = 14.5 Hz, ^{n}Bu), 23.5 (d, *J* = 4.0 Hz, ^{n}Bu) 19.3 (^{i}Bu), 19.2 (^{i}Bu), 13.6 (^{n}Bu);

^{31}P NMR (202 MHz, $CDCl_3$-*d*$_3$): δ 42.7 (phosphine oxide)

FT-IR (ATR): 3142 (br), 2926, 2226, 1720, 1592, 1234, 1156, 767 ν_{max}/cm^{-1};

HRMS (ES+): $C_{85}H_{88}O_{10}P_2$ calcd. 1330.5853 found 1330.5850, Δ = -0.20 ppm.

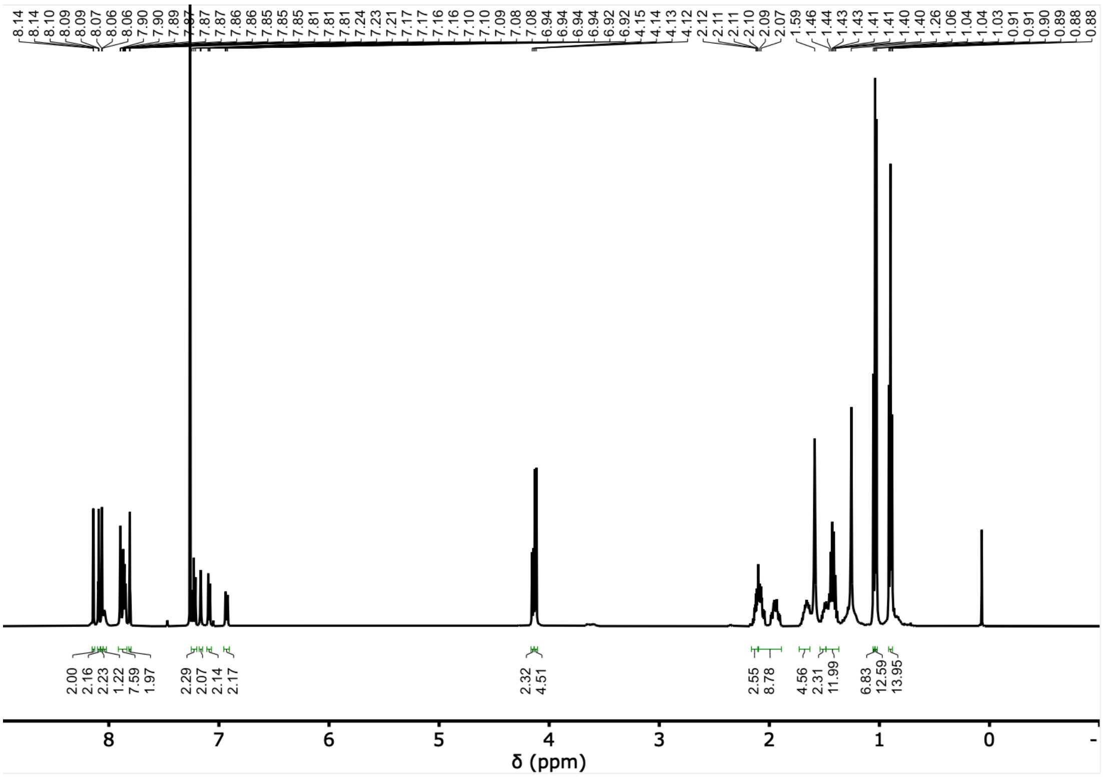

Figure S5 ^{1}H NMR (500 MHz, $CDCl_3$) of DAAD

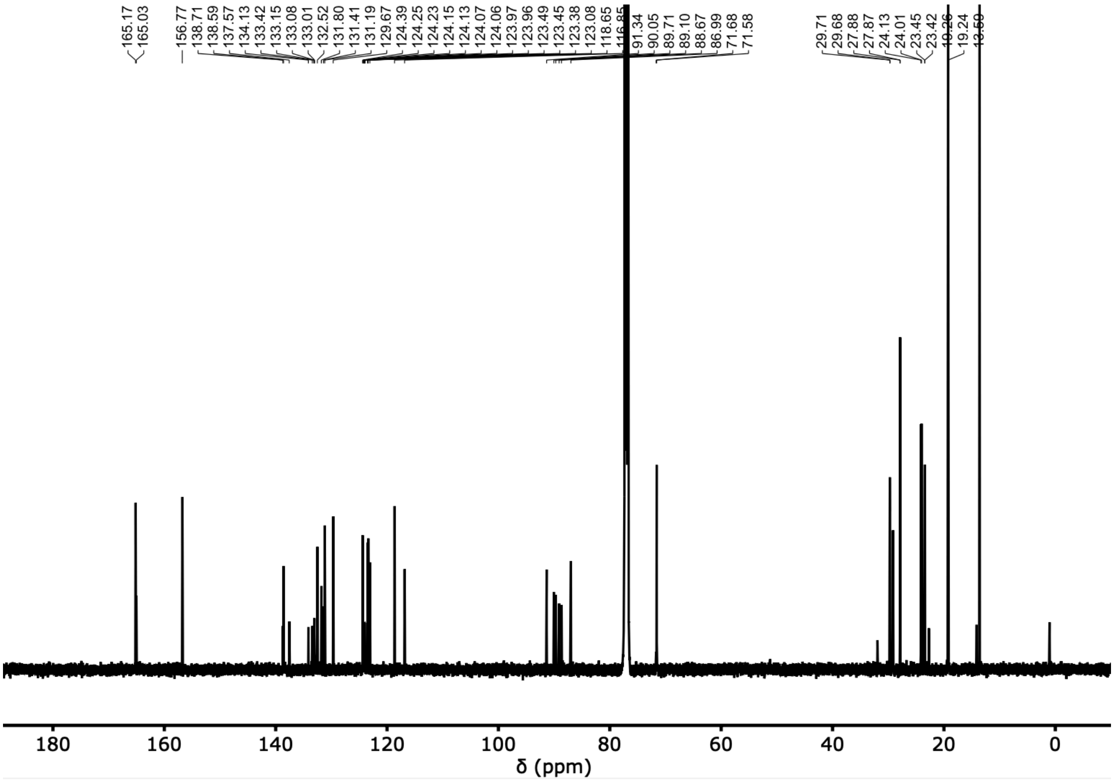

Figure S6 ^{13}C NMR (126 MHz, $CDCl_3$) of DAAD

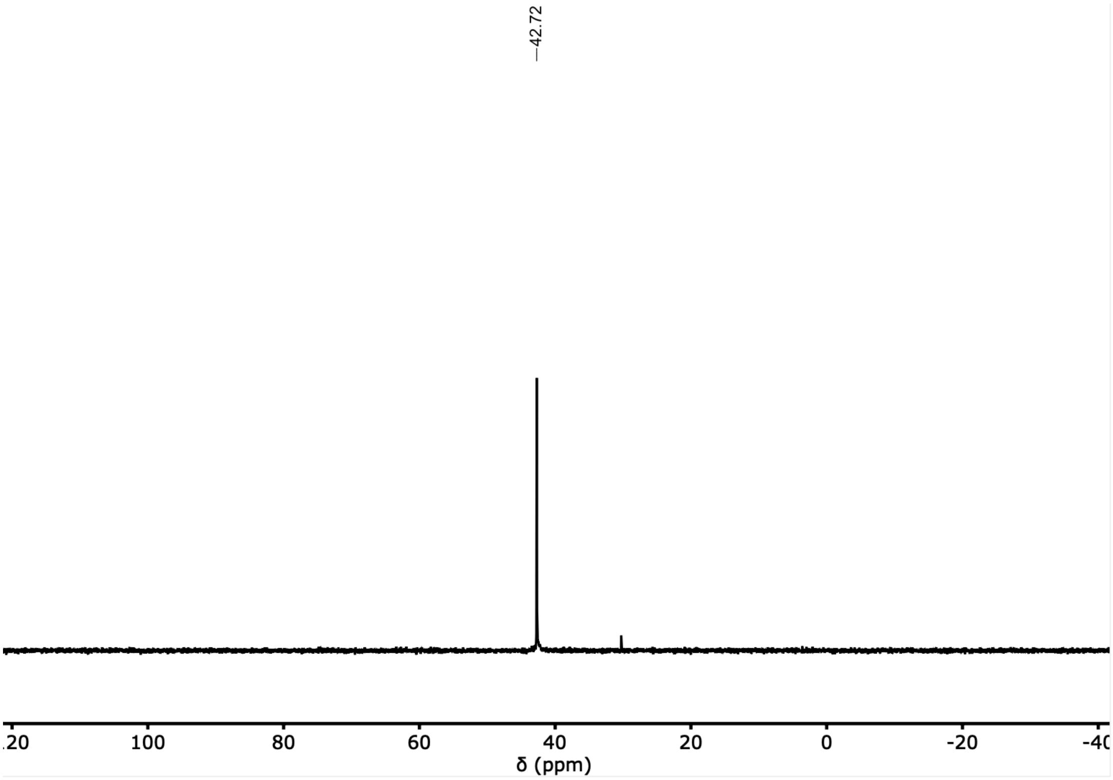

Figure S7 ^{31}P NMR (202 MHz, $CDCl_3$-d_3) of DAAD

DAAAD 5-mer

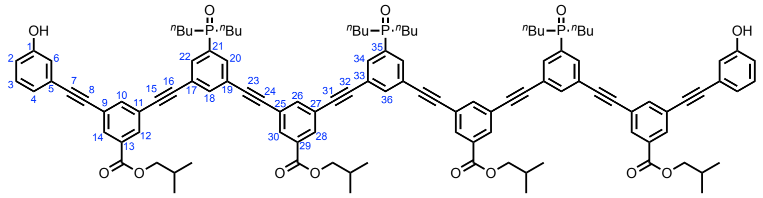

TLC R_f: 0.40 (MeOH:CH_2Cl_2 1:9);

^{1}H NMR (500 MHz, $CDCl_3$): δ 8.18 (dt, J = 6.0, 1.5 Hz, 4H, 12, 14-H), 8.09 (d, J = 1.5 Hz, 4H, 28, 30-H), 7.97 – 7.81 (m, 11H, 18, 20, 22, 26, 34, 36-H), 7.79 (t, J = 1.5 Hz, 2H, 10-H), 7.20 (t, J = 8.0 Hz, 2H, 3-H), 7.11 (dd, J = 2.5, 1.5 Hz, 2H, 6-H), 7.06 (dt, J = 7.5, 1.0 Hz, 2H, 4-H), 6.92 (ddd, J = 8.0, 2.5, 1.0 Hz, 2H, 2-H), 4.16 (d, J = 6.5 Hz, 4H, ^{i}Bu), 4.12 (d, J = 6.5 Hz, 4H, ^{i}Bu), 2.14 – 2.10 (m, 4H, ^{i}Bu), 2.07-1.81 (m, 12H, ^{n}Bu), 1.71-1.55 (m, 6H, ^{n}Bu), 1.49-1.35 (m, 18H, ^{n}Bu), 1.06 (d, J = 6.5 Hz, 12H), 1.03 (d, J = 6.5 Hz, 12H, ^{i}Bu), 0.90 (t, J = 7.0 Hz, 18H, ^{n}Bu);

^{13}C NMR (126 MHz, $CDCl_3$): δ 165.2 (13-C=O), 165.1 (29-C=O), 156.8 (1-C), 138.6 (26-C), 138.4 (10-C), 137.6 (18-C), 137.4 (38-C), 133.7 (d, J = 88.0 Hz, 21, 35-C), 133.1 (Ar-C), 133.0 (Ar-C), 132.5 (Ar-C), 132.0 (Ar-C), 131.5 (Ar-C), 131.2 (13-C), 129.6 (3-C), 124.4 (4-C), 124.1 (Ar-C), 124.0 (Ar-C), 124.0 (Ar-C), 123.5 (Ar-C), 123.5 (Ar-C), 123.4 (Ar-C), 123.4 (Ar-C), 123.1 (Ar-C), 118.7 (6-C), 116.9 (2-C), 91.3 (-C≡), 89.9 (-C≡), 89.6 (-C≡), 89.6 (-C≡), 89.1 (-C≡), 88.6 (-C≡), 86.9 (-C≡), 71.7 (^{i}Bu), 71.6 (^{i}Bu), 29.2 (d, J = 68.5 Hz, ^{n}Bu), 27(^{i}Bu), 27.9 (^{i}Bu), 24.1 (d, J = 14.5 Hz, ^{n}Bu), 23.5 (d, J = 4.0 Hz, ^{n}Bu) 19.3 (^{i}Bu), 19.2 (^{i}Bu), 13.6 (^{n}Bu);

^{31}P NMR (202 MHz, $CDCl_3$-d_3): 41.8, 41.5

FT-IR (ATR): 3129 (br), 2958, 2189, 1721, 1691 1594, 1235 ν_{max}/cm^{-1};

HRMS (ES+): $C_{114}H_{121}O_{13}P_3$ calcd. 1790.8020 found 1790.7977, Δ = -2.40 ppm.

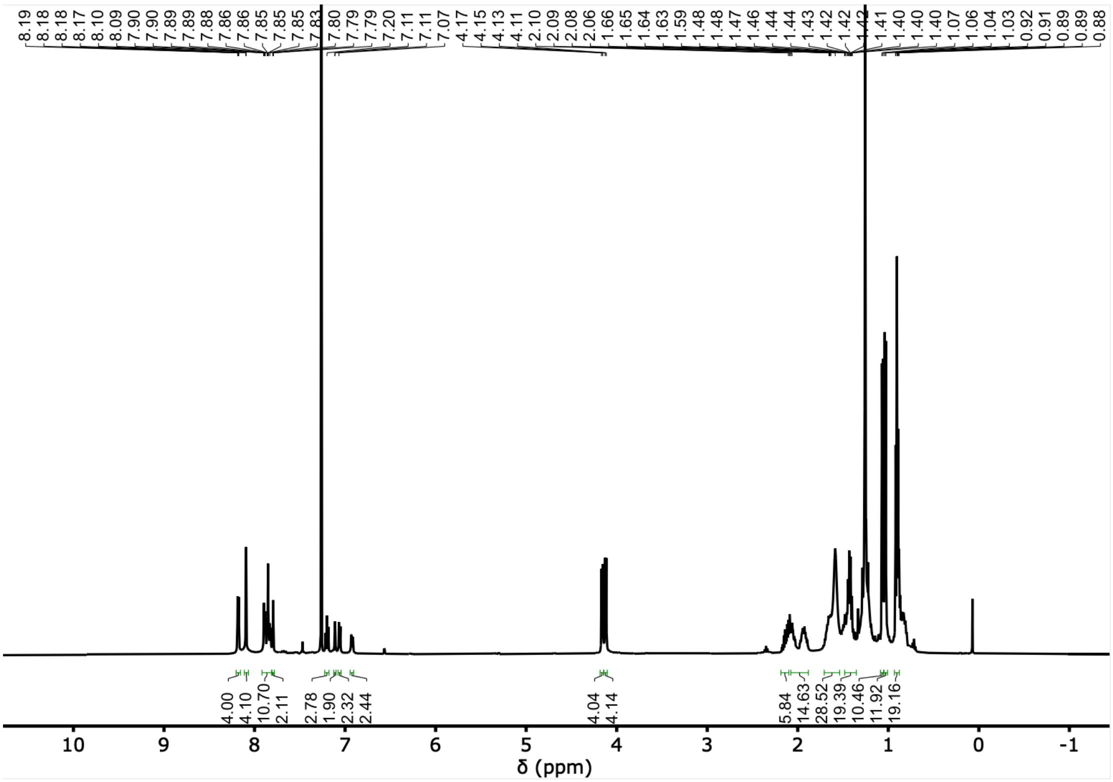

Figure S8 ^{1}H NMR (500 MHz, $CDCl_3$) of DAAAD

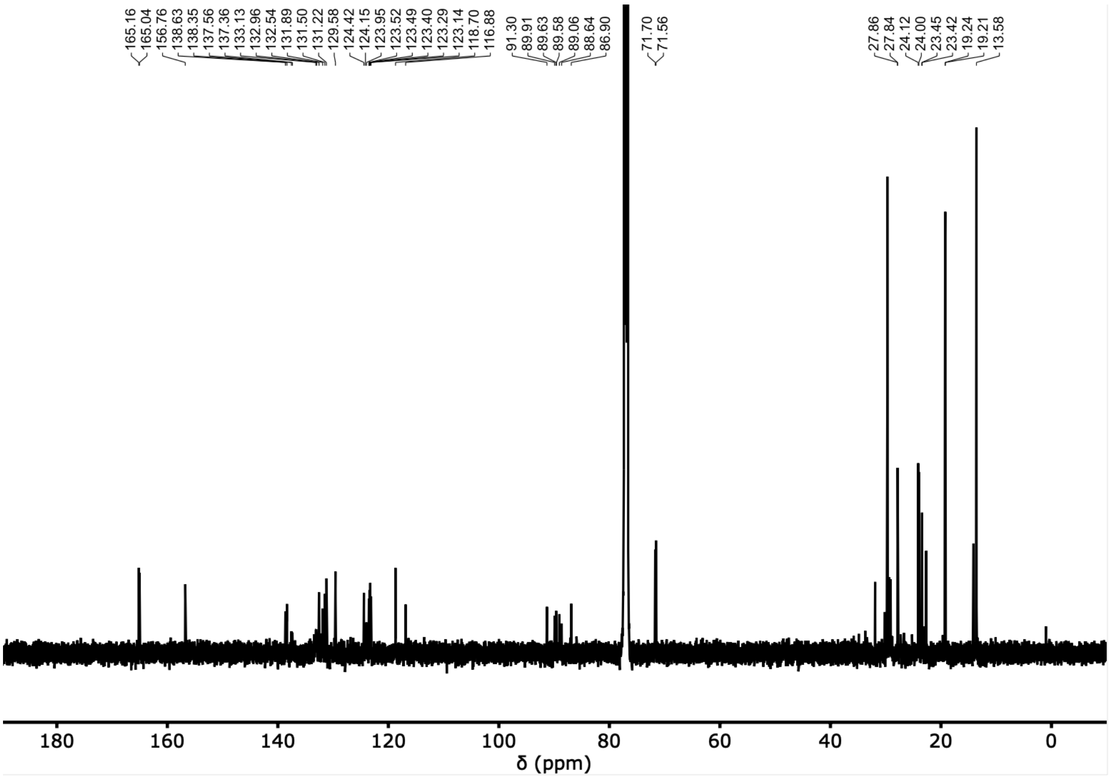

Figure S9 ^{13}C NMR (126 MHz, $CDCl_3$) of DAAAD

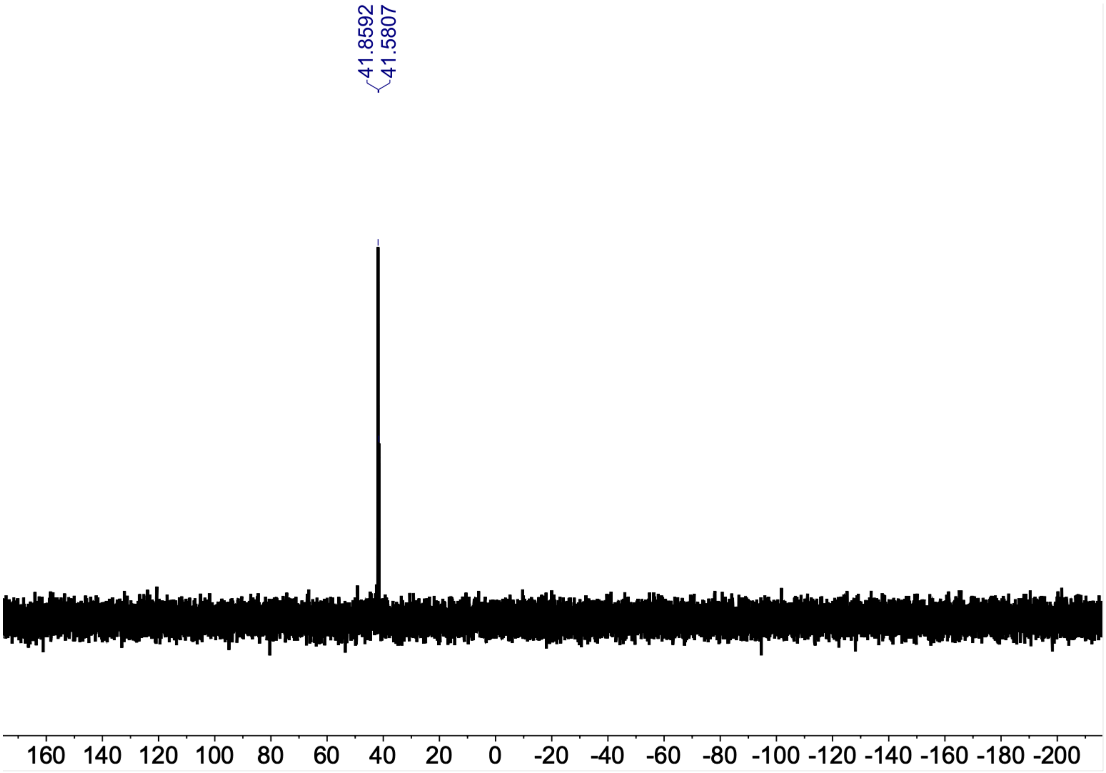

Figure S10 ^{31}P NMR (202 MHz, $CDCl_3$-d_3) of DAAAD

DAAAAD 6-mer

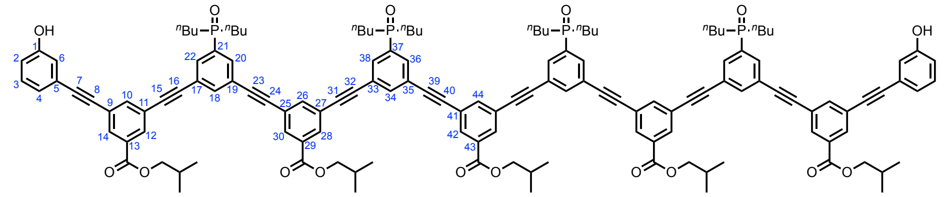

TLC R_f: 0.40 (MeOH:CH_2Cl_2 1:9);

^{1}H NMR (500 MHz, $CDCl_3$): δ 8.50 (bs, 2H, OH), 8.19 (d, J = 1.5 Hz, 2H, 42-H),k 8.17 (dt, J = 6.0, 1.5 Hz, 4H, 12, 14-H), 8.09 (d, J = 1.5 Hz, 4H, 28, 30-H), 7.94 – 7.81 (m, 15H, 18, 20, 22, 26, 34, 36, 38, 44-H), 7.80 (t, J = 1.5 Hz, 2H, 10-H), 7.21 (t, J = 8.0 Hz, 2H, 3-H), 7.15 (dd, J = 2.5, 1.5 Hz, 2H, 6-H), 7.07 (dt, J = 7.5, 1.0 Hz, 2H, 4-H), 6.95 (ddd, J = 8.0, 2.5, 1.0 Hz, 2H, 2-H), 4.33 – 3.93 (m, 10H ^{i}Bu), 2.14 – 2.10 (m, 5H, ^{i}Bu), 2.10-2.05-1.81 (m, 6H, ^{n}Bu), 1.98-1.81-2.05-1.81 (m, 10H, ^{n}Bu), 1.71-1.55 (m, 8H, ^{n}Bu), 1.49-1.35 (m, 24H, ^{n}Bu), 1.08 – 1.01 (m, 30H, ^{i}Bu), 0.97 – 0.79 (m, 24H, ^{n}Bu);

^{13}C NMR (126 MHz, $CDCl_3$): δ 165.2 (13-C=O), 165.1 (29, C=O), 165.1 (43-C=O), 157.00 (1-C), 138.6 (26, 44-C), 138.4 (10-C), 137.5 (18-C), 137.3 (34-C), 133.1 (bs), 132.5 (Ar-C), 132.1 (Ar-C), 132.0 (Ar-C), 131.5 (Ar-C), 131.2 (13-C), 129.5 (3-C), 128.5, 128.4, 124.5 (4-C), 124.0 (bs), 123.5 (Ar-C), 123.5 (Ar-C), 123.1 (Ar-C), 118.7 (6-C), 116.9 (2-C), 91.4 (-C≡), 89.9 (-C≡), 89.6 (-C≡), 89.6 (-C≡), 89.1 (-C≡), 89.0 (-C≡), 88.7 (-C≡), 86.9 (-C≡), 71.7 (^{i}Bu), 71.5 (^{i}Bu), 29.2 (d, J = 68.5 Hz, ^{n}Bu), 27.9 (^{i}Bu), 24.1 (d, J = 14.5 Hz, ^{n}Bu), 23.5 (d, J = 4.0 Hz, ^{n}Bu) 19.3 (^{i}Bu), 19.2 (^{i}Bu), 13.6 (^{n}Bu);

^{31}P NMR (202 MHz, $CDCl_3$-*d_3*): δ 41.4, 41.0 (phosphine oxide)

FT-IR (ATR): 3187 (br), 2958, 2172, 1722, 1594, 1236 ν_{max}/cm^{-1};

HRMS (ES+): $C_{143}H_{154}O_{16}P_4$ calcd. 2251.0187 found 2251.0203, Δ = -0.70 ppm.

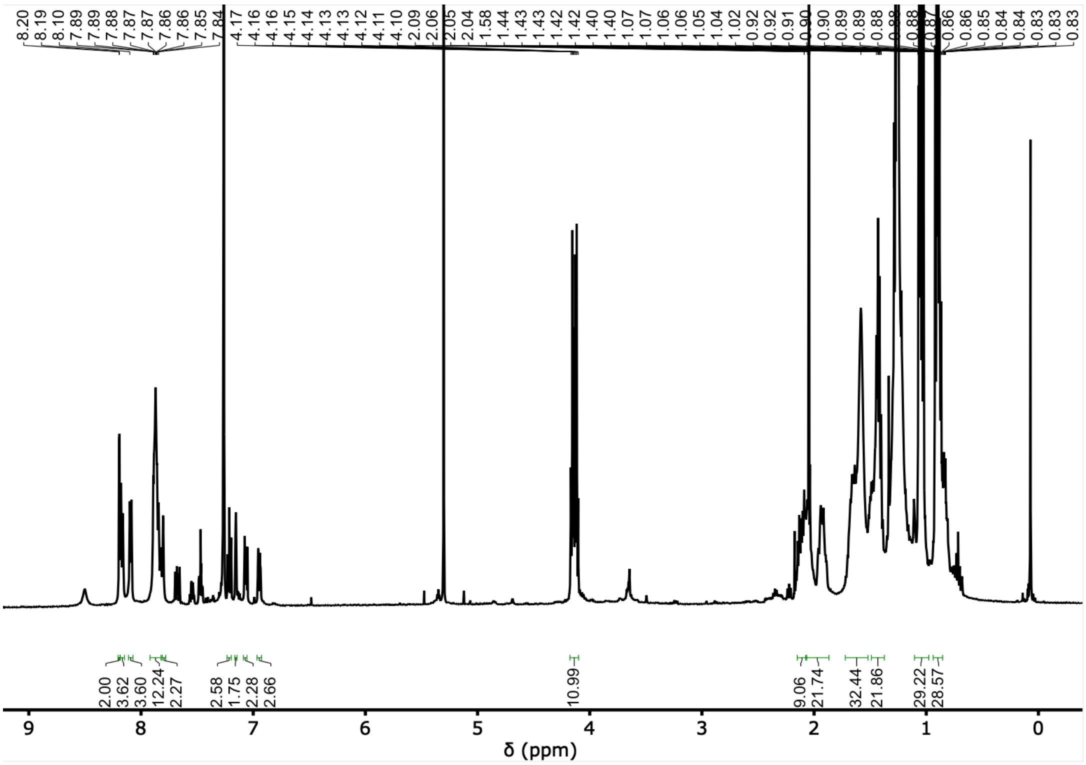

Figure S11 ^{1}H NMR (500 MHz, $CDCl_3$) of DAAAAD

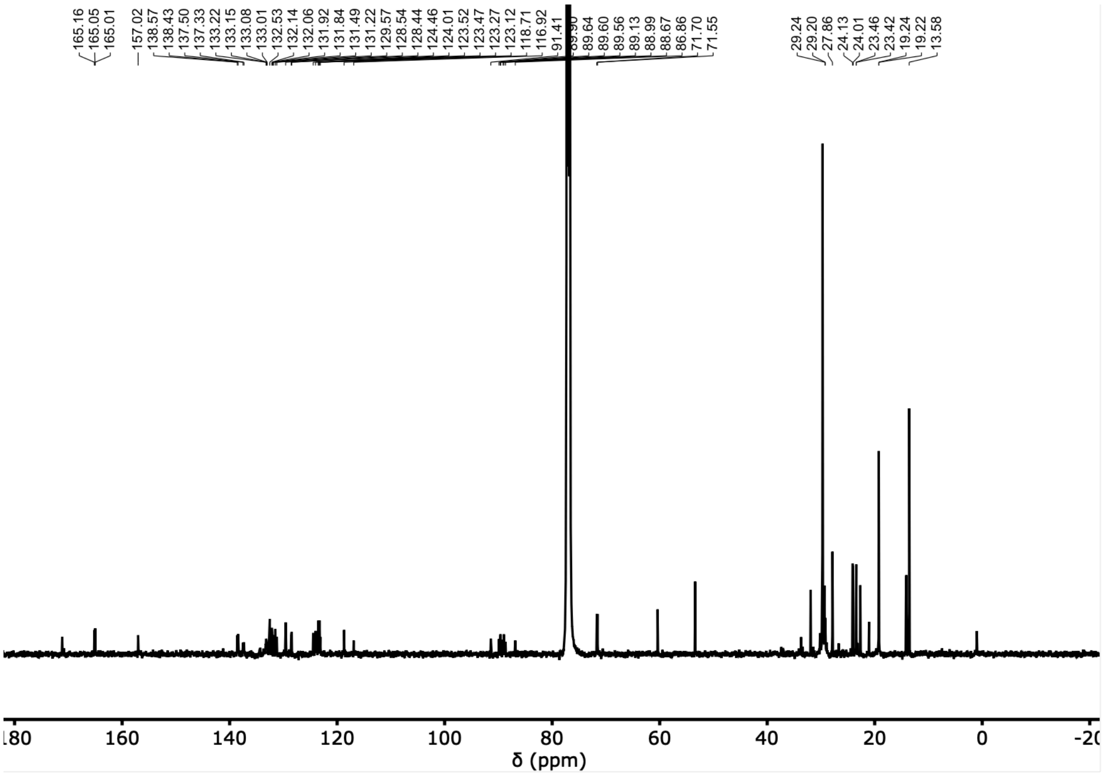

Figure S12 ^{13}C NMR (126 MHz, $CDCl_3$) of DAAAAD

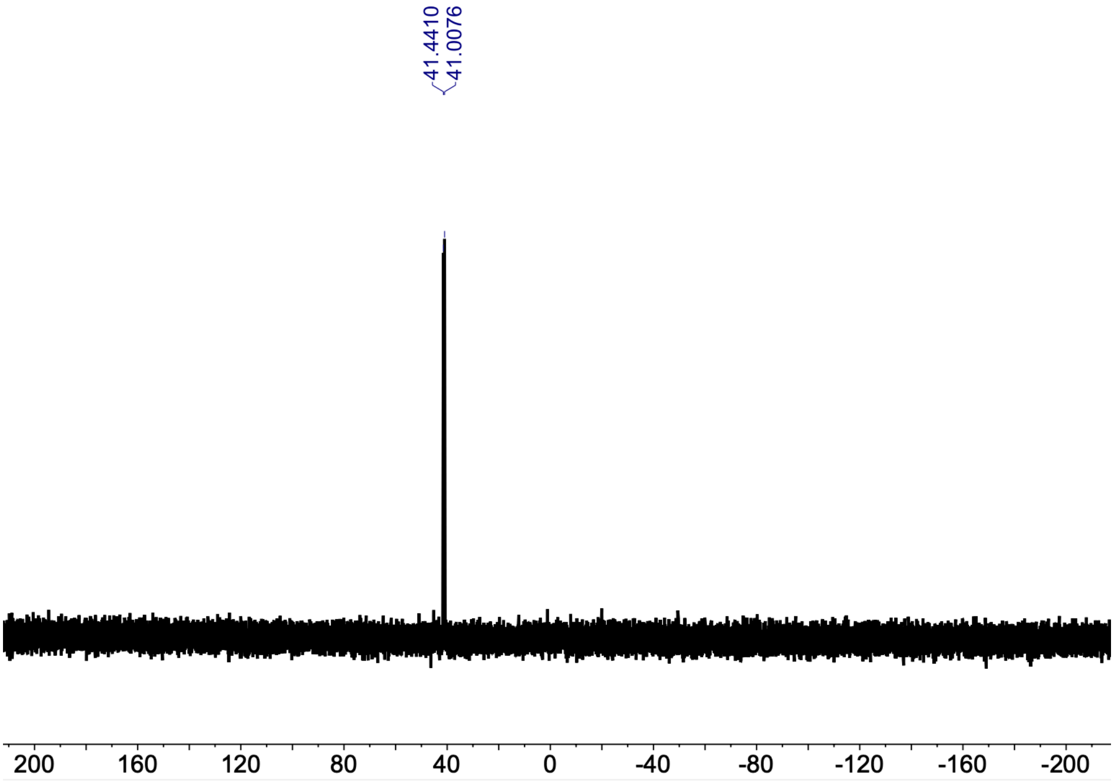

Figure S13 ^{31}P NMR (202 MHz, $CDCl_3$-d_3) of DAAAAD

ADA oligomerization

4 (105 mg, 0.400 mmol), **5** (28.4 mg, 0.200 mmol) and **2** (172 mg, 0.400 mmol) were placed in a flask and degassed with N_2 for 30 minutes. $Pd_2(dba)_3$ (7.30 mg, 8.00 μmol) and CuI (1.50 mg, 8.00 μmol) and PPh_3 (10.5 mg, 40.0 μmol) were placed in a separate flask and degassed with N_2. Degassed Et_3N (167 μL, 1.20 mmol) was added and the contents of this flask transferred to the first using degassed toluene (8 mL). The reaction was stirred overnight at room temperature, in the dark under N_2. The solvent was removed by rotary evaporation under reduced pressure yielding a brown solid (415 mg). The sample was dissolved in EtOH and sonicated, before filtering. Preparative HPLC separation of the oligomerisation mixture was completed using a HIRBP-6988 prep column (250 x 25 mm, 5 μm particle size) with a water/THF solvent system (THF: 57% for 100 mins, 100% for 5 mins) at 15 mL min^{-1}.

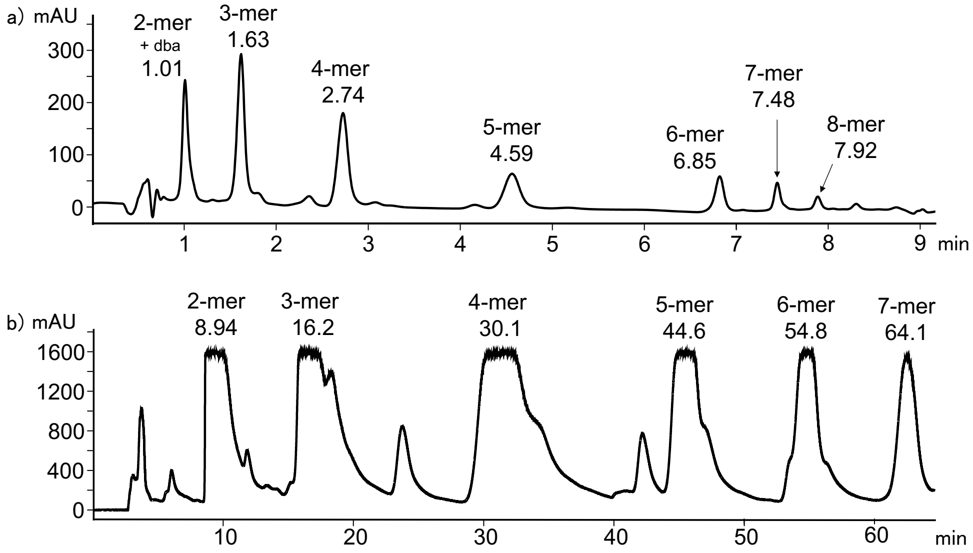

Figure S14: (a) LCMS analysis of AD_nA oligomerisation mixture using a Hichrom C_8C_{18} column (50 x 4.6 mm, 5 μm particle size) with a water/THF (0.1% formic acid) solvent system (THF: 60% for 5 mins, 60-70% over 1 min, 70% for 2 mins) at a flow rate of 1 mL min^{-1}. (b) Preparative HPLC (bottom) separation of AD_nA oligomerisation mixture using a HIRBP-6988 prep column (250 x 25 mm, 5 μm particle size) with a water/THF solvent system (THF: 60% for 36 mins, 65% for 28 mins, 70% for 15 mins) at 15 mL min^{-1}. Both samples were prepared in EtOH. UV/vis absorption was measured at 290 nm. Peaks identified by MS are labelled with retention time in minutes; dba = dibenzylideneacetone.

ADA 3-mer

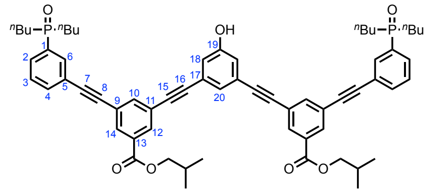

TLC R_f: 0.40 (MeOH:CH_2Cl_2 1:9);

^{1}H NMR (500 MHz, THF-d_8): δ 10.10 (bs, 1H, OH), 8.13 (m, 4H, 12, 14-H), 8.05 (d, J = 10.5 Hz, 2H, 6-H), 7.92 (m, 2H, 10-H), 7.85 (dd, J = 10.0, 7.5 Hz, 2H, 2-H), 7.79 – 7.73 (m, 2H, 4-H), 7.59 (td, J = 7.5, 2.5 Hz, 2H, 3-H), 7.31 (t, J = 1.5 Hz, 1H, 20-H), 7.17 (d, J = 1.5 Hz, 2H, 18-H), 4.17 (d, J = 6.5 Hz, 4H, ^{i}Bu), 2.14 – 2.10 (m, 2H, ^{i}Bu), 2.07-1.81 (m, 8H, ^{n}Bu), 1.71-1.55 (m, 4H, ^{n}Bu), 1.49-1.35 (m, 12H, ^{n}Bu), 1.06 (d, J = 6.5 Hz, 12H, ^{i}Bu), 0.91 (t, J = 7.0 Hz, 12H, ^{n}Bu);

^{13}C NMR (126 MHz, THF-d_8): δ 164.1 (C=O), 158.5 (19-C), 138.0 (10-C), 134.9 (d, J = 88.5 Hz, 1-C), 134.0 (4-C), 133.7 (d, J = 9.0 Hz, 6-C), 131.8 (Ar-C), 131.6 (12-C), 131.6 (14-C), 130.8 (d, J = 8.5 Hz, 2-C), 128.6 (d, J = 11.0 Hz, 3-C), 125.5 (20-C), 124.1 (Ar-C), 123.8 (Ar-C), 123.7 (Ar-C), 123.0 (d, J = 12.0 Hz, 5-C), 119.3 (18-C), 90.3 (-C≡), 90.0 (-C≡), 88.2 (-C≡), 87.1 (-C≡), 71.1 (^{i}Bu), 29.3 (d, J = 68.5 Hz, ^{n}Bu), 27.9 (^{i}Bu), 24.1 (d, J = 14.5 Hz, ^{n}Bu), 23.5 (d, J = 4.0 Hz, ^{n}Bu) 19.3 (^{i}Bu), 13.6 (^{n}Bu);

^{31}P NMR (202 MHz, $CDCl_3$-d_3): δ 41.3 (phosphine oxide);

FT-IR (ATR): 3060 (br), 2958, 2164, 1722, 1594, 1159, 768 ν_{max}/cm^{-1};

HRMS (ES+): $C_{64}H_{73}O_7P_2$ calcd. 1015.4826 found 1015.4798, Δ = -2.82 ppm.

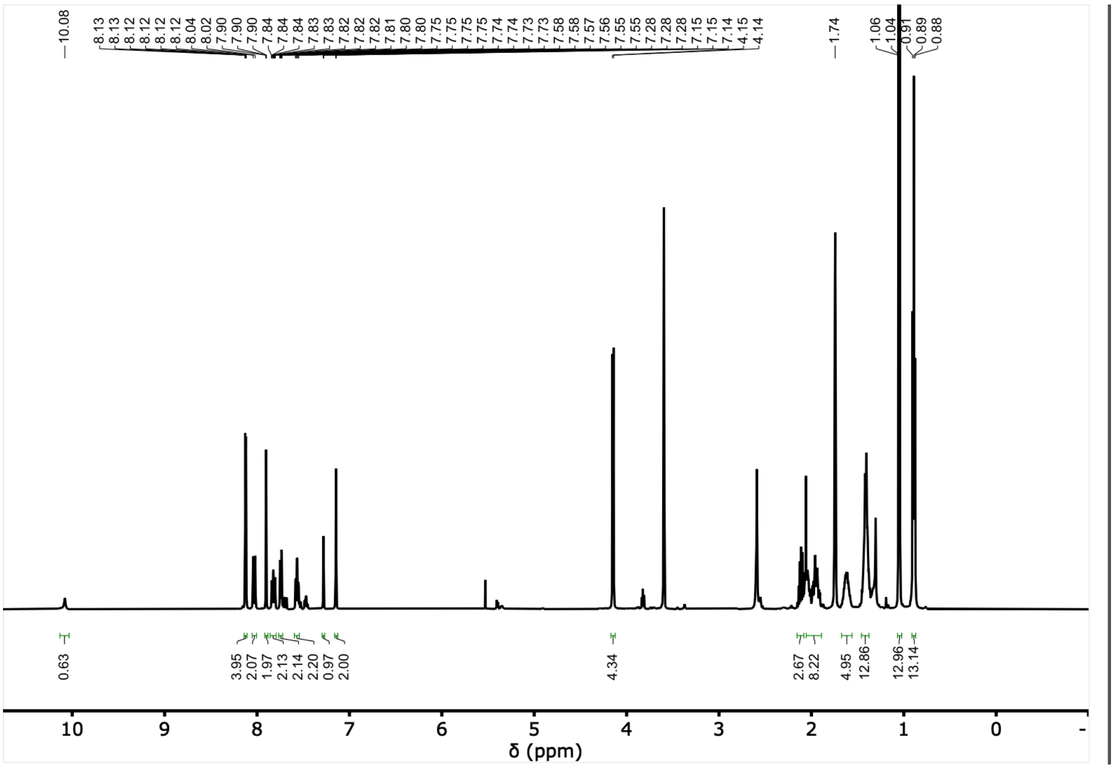

Figure S15 ^{1}H NMR (500 MHz, THF-d_8) of ADA

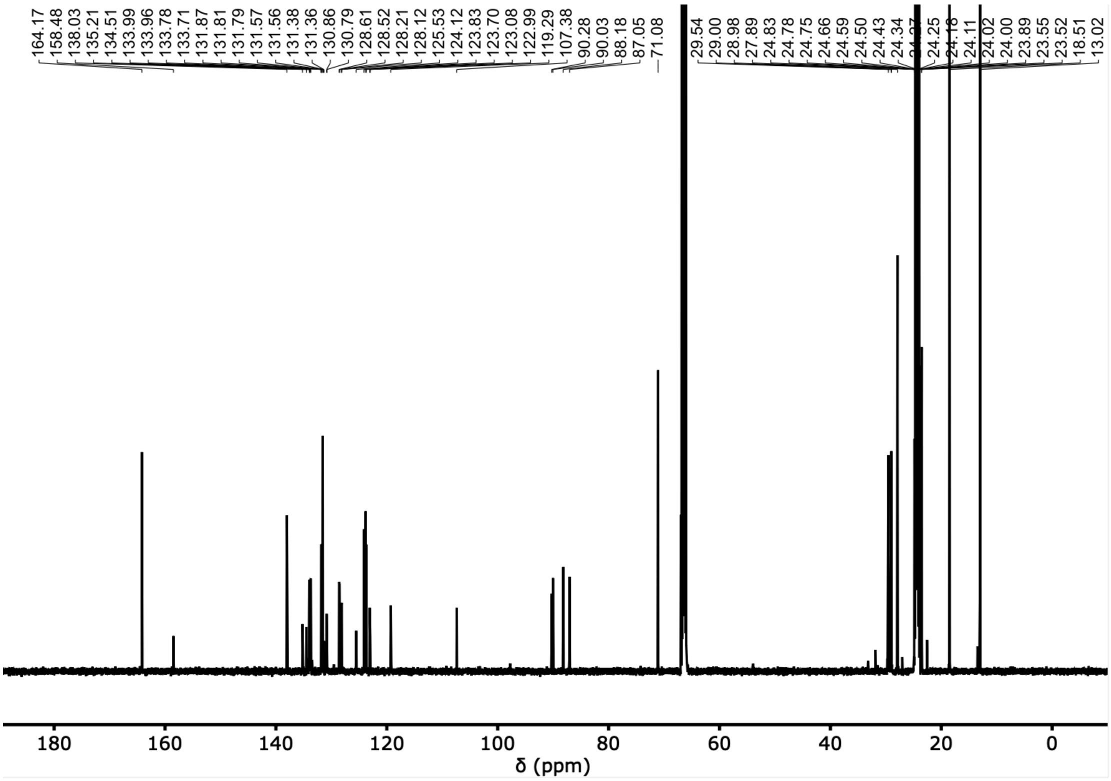

Figure S16 ^{13}C NMR (126 MHz, THF-d_8) of ADA

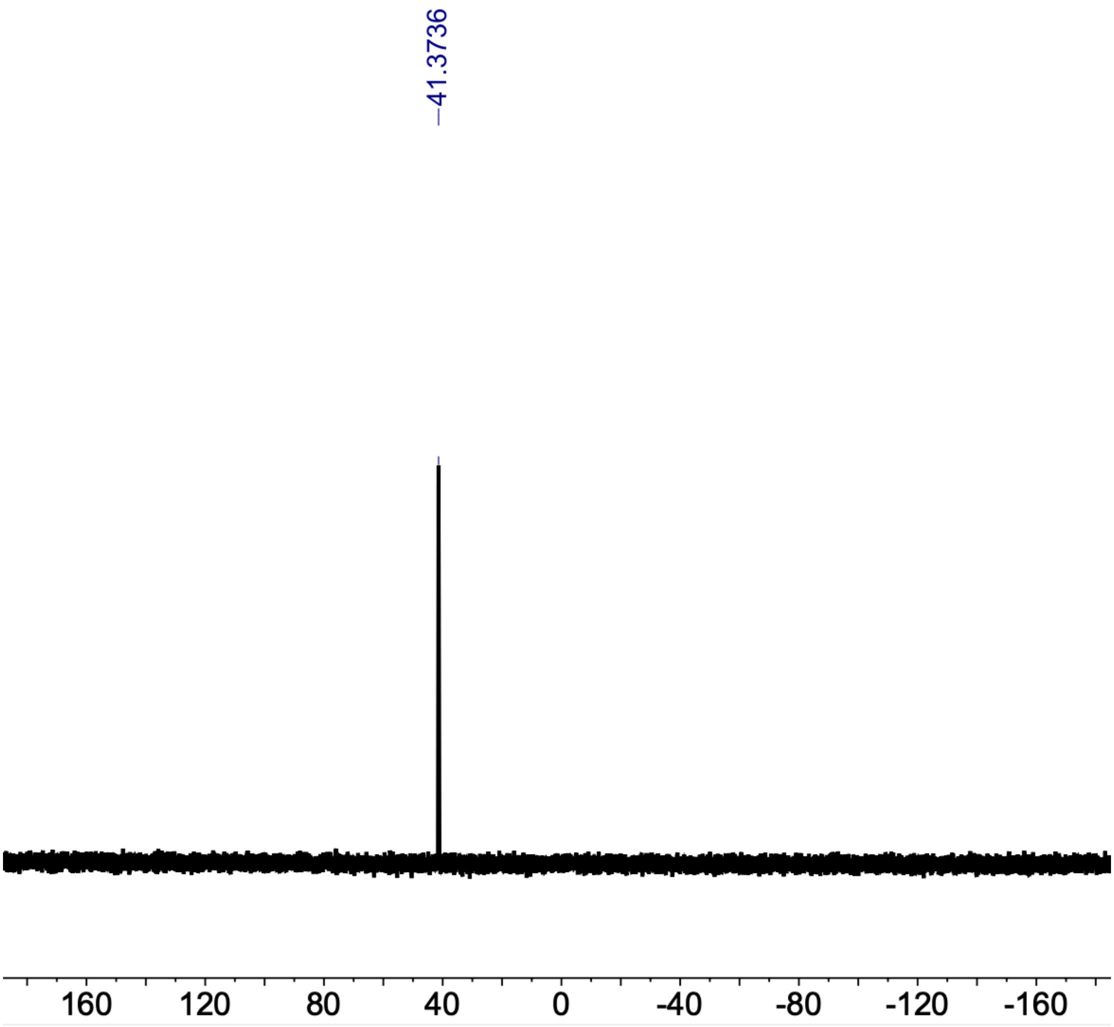

Figure S17 ^{31}P NMR (202 MHz, $CDCl_3$-d_3) of ADA

ADDA 4-mer

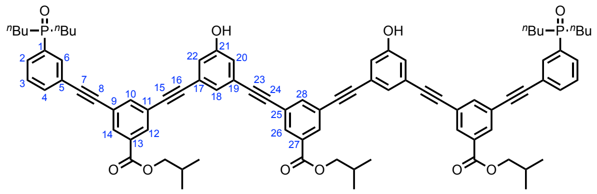

TLC R_f: 0.40 (MeOH:CH_2Cl_2 1:9);

^{1}H NMR (500 MHz, THF-d_8): δ 9.85 (bs, 2H, OH), 8.15 (m, 6H, 12, 14, 26-H), 8.07 (d, J = 11.0 Hz, 2H, 6-H), 7.94 (m, 3H, 10, 28-H), 7.84 (m, 2H, 2-H), 7.80 – 7.74 (m, 2H, 4-H), 7.59 (td, J = 7.5, 2.5 Hz, 2H, 3-H), 7.32 (t, J = 1.5 Hz, 2H, 18-H), 7.12 (d, J = 1.5 Hz, 4H, 20, 22-H), 4.17 (m, 6H, ^{i}Bu), 2.14 – 2.10 (m, 3H, ^{i}Bu), 2.07-1.81 (m, 8H, ^{n}Bu), 1.71-1.55 (m, 4H, ^{n}Bu), 1.49-1.35 (m, 12H, ^{n}Bu), 1.07 (d, J = 6.5 Hz, 6H, ^{i}Bu), 1.06 (d, J = 6.5 Hz, 12H, ^{i}Bu), 0.91 (t, J = 7.0 Hz, 12H, ^{n}Bu);

^{13}C NMR (126 MHz, THF-d_8): δ 166.1 (27-C=O), 166.0 (13-C=O), 160.2 (21-C), 140.0 (10-C), 136.6 (d, J = 88.5 Hz, 1-C), 135.9 (4-C), 135.6 (d, J = 9.0 Hz, 6-C), 133.6 (Ar-C), 133.4 (12-C), 133.4 (14-C), 132.6 (d, J = 8.5 Hz, 2-C), 130.5 (d, J = 11.0 Hz, 3-C), 127.7 (Ar-C), 126.0 (Ar-C), 125.9 (Ar-C), 125.7 (Ar-C), 125.6 (Ar-C), 125.6 (Ar-C), 125.0 (d, J = 12.0 Hz, 5-C), 121.0 (18-C), 92.1 (-C≡), 92.0 (-C≡), 91.9 (-C≡), 90.1 (-C≡), 89.1 (-C≡), 89.0 (-C≡), 73.0 (^{i}Bu), 29.3 (d, J = 68.5 Hz, ^{n}Bu), 26.8 (^{i}Bu), 25.8 (d, J = 14.5 Hz, ^{n}Bu), 25.3 (d, J = 4.0 Hz, ^{n}Bu) 20.3 (^{i}Bu), 14.9 (^{n}Bu);

^{31}P NMR (202 MHz, $CDCl_3$-d_3): δ 43.5 (phosphine oxide);

FT-IR (ATR): 3074 (br), 2961, 2222, 1722, 1594, 1256, 1236 ν_{max}/cm^{-1};

HRMS (ES+): $C_{85}H_{89}O_{10}P_2$ calcd. 1331.5926 found 1331.5880, Δ = -3.39 ppm.

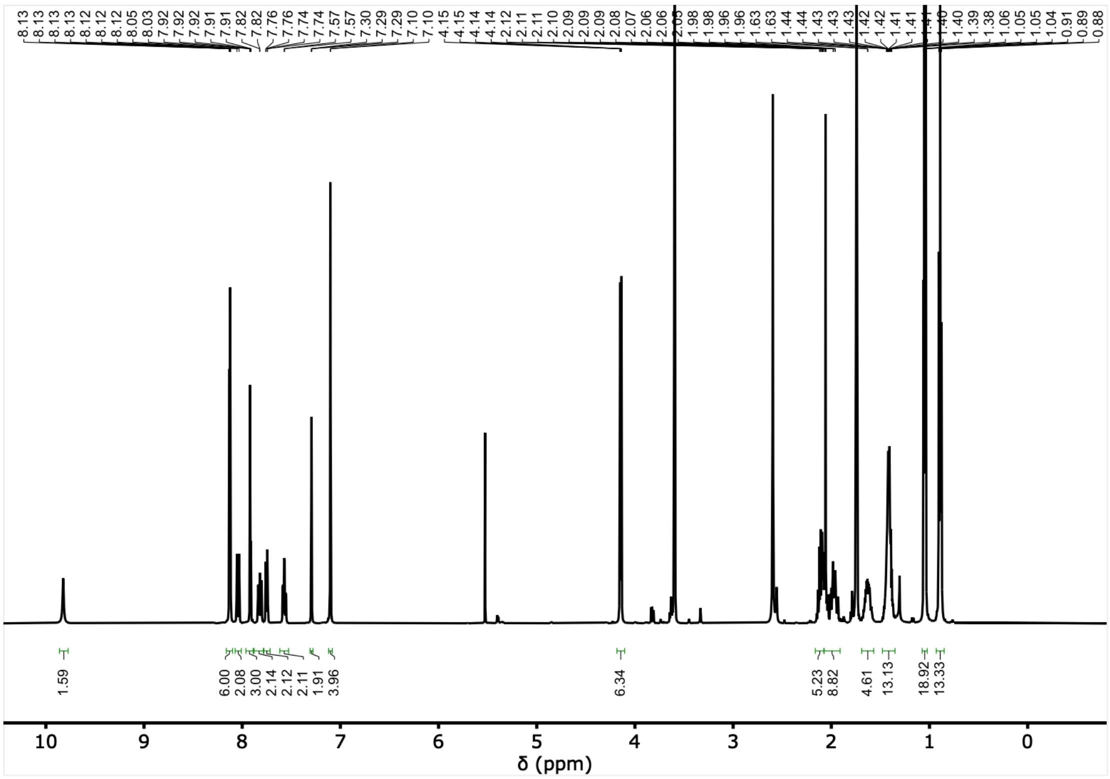

Figure S18 ^{1}H NMR (500 MHz, THF-d_8) of ADDA

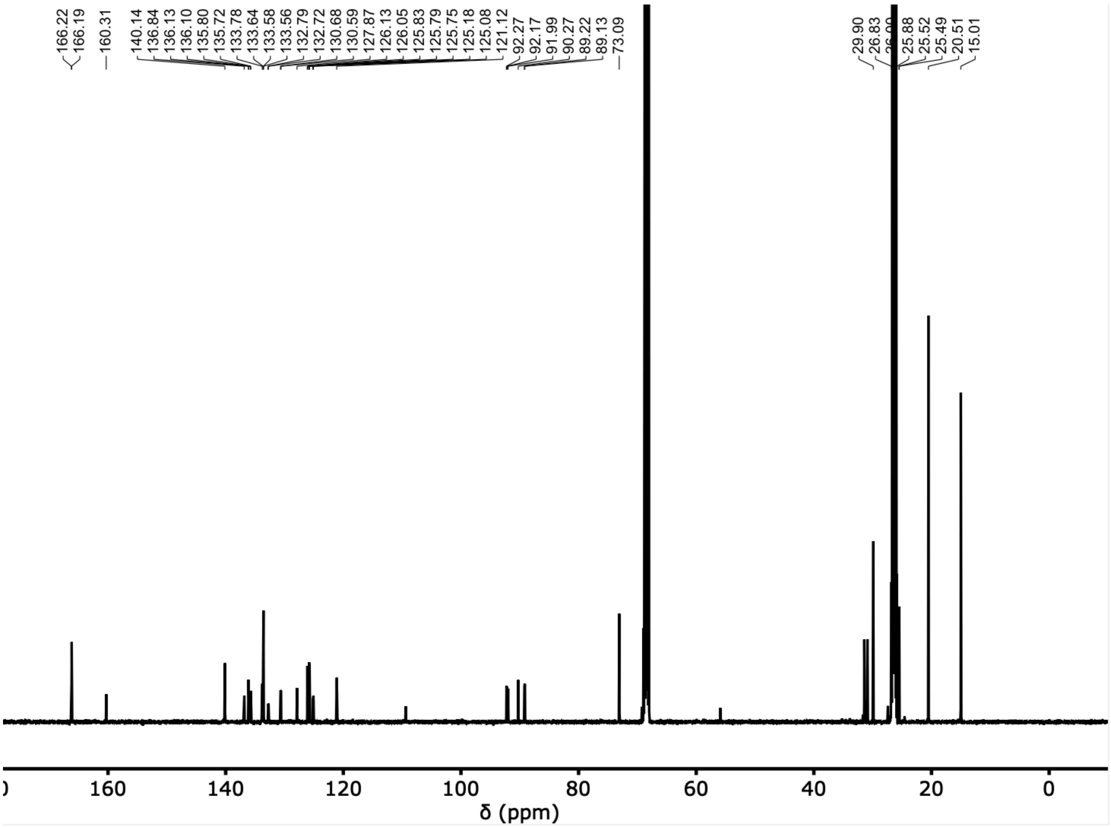

Figure S19 ^{13}CNMR (202 MHz, THF-d_8) of ADDA

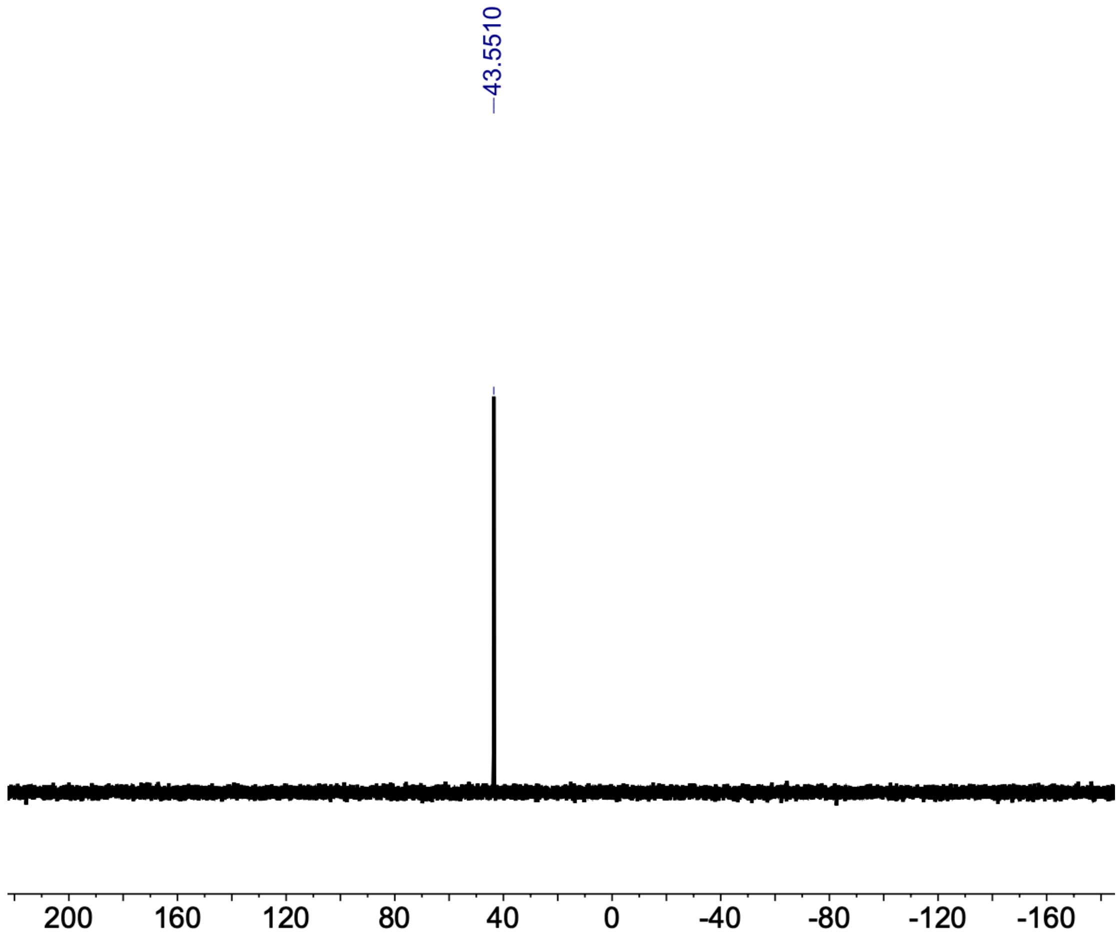

Figure S20 ^{31}P NMR (202 MHz, $CDCl_3$-d_3) of ADDA

ADDDA 5-mer

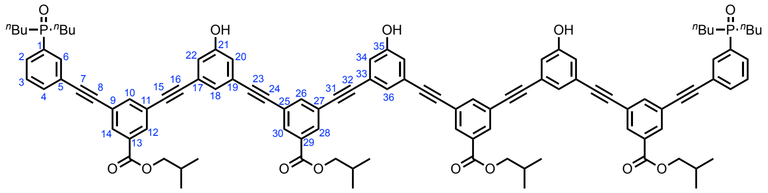

TLC R_f: 0.40 (MeOH:CH_2Cl_2 1:9);

^{1}H NMR (500 MHz, THF-d_8): δ 9.56 (bs, 3H, OH), 8.16 – 8.11 (m, 8H, 12, 14, 28, 30-H), 8.03 (dt, J = 11.0, 1.5 Hz, 2H, 6-H), 7.92 (t, J = 1.5 Hz, 4H, 10, 26-H), 7.82 (ddt, J = 10.5, 7.5, 1.5 Hz, 2H, 2-H), 7.75 (d, J = 8.0 Hz, 2H, 4-H), 7.57 (td, J = 7.5, 2.5 Hz, 2H, 3-H), 7.28 (dt, J = 2.5, 1.5 Hz, 3H, 18, 36-H), 7.07 (m, 6H, 20, 22, 34-H), 4.15 (d, J = 6.5 Hz, 4H, ^{i}Bu), 4.14 (d, J = 6.5 Hz, 4H, ^{i}Bu), 2.14 – 2.10 (m, 4H, ^{i}Bu), 2.07-1.81 (m, 8H, ^{n}Bu), 1.71-1.55 (m, 4H, ^{n}Bu), 1.49-1.35 (m, 12H, ^{n}Bu), 1.06 (d, J = 6.5 Hz, 12H, ^{i}Bu), 1.04 (d, J = 6.5 Hz, 12H, ^{i}Bu), 0.91 (t, J = 7.0 Hz, 12H, ^{n}Bu);

^{13}C NMR (126 MHz, THF-d_8): δ 166.1 (29-C=O), 166.0 (13-C=O), 160.1 (21-C), 160.0 (35-C), 140.0 (10-C), 139.9 (26-C), 136.3 (d, J = 88.5 Hz, 1-C), 135.9 (4-C), 135.6 (d, J = 9.0 Hz, 6-C), 133.7 (Ar-C), 133.6 (Ar-C), 133.6 (Ar-C), 133.4 (Ar-C), 133.4 (Ar-C), 132.6 (d, J = 8.5 Hz, 2-C), 130.5 (d, J = 11.0 Hz, 3-C), 127.7 (20-C), 127.6 (Ar-C), 126.0 (Ar-C), 125.9 (Ar-C), 125.7 (Ar-C), 125.7 (Ar-C), 125.7 (Ar-C), 125.7 (Ar-C), 121.1 (36-C), 121.0 (18-C), 92.1 (-C≡), 92.1 (-C≡), 92.0 (-C≡), 91.9 (-C≡), 90.1 (-C≡), 89.1 (-C≡), 89.0 (-C≡), 89.0 (-C≡), 73.0 (^{i}Bu), 73.0 (^{i}Bu), 29.3 (d, J = 68.5 Hz, ^{n}Bu), 27.9 (^{i}Bu), 24.1 (d, J = 14.5 Hz, ^{n}Bu), 25.3 (d, J = 4.0 Hz, ^{n}Bu) 20.3 (^{i}Bu), 14.9 (^{n}Bu);

^{31}P NMR (162 MHz, $CDCl_3$): δ 44.3

FT-IR (ATR): 3013 (br), 2961, 2203, 1723, 1593, 1236, 768 ν_{max}/cm^{-1};

HRMS (ES+): $C_{106}H_{106}O_{13}P_2$ calcd. 1648.7103 found 1648.7030, Δ = -4.47 ppm.

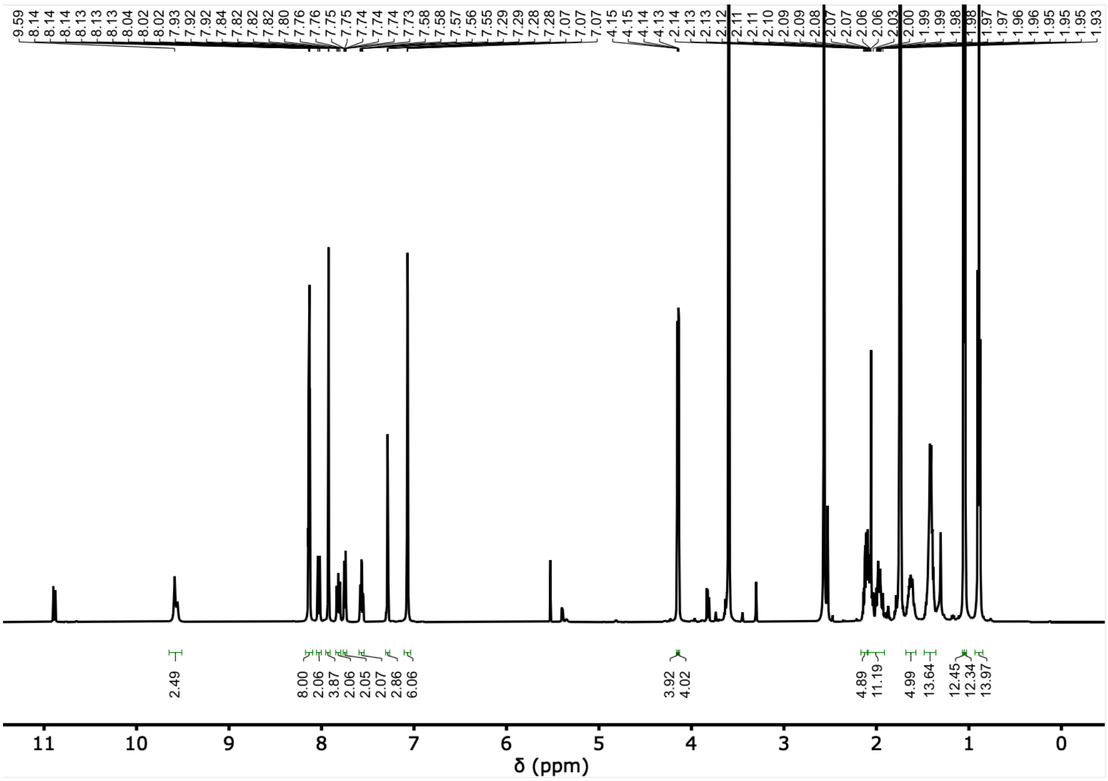

Figure S21 ^{1}H NMR (500 MHz, THF-d_8) of ADDDA

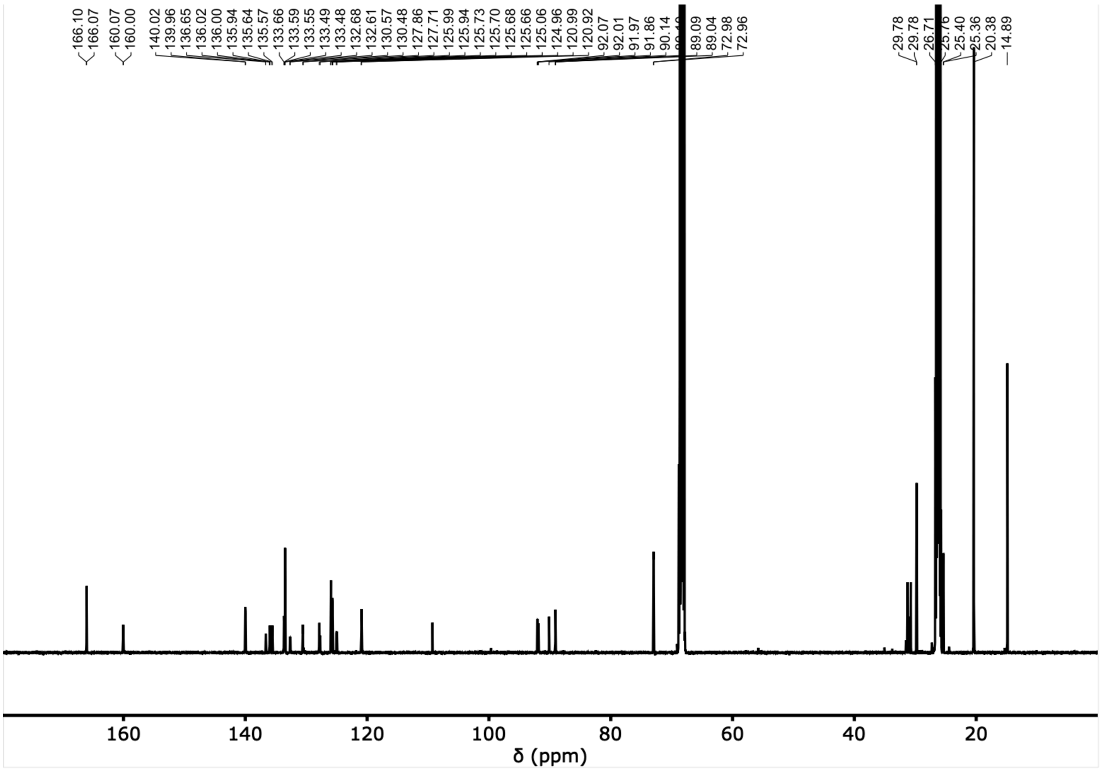

Figure S22 ^{13}C NMR (126 MHz, THF-d_8) of ADDDA

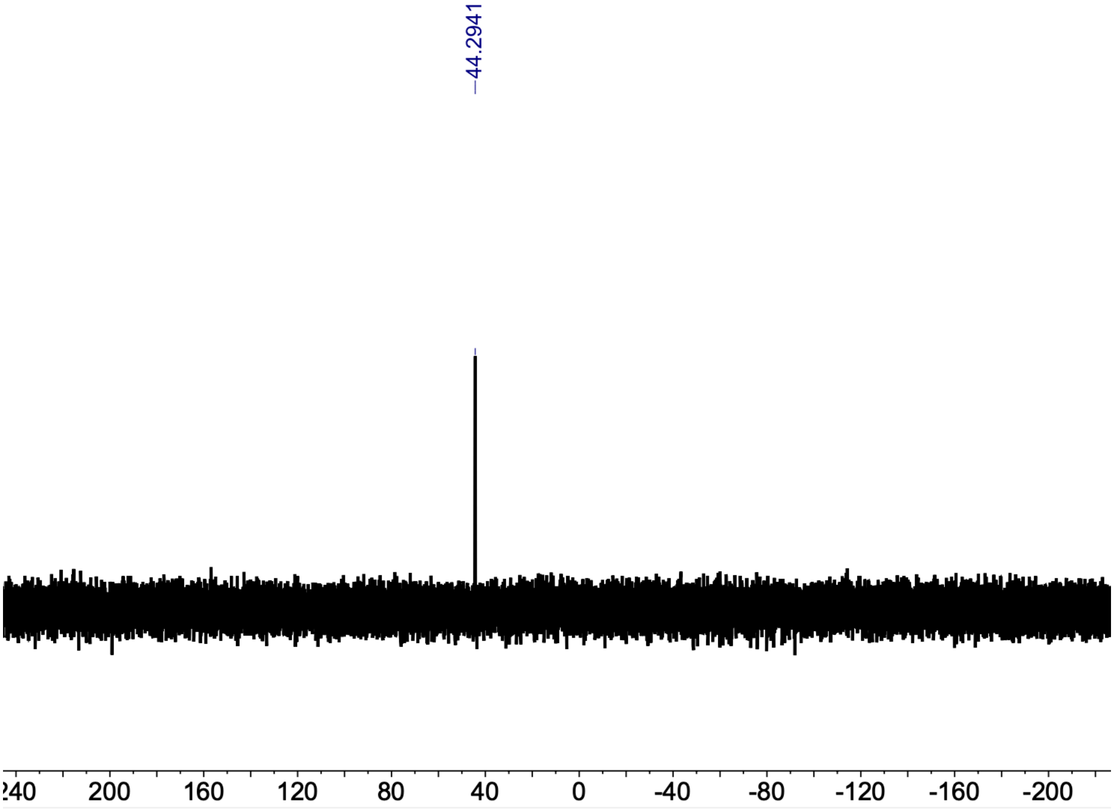

Figure S23 ^{31}P NMR (162 MHz, $CDCl_3$) of ADDDA

ADDDDA 6-mer

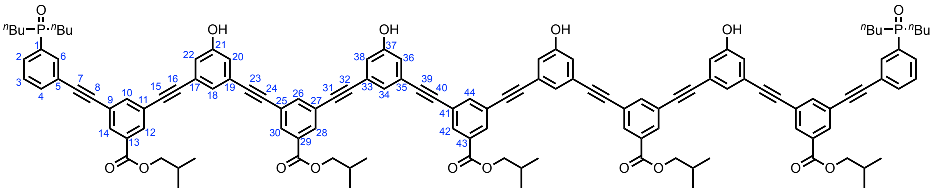

TLC *Rf*: 0.40 (MeOH:CH_2Cl_2 1:9);

^{1}H NMR (500 MHz, THF-d_8): δ 9.51 (bs, 4H, OH), 8.16 – 8.12 (m, 10H, 12, 14, 28, 30, 42-H), 8.02 (dt, J = 11.0, 1.5 Hz, 2H, 6-H), 7.95 – 7.91 (m, 5H, 10, 26, 44-H), 7.81 (ddt, J = 10.5, 7.5, 1.5 Hz, 2H, 2-H), 7.74 (d, J = 8.0 Hz, 2H, 4-H), 7.56 (td, J = 7.5, 2.5 Hz, 2H, 3-H), 7.30 – 7.27 (m, 4H, 18, 34-H), 7.07 – 7.02 (m, 8H, 20, 22, 36, 38-H), 4.15 (d, J = 6.5 Hz, 4H, ^{i}Bu), 4.14 (d, J = 6.5 Hz, 6H, ^{i}Bu), 2.14 – 2.10 (m, 5H, ^{i}Bu), 2.07 – 1.81 (m, 8H, ^{n}Bu), 1.71-1.55 (m, 4H, ^{n}Bu), 1.49-1.35 (m, 12H, ^{n}Bu), 1.06 – 1.03 (m, 30H, ^{i}Bu), 0.91 (t, J = 7.0 Hz, 12H, ^{n}Bu);

^{13}C NMR (126 MHz, THF-d_8): δ 166.1 (29, 43-C=O), 166.0 (13-C=O), 160.1 (21-C), 160.0 (37-C), 140.0 (10-C), 139.9 (26, 44-C), 136.3 (d, J = 88.5 Hz, 1-C), 135.9 (4-C), 135.6 (d, J = 9.0 Hz, 6-C), 133.7 (Ar-C), 133.6 (Ar-C), 133.6 (Ar-C), 133.6 (Ar-C), 133.4 (Ar-C), 133.4 (Ar-C), 132.0 (d, J = 8.5 Hz, 2-C), 130.5 (d, J = 11.0 Hz, 3-C), 127.7 (20-C), 127.6 (Ar-C), 126.0 (Ar-C), 126.1 (Ar-C), 125.9 (Ar-C), 125.7 (Ar-C), 125.7 (Ar-C), 125.7 (Ar-C), 125.7 (Ar-C), 121.1 (34-C), 121.0 (18-C), 92.1 (-C≡), 92.0 (-C≡), 91.9 (-C≡), 90.1 (-C≡), 89.1 (-C≡), 89.1 (-C≡), 89.1 (-C≡), 89.0 (-C≡), 73.0 (^{i}Bu), 73.0 (^{i}Bu), 29.3 (d, J = 68.5 Hz, ^{n}Bu), 27.9 (^{i}Bu), 25.3 (d, J = 4.0 Hz, ^{n}Bu) 24.1 (^{n}Bu), 20.3 (^{i}Bu), 20.4 (^{i}Bu), 14.9 (^{n}Bu);

^{31}P NMR (162 MHz, THF-d_8): δ 39.4 (phosphine oxide);

FT-IR (ATR): 3030 (br), 2960, 2199, 1722, 1583, 1236, 768 ν_{max}/cm^{-1};

MS (ES+): m/z (%) = 825.1 (100) [M+2H^{+}], 1648.8 (10) [M+H^{+}].

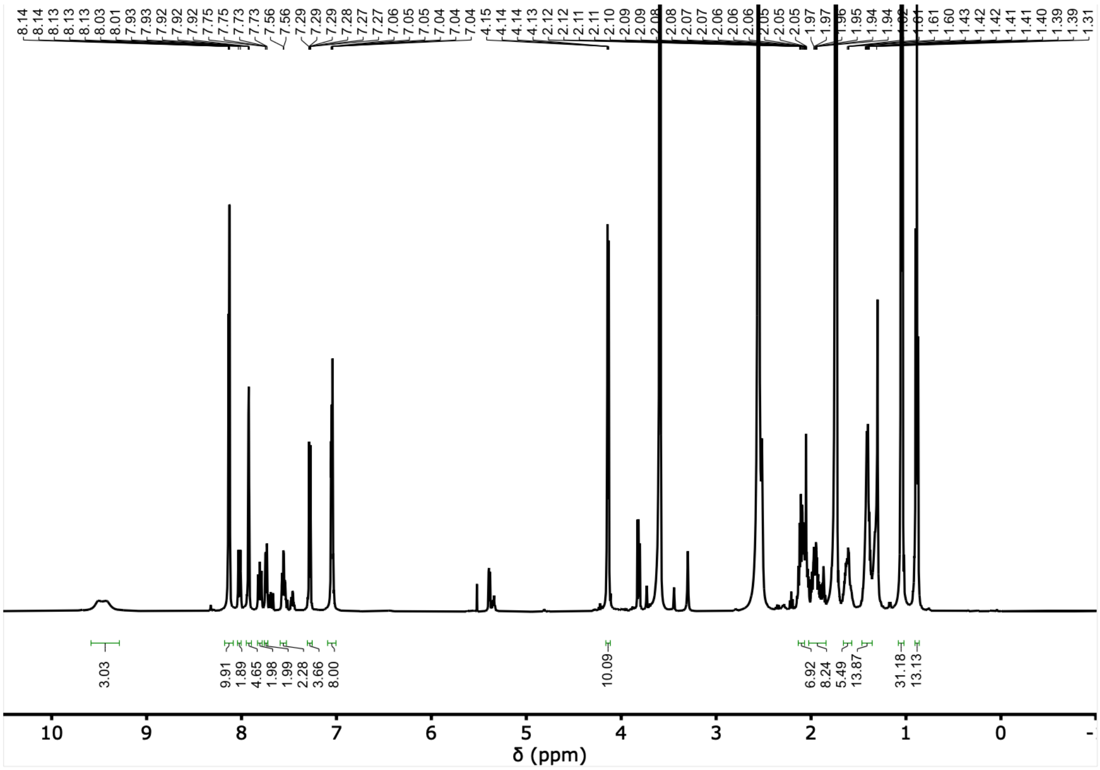

Figure S24 ^{1}H NMR (500 MHz, THF-d_8) of ADDDDA

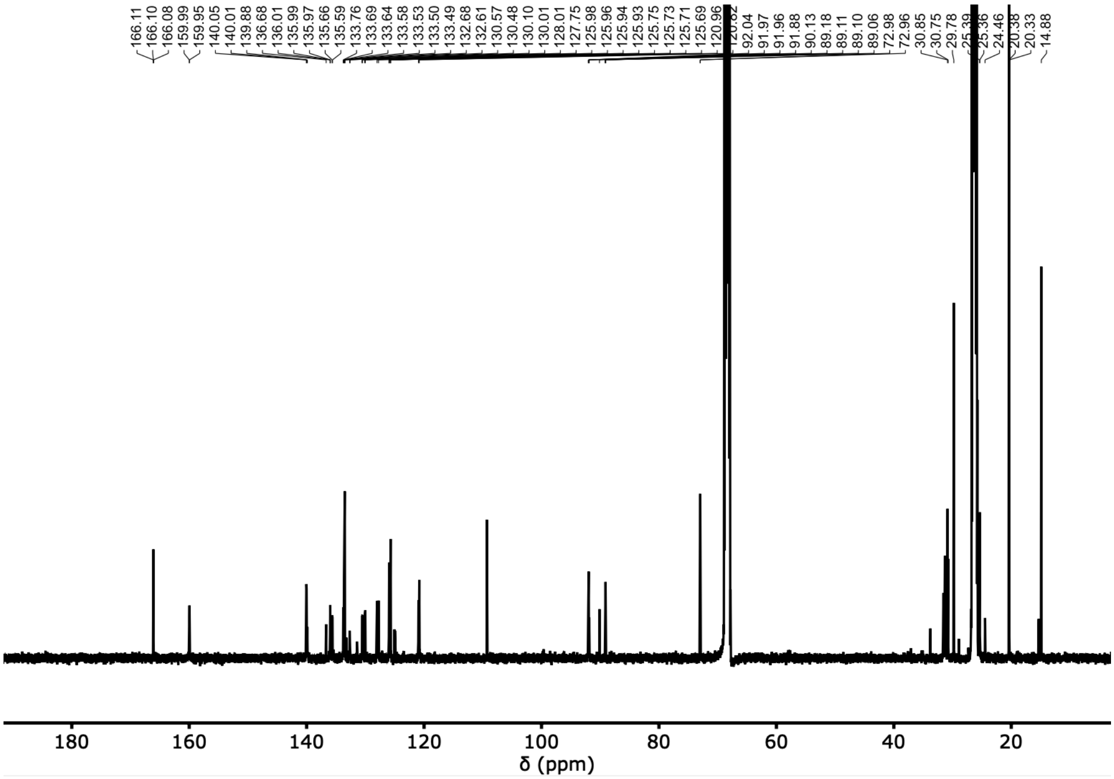

Figure S25 ^{13}C NMR (126 MHz, THF-d_8) of ADDDDA

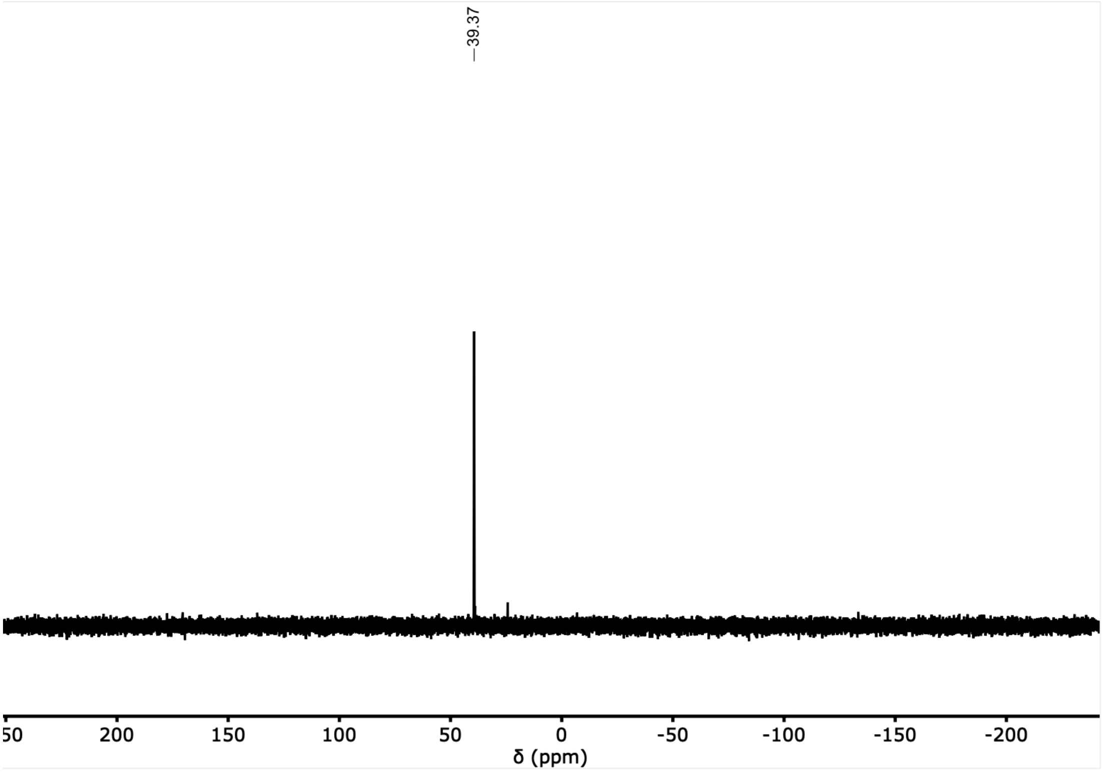

Figure S26 ^{31}P NMR (162 MHz, **THF-*d*$_8$**) of ADDDDA

ADDDDDA 7-mer

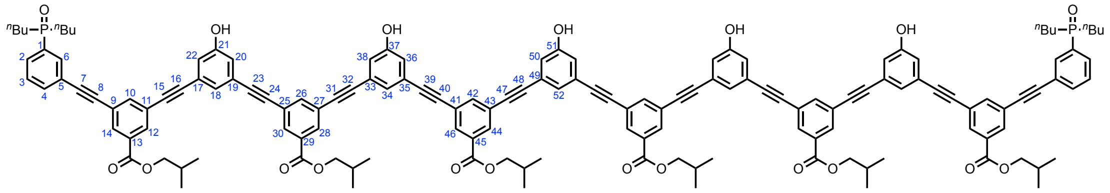

TLC R_f: 0.40 (MeOH:CH_2Cl_2 1:9);

^{1}H NMR (500 MHz, THF-d_8): δ 9.36 (bs, 5H, OH), 8.15 – 8.12 (m, 12H, 12, 14, 28, 30, 44, 46-H), 8.03 (dt, J = 11.0, 1.5 Hz, 2H, 6-H), 7.94 – 7.91 (m, 6H, 10, 26, 42-H),i 7.82 (ddt, J = 10.5, 7.5, 1.5 Hz, 2H, 2-H), 7.75 (d, J = 8.0 Hz, 2H, 4-H), 7.57 (td, J = 7.5, 2.5 Hz, 2H, 3-H), 7.31 – 7.27 (m, 5H, 18, 34, 52-H), 7.08 – 7.02 (m, 10H, 20, 22, 36, 38, 50-H), 4.15 (d, J = 6.5 Hz, 4H, ^{i}Bu), 4.14 (d, J = 6.5 Hz, 8H, ^{i}Bu), 2.14 – 2.10 (m, 10H, ^{i}Bu), 2.07 – 1.81 (m, 4H, ^{n}Bu), 1.71-1.55 (m, 4H, ^{n}Bu), 1.49-1.35 (m, 12H, ^{n}Bu), 1.06 – 1.03 (m, 36H, ^{i}Bu), 0.91 (t, J = 7.0 Hz, 12H, ^{n}Bu);

^{13}C NMR (126 MHz, THF-d_8): δ 166.1 (29, 45-C=O), 166.0 (13-C=O), 160.1 (21-C), 160.0 (37, 51-C), 140.0 (10-C), 139.9 (26, 42-C), 136.3 (d, J = 88.5 Hz, 1-C), 135.9 (4-C), 135.6 (d, J = 9.0 Hz, 6-C), 133.7 (Ar-C), 133.6 (Ar-C), 133.6 (Ar-C), 133.6 (Ar-C), 133.4 (Ar-C), 133.4 (Ar-C), 133.4 (Ar-C), 132.0 (d, J = 8.5 Hz, 2-C), 130.5 (d, J = 11.0 Hz, 3-C), 127.7 (20-C), 127.6 (Ar-C), 127.6 (Ar-C), 126.0 (Ar-C), 126.1 (Ar-C), 125.9 (Ar-C), 125.9 (Ar-C), 125.7 (Ar-C), 125.7 (Ar-C), 125.7 (Ar-C), 121.1 (34, 52-C), 121.0 (18-C), 92.1 (-C≡), 92.0 (-C≡), 91.9 (-C≡), 90.1 (-C≡), 89.1 (-C≡), 89.0 (-C≡), 73.0 (^{i}Bu), 73.0 (^{i}Bu), 29.3 (d, J = 68.5 Hz, ^{n}Bu), 27.9 (^{i}Bu), 25.4 (d, J = 14.5 Hz, ^{n}Bu), 24.5 (d, J = 4.0 Hz, ^{n}Bu) 20.3 (^{i}Bu), 14.9 (^{n}Bu);

^{31}P NMR (162 MHz, $CDCl_3$): δ 39.4 (phosphine oxide);

FT-IR (ATR): 3144 (br), 2963, 2199, 1722, 1583, 1237, 770 ν_{max}/cm^{-1};

MS (ES+): m/z (%) = 983.4 (100) [M+2H$^+$], 1965.2 (5) [M+H$^+$]

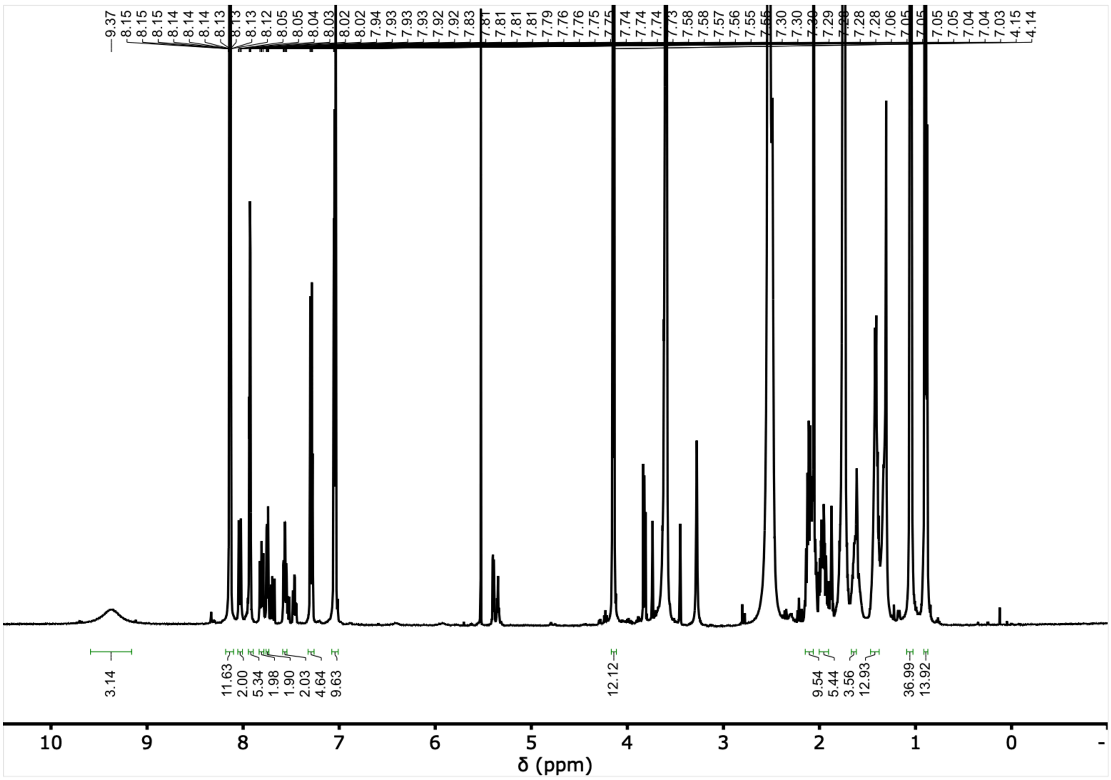

Figure S27 ^{1}H NMR (500 MHz, THF-d_8) of ADDDDDA

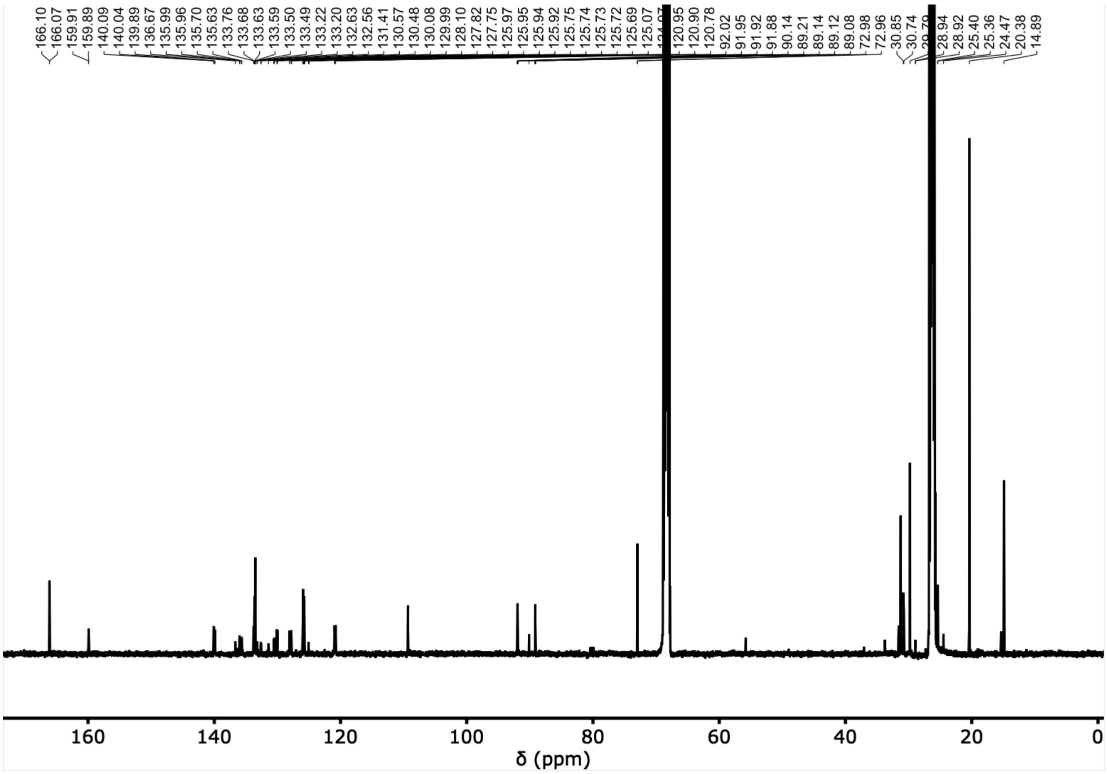

Figure S28 ^{13}C NMR (126 MHz, THF-d_8) of ADDDDDA

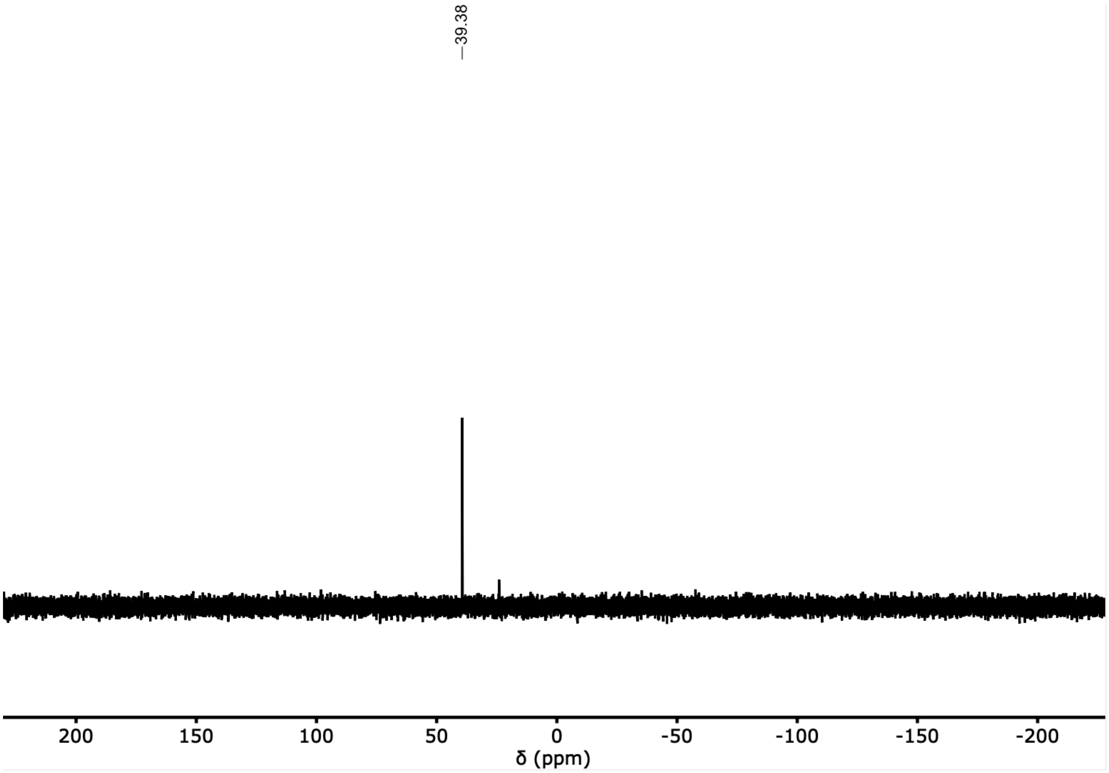

Figure S29 ^{31}P NMR (162 MHz, THF-d_8) of ADDDDDA

Binding studies

NMR dilution experiments

Self association constants were measured by ^{31}P NMR dilution experiments in a Bruker 500 MHz AVIII HD Smart Probe spectrometer. The oligomer was dissolved in $CDCl_3$ at a known concentration. A known volume of $CDCl_3$ was added to an NMR tube, aliquots of the oligomer were added, and the spectrum was recorded after each addition. The chemical shifts of the each signals in the spectrum was monitored as a function of oligomer concentration and analysed using purpose written software in Microsoft Excel. Errors were calculated as two times the standard deviation from the average value (95% confidence limit). The data for self-association of DAD, DAAD, DAAAD, ADA, ADDA and ADDDA are reported below.

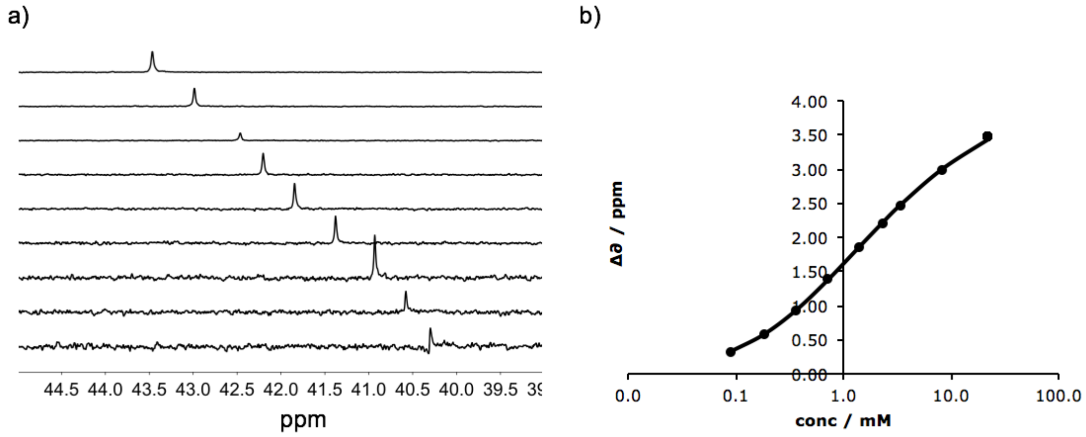

Figure S30: a) ^{31}P NMR (202 MHz, $CDCl_3$-d_3) data for dilution of DAD at 298 K (b) Plot of the change in chemical shift as a function of concentration (the line represents the best fit to a dimerisation isotherm).

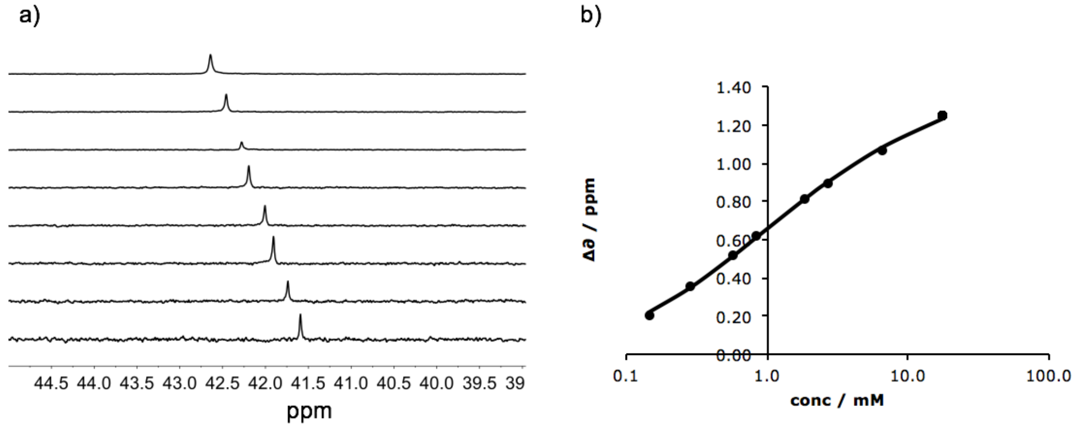

Figure S31: a) ^{31}P NMR (202 MHz, $CDCl_3$-d_3) data for dilution of DAAD at 298 K - (b) Plot of the change in chemical shift as a function of concentration (the line represents the best fit to an isodesmic polymerisation isotherm).

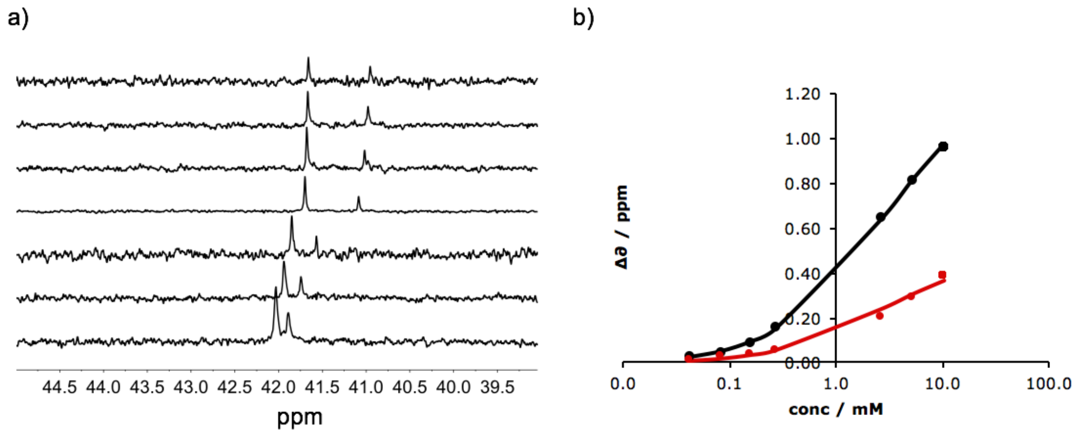

Figure S32 : a) ^{31}P NMR (202 MHz, $CDCl_3$-d_3) data for dilution of DAAAD at 298 K - (b) Plot of the change in chemical shift as a function of concentration (the line represents the best fit to an isodesmic polymerisation isotherm).

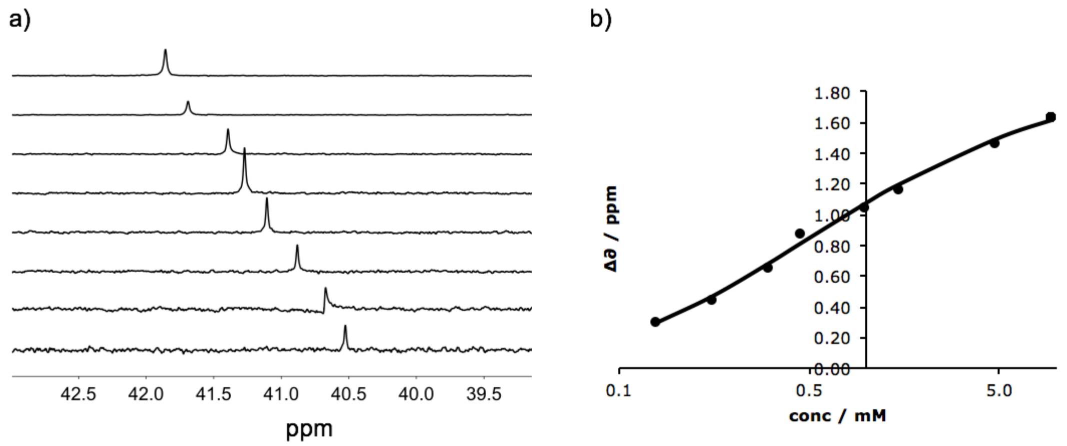

Figure S33 : a) ^{31}P NMR (202 MHz, $CDCl_3$-d_3) data for dilution of ADA at 298 K - (b) Plot of the change in chemical shift as a function of concentration (the line represents the best fit to a dimerisation isotherm).

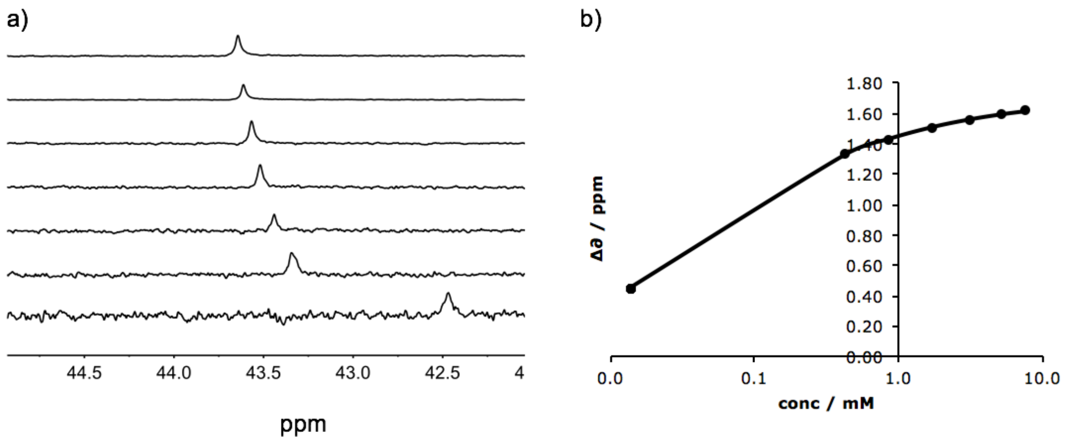

Figure S34: a) ^{31}P NMR (202 MHz, $CDCl_3$-d_3) data for dilution of ADDA at 298 K - (b) Plot of the change in chemical shift as a function of concentration (the line represents the best fit to a dimerisation isotherm).

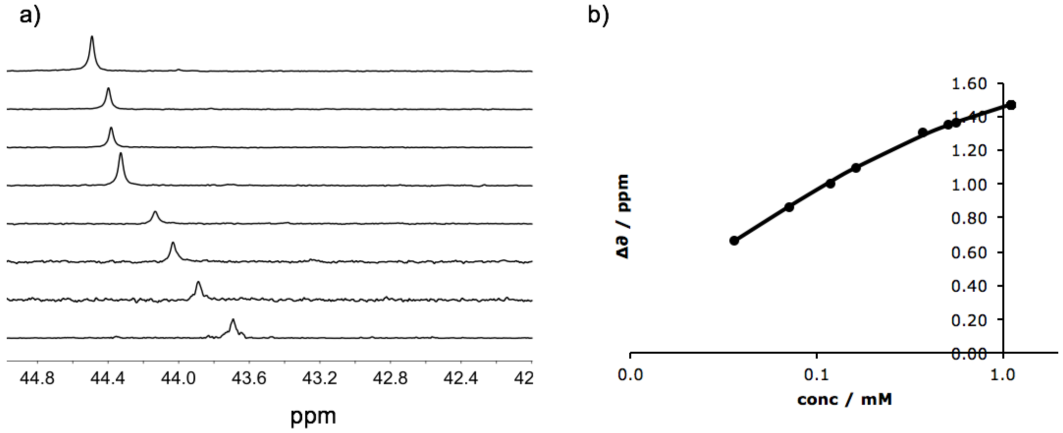

Figure S35: a) ^{31}P NMR (202 MHz, $CDCl_3$-d_3) data for dilution of ADDDA at 298 K - (b) Plot of the change in chemical shift as a function of concentration (the line represents the best fit to a dimerisation isotherm).

NMR titration experiments

A 5 mM solution of **D** was prepared in $CDCl_3$. A known volume of the solution was added to an NMR tube and the ^{31}P spectrum was recorded. Known volumes of DMSO-d_6 in $CDCl_3$ (or neat DMSO-d_6) were added, and the spectrum was recorded after each addition. The ^{31}P chemical shifts were monitored as a function of DMSO-d_6 concentration, and the data were analysed using purpose written software in Microsoft Excel.

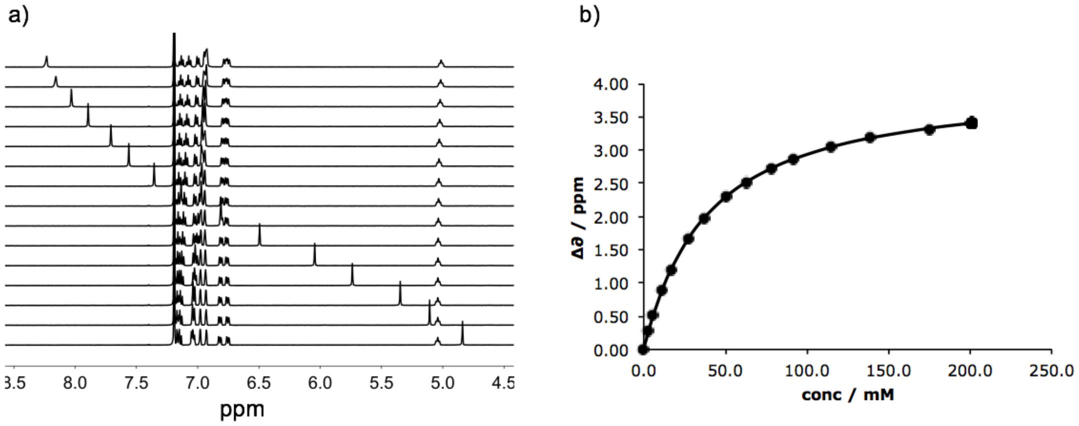

Figure S36 a) ^{31}P NMR (202 MHz, $CDCl_3$-d_3) data for titration of DMSO-d_6 into **D** (5 mM) at 298 K in $CDCl_3$- (b) Plot of the change in chemical shift as a function of concentration (the line represents the best fit to 1:1 binding isotherm).

NMR duplex denaturation experiments

Association constants for duplex formation were measured by ^{31}P NMR denaturation experiments in a Bruker 500 MHz AVIII HD Smart Probe spectrometer. An equimolar solution of complementary oligomers (AAA•DDD, ADA•DAD, ADDA•DAAD and ADDDA•DAAAD) was produced at known concentrations (0.3 mM-1.0 mM) in $CDCl_3$. A known volume of the solution was added to an NMR tube and the ^{31}P spectrum was recorded. Known volumes of DMSO-d_6 in $CDCl_3$ (or neat DMSO-d_6) were added, and the spectrum was recorded after each addition. The ^{31}P chemical shifts were monitored as a function of DMSO-d_6 concentration. ^{31}P NMR shifts were monitored for a 4.9 mM solution of A in $CDCl_3$ with the same concentrations of DMSO-d_6, and these values were subtracted from the values recorded in the denaturation experiment to account for solvent effects. The corrected data were analysed using purpose written software in Microsoft Excel.

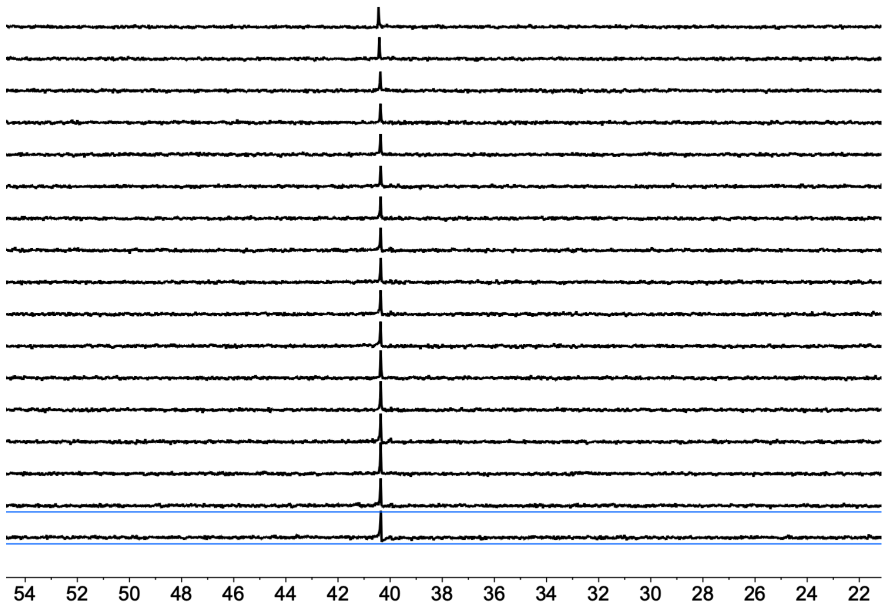

Figure S37 Free ^{31}P NMR (202 MHz) shifts of a 5.6 mM solution of A 4.9 mM in $CDCl_3$ in the presence of different concentrations of DMSO-d_6 (0-200 mM).

The denaturation data did not fit to a simple two-state isotherm for any of the duplexes. Therefore all of the denaturation data were analysed taking into account partially denatured species. In order to fit the denaturation data using the minimum number of variables, the association constants for certain species were fixed at values that were determined in independent experiments (e.g. K_d is the **D**•DMSO association constant, and K_N is the association constant for duplex formation between complementary oligomers of length N in $CDCl_3$-*d3*). For some of the complexes, isomeric arrangements are possible, and a statistical factor is included below to account for degenerate arrangements of free and bound base-pairing interactions. In addition, the chemical shifts of the partially denatured species were fixed using population-weighted average of the chemical shifts of the fully bound duplex and fully denatured species based on the number of phenol-phosphine oxide H-bonding interactions present. Thus although the binding isotherms are complicated and involve a large number of different species, the fitting process is robust, because the only variables that are fit in each experiment are the association constant for duplex formation, the chemical shift of the fully assembled duplex, and the chemical shifts of the two fully denatured oligomers. In all cases, good fits were obtained, and the optimised chemical shifts of the two fully denatured oligomers were very close to values observed for AAA at high concentrations of DMSO.

AAA•DDD

The association constant for duplex formation and the free and the bound ^{31}P NMR chemical shifts were optimised. The association constants were fixed for the following species:

K (DDD•DMSO) = 3 K_d

K (DDD•2DMSO) = 3 K_d^2

K (DDD•3DMSO) = K_d^3

K (AAA•DDD•DMSO) = 3 K_2 K_d

K (AAA•DDD•2DMSO) = 3 K K_d^2

The chemical shifts were fixed for the following species:

$$\delta_{\mathrm{AAA\bullet DDD\bullet DMSO}} = \frac{(2\delta_{\mathrm{bound}} + \delta_{\mathrm{free}})}{3}$$

$$\delta_{\mathrm{AAA\bullet DDD\bullet (DMSO)_2}} = \frac{(\delta_{\mathrm{bound}} + 2\delta_{\mathrm{free}})}{3}$$

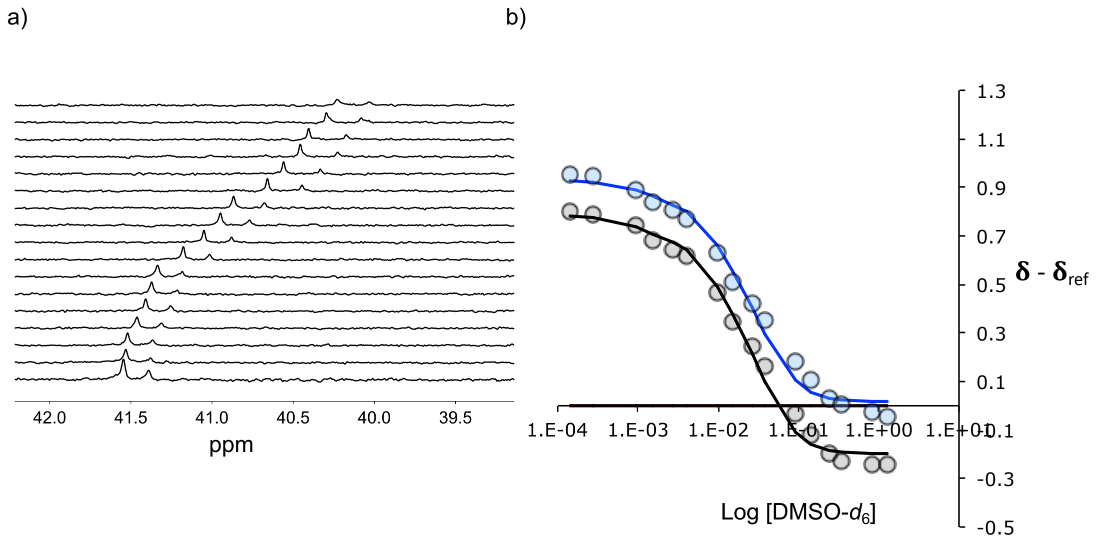

Figure S38 Free^{31}P NMR (202 MHz, $CDCl_3$-d_3) data for DMSO-d_6 denaturation of AAA•DDD (1.2 mM, 0.64 mM) at 298 K in $CDCl_3$ (b) Plot of the change in ^{31}P chemical shift as a function of DMSO-d_6 concentration The dots represent the experimental values, and the lines calculated denaturation isotherms considering all possible species.

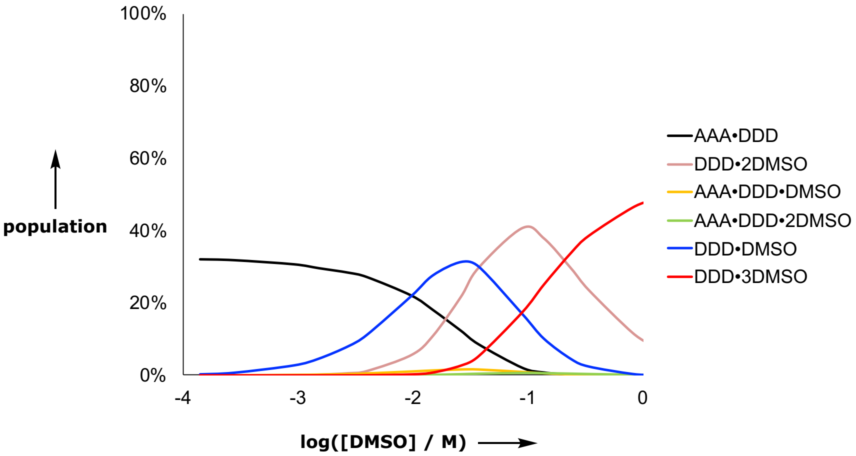

Figure S39: Calculated speciation for DMSO denaturation of AAA• DDD (1.2 mM, 0.64 mM) at 298 K in $CDCl_3$.

ADA•DAD

The association constant for duplex formation and the free and the bound ^{31}P NMR chemical shifts were optimised. The association constants were fixed for the following species:

K (ADA•DMSO) = K_d

K (DAD•DMSO) = 2 K_d

K (DAD•2DMSO) = K_d^2

K (ADA•DAD•DMSO) = 3 K_2 K_d

K (ADA•DAD•2DMSO) = 3 K_1 K_d^2

DMSO can also bind to the unbound phenols on the chain ends of the DAD•DAD complex:

K (DAD•DAD•DMSO) = 2 K(DAD•DAD) K_d

K (DAD•DAD•2DMSO) = K(DAD•DAD) K_d^2

And the following values are known from the dilution experiments:

K (DAD•DAD) = 490 M^{-1}

K (ADA•ADA) = 1,360 M^{-1}

The chemical shifts were fixed for the following species:

$$\delta_{\text{ADA•DAD•DMSO}} = \frac{(2\delta_{\text{bound}} + \delta_{\text{free}})}{3}$$

$$\delta_{\text{ADA•DAD•(DMSO)}_2} = \frac{(\delta_{\text{bound}} + 2\delta_{\text{free}})}{3}$$

$$\delta_{\text{ADA•DMSO}} = \delta_{\text{ADA}}$$

$$\delta_{\text{DAD•DMSO}} = \delta_{\text{DAD•2DMSO}} = \delta_{\text{DAD}}$$

And the following values are known from the dilution experiments:

$$\delta_{\text{ADA•ADA}} = 2.0 \text{ ppm}$$

$$\delta_{\text{DAD•DAD}} = \delta_{\text{DAD•DAD•DMSO}} = \delta_{\text{DAD•DAD•2DMSO}} = 4.3 \text{ ppm}$$

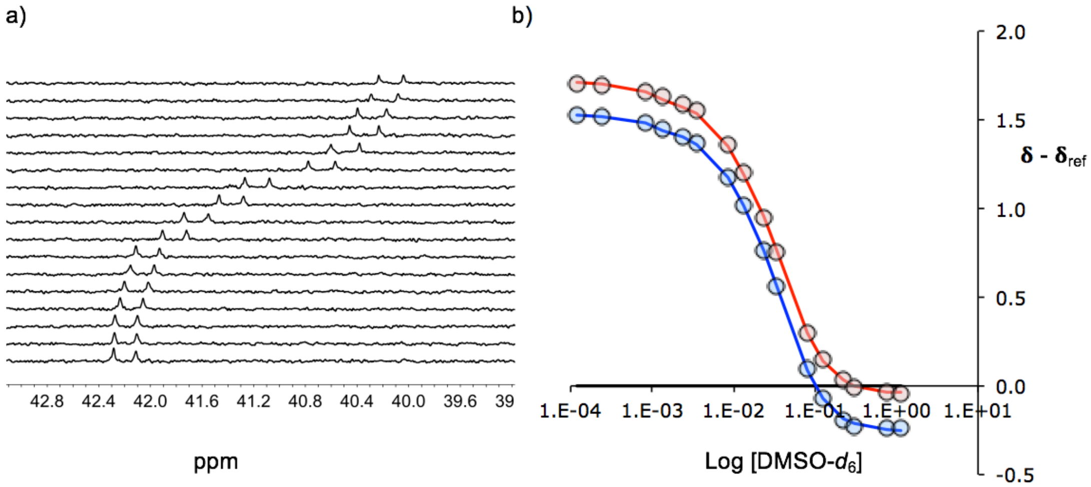

Figure S40 ^{31}P NMR (202 MHz, $CDCl_3$-d_3) data for DMSO-d_6 denaturation of ADA•DAD (0.4 mM, 0.4 mM) at 298 K (b) Plot of the change in ^{31}P chemical shift as a function of DMSO-d_6 concentration The dots represent the experimental values, and the lines calculated denaturation isotherms considering all possible species.

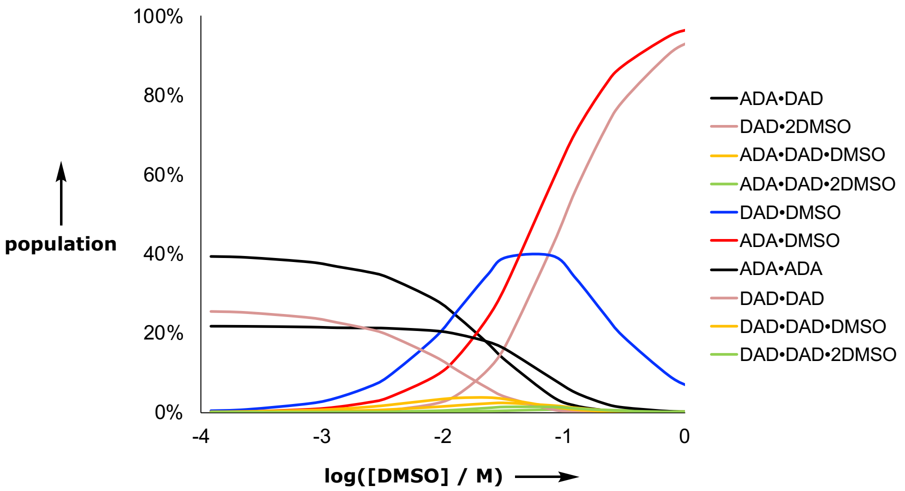

Figure S41: Speciation from DMSO denaturation experiments on complementary oligomer duplex ADA•DAD (0.4 mM, 0.4 mM).

ADDA•DAAD

The association constant for duplex formation and the free and the bound ^{31}P NMR chemical shifts were optimised. The association constants were fixed for the following species:

K (ADDA•DMSO) = 2 K_d

K (ADDA•2DMSO) = K_d^2

K (DAAD•DMSO) = 2 K_d

K (DAAD•2DMSO) = K_d^2

K (ADDA•DAAD•DMSO) = 4 K_3 K_d

K (ADDA•DAAD•2DMSO) = 6 K_2 K_d^2

And the following values are known from the dilution experiments:

K(ADDA•ADDA) = 22,200 M^{-1}

K(DAAD•DAAD) = 575 M^{-1}

The following species can be shown not to be populated to any significant extent:

K (ADDA•DAAD•3DMSO) = 4 K_1 K_d^3

K (DAAD•DAAD•DMSO) = 2 K(DAAD•DAAD) K_d

K (DAAD•DAAD•2DMSO) = K(DAAD•DAAD) K_d^2

The chemical shifts were fixed for the following species:

$$\delta_{\text{ADDA•DAAD•DMSO}} = \frac{(3\delta_{\text{bound}} + \delta_{\text{free}})}{4}$$

$$\delta_{\mathrm{ADDA\bullet DAAD\bullet (DMSO)_2}} = \frac{(\delta_{\mathrm{bound}} + \delta_{\mathrm{free}})}{2}$$

$$\delta_{\mathrm{DAAD\bullet DMSO}} = \delta_{\mathrm{DAAD\bullet 2DMSO}} = \delta_{\mathrm{DAAD}}$$

$$\delta_{\mathrm{ADDA\bullet DMSO}} = \delta_{\mathrm{ADDA\bullet 2DMSO}} = \delta_{\mathrm{ADDA}}$$

And the following values are known from the dilution experiments:

$$\delta_{\mathrm{ADDA\bullet ADDA}} = 1.7 \text{ ppm}$$

$$\delta_{\mathrm{DAAD\bullet DAAD}} = \delta_{\mathrm{DAD\bullet DAD\bullet 2DMSO}} = 1.7\text{ppm}$$

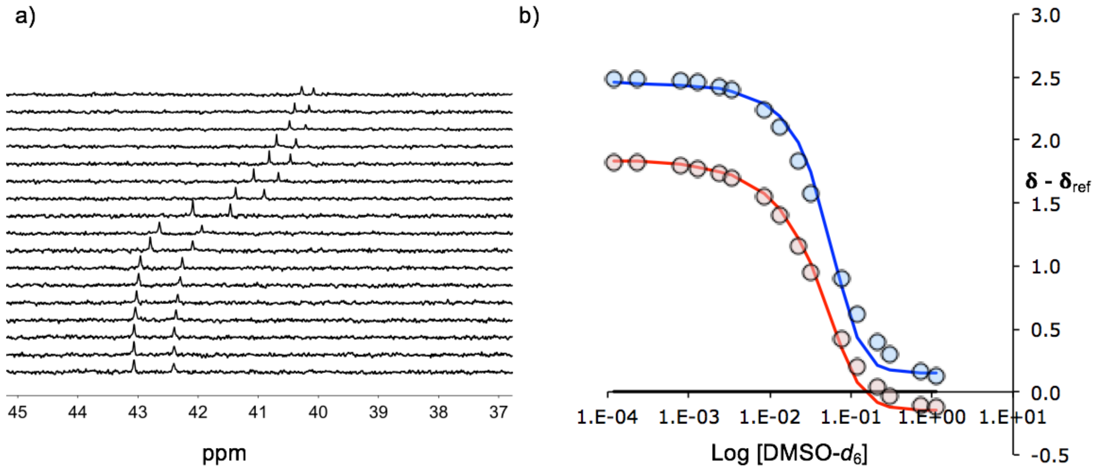

Figure S42 ^{31}P NMR (202 MHz, $CDCl_3$-d_3) data for DMSO-d_6 denaturation of ADDA•DAAD (0.5 mM, 0.5 mM) at 298 K in $CDCl_3$ (b) Plot of the change in ^{31}P chemical shift as a function of DMSO-d_6 concentration The dots represent the experimental values, and the lines calculated denaturation isotherms considering all possible species.

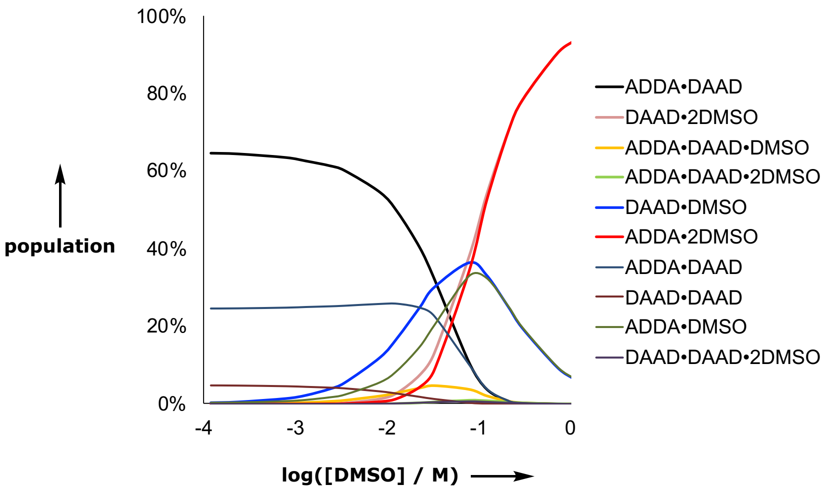

Figure S43: Speciation from DMSO denaturation experiments on complementary oligomer duplex ADDA•DAAD (0.5 mM, 0.5 mM).

ADDDA•DAAAD

The association constant for duplex formation and the free and bound ^{31}P NMR chemical shifts were optimised. The association constants were fixed for the following species:

K (ADDDA•DMSO) = 3 K_d

K (ADDDA•2DMSO) = 3 K_d^2

K (ADDDA•3DMSO) = K_d^3

K (DAAAD•DMSO) = 2 K_d

K (DAAAD•2DMSO) = K_d^2

K (ADDDA•DAAAD•DMSO) = 5 K_4 K_d

K (ADDDA•DAAAD•2DMSO) = 10 K_3 K_d^2

And the following values are known from the dilution experiments:

K (ADDDA•ADDDA) = 14,350 M^{-1}

K (DAAAD•DAAAD) = 210 M^{-1}

The following species can be shown not to be populated to any significant extent:

K (ADDDA•DAAAD•3DMSO) = 10 K_2 K_d^3

K (ADDDA•DAAAD•4DMSO) = 5 K_1 K_d^4

K (DAAAD•DAAAD•DMSO) = 2 K(DAAAD•DAAAD) K$_d$

K (DAAAD•DAAAD•2DMSO) = K(DAAAD•DAAAD) K$_d^2$

The chemical shifts were fixed for the following species:

$$\delta_{\text{ADDDA•DAAAD•DMSO}} = \frac{(4\delta_{\text{bound}} + \delta_{\text{free}})}{5}$$

$$\delta_{\text{ADDDA•DAAAD•2DMSO}} = \frac{(3\delta_{\text{bound}} + 2\delta_{\text{free}})}{5}$$

$$\delta_{\text{DAAAD•DMSO}} = \delta_{\text{DAAAD•2DMSO}} = \delta_{\text{DAAAD}}$$

$$\delta_{\text{ADDDA•DMSO}} = \delta_{\text{ADDDA•2DMSO}} = \delta_{\text{ADDDA•3DMSO}} = \delta_{\text{ADDDA}}$$

And the following values are known from the dilution experiments:

$$\delta_{\text{ADDDA•ADDDA}} = 1.8 \text{ ppm}$$

$$\delta_{\text{DAAAD•DAAAD}} = 1.5 \text{ ppm}$$

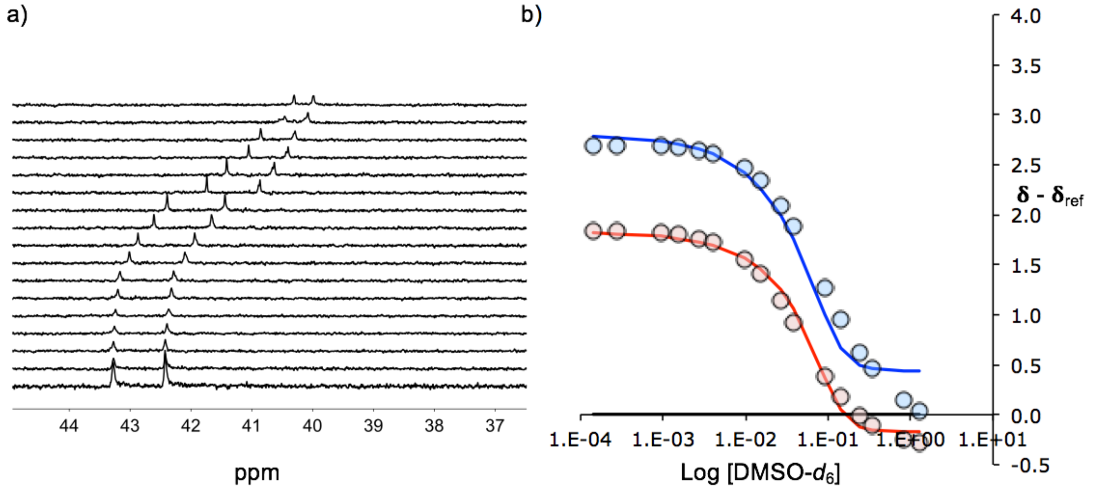

Figure S44 ^{31}P NMR (202 MHz, $CDCl_3$-d_3) data for DMSO-d_6 denaturation of ADDDA•DAAAD (0.3 mM, 0.3 mM) at 298 K in $CDCl_3$ (b) Plot of the change in ^{31}P chemical shift as a function of DMSO-d_6 concentration The dots represent the experimental values, and the lines calculated denaturation isotherms considering all possible species.

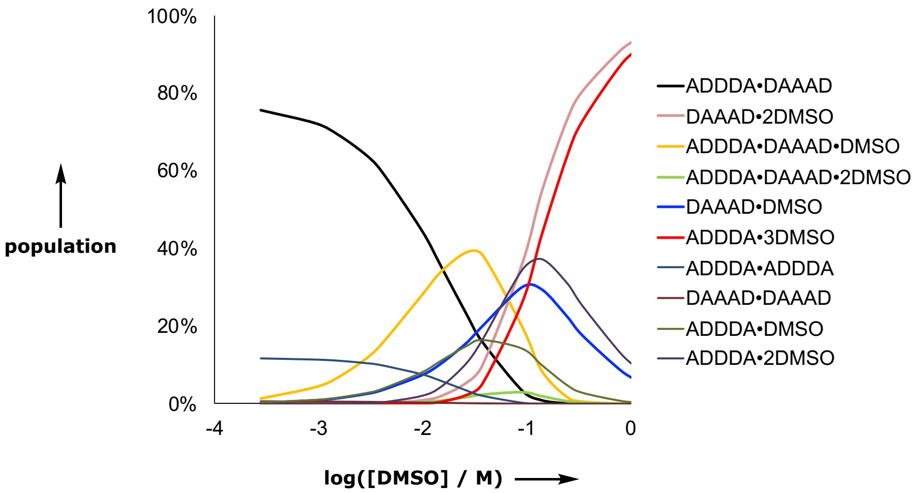

Figure S45: Speciation from DMSO denaturation experiments on complementary oligomer duplex ADDDA•DAAAD (0.3 mM, 0.3 mM).

X-ray crystal structure of DAD

DAD (5 mg) was dissolved in 10% CH_2Cl_2 in MeCN (0.5 mL), and the mixture was filtered to a vial and sealed with a plastic cap with a small hole, resulting in crystallization after 10 days at room temperature. Crystals suitable for X-ray analysis were selected using an optical microscope and examined at 180 K on a Bruker D8-QUEST instrument, fitted with a PHOTON-100 detector and Incoatec IµS Cu microsource (λ_{ave} = 1.5418 Å).

The structure analysis proved to be difficult, and the result is the best obtained from several crystals. The diffraction pattern looks normal (albeit with relatively rapid drop off of intensity higher angle), and the established monoclinic *C* unit cell fits 100% of the measured reflections. There is no sign of any additional crystal component in the diffraction frames, nor of any streaking or split reflections. However, several observations indicate a potential for twinning:

(1) The lattice approximates orthorhombic *F* (11.6, 24.5, 71.9, 90, 90, 90). The additional 2-fold axes of point group *mmm* yield two (equivalent) potential twin laws: (–1 0 0 / 0 –1 0 / 1 0 1) or (1 0 0 / 0 1 0 / –1 0 –1). However, neither twin law improves the agreement to the data.

(2) Some reflections are observed that should be absent for space group *C*2/*c*.

(3) For the poorest fitting data, *F*(obs) is consistently larger than *F*(calc).

Analysis with TWINROTMAT and ROTAX failed to identify any feasible twin laws. Attempts to refine the structure in lower symmetry space groups (e.g. $P2_1/n$, *P*–1, with consideration of potential twinning) also did not yield any improvement. Hence, the presented *C*2/*c* structure is the best that could be obtained.

The analysis shows the molecular structure clearly. The molecules lie in (centred) layers parallel to the *ab* planes with disorder of the butyl chains on P occurring at the interlayer region. Selecting one of the butyl chain components in any given site leaves voids where the other chain component lies. These voids may be occupied by solvent molecules (most likely MeCN) in the crystal.

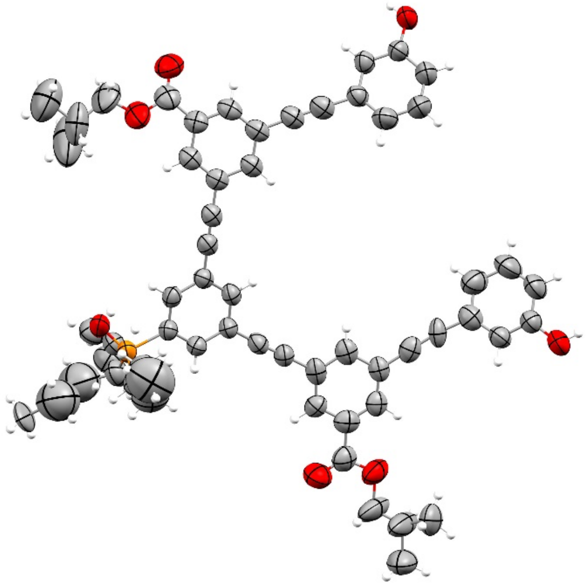

Figure S46: X-ray structure of **DAD** in ORTEP view (ellipsoids are drawn at 50% probability level).

Summary of crystallographic data

CCDC number	2079477
Cambridge data number	CH_B2_0023
Chemical formula	$C_{56}H_{55}O_7P$
Formula weight	870.97
Temperature / K	180(2)
Crystal system	monoclinic
Space group	*C*2/*c*
a / Å	24.5550(7)
b / Å	11.5883(3)
c / Å	37.9435(10)
α / °	90
β / °	108.3188(14)
γ / °	90
Unit-cell volume / Å^3	10249.7(5)
Z	8
Calc. density / g cm^{-3}	1.129
F(000)	3696
Radiation type	CuKα
Absorption coefficient / mm^{-1}	0.865
Crystal size / mm^3	0.22 x 0.12 x 0.08
2θ range / °	7.58-133.63
Completeness to max 2θ	0.997
No. of reflections measured	58401
No. of independent reflections	9082
R(int)	0.0827
No. parameters / restraints	605 / 101
Final R1 values (I > 2σ(I))	0.1418
Final wR(F^2) values (all data)	0.1842
Goodness-of-fit on F^2	1.039
Largest difference peak & hole / e Å^{-3}	1.158, –0.495

Molecular Mechanics Calculations

General details

Molecular mechanics calculations were performed using MacroModel version 9.8 (MacroModel, version 9.8, Schrödinger, LLC, New York, NY, 2014). Calculations were performed on simplified oligomers in which the solubilising groups were changed to methyl groups in order to reduce the computational cost. All structures were minimized first and the minimized structures were then used as the starting molecular structures for all MacroModel conformational searches. The force field used was MMFFs as implemented in this software ($CHCl_3$ solvation). The charges were defined by the force field library and no cut off was used for non-covalent interactions. A Polak-Ribiere Conjugate Gradient (PRCG) was used and each minimisation was subjected to 10000 iterations. The minima converged on a gradient with a threshold of 0.1. The sampling method was selected to be large scale low mode sampling. Conformational searches were performed from previously minimized structures using 10000 steps. Calculations were repeated three times from different starting conformations. The conformations shown are the lowest energy structures and the images were created using PyMol (The PyMOL Molecular Graphics System, Version 1.6 Schrödinger, LLC).

Single strands

For the **DAD** and **ADA** 3-mers, the 50 lowest energy structures were all within 0.5 kJ mol^{-1} with none forming intramolecular H-bonds. For the **DAAD** 4-mer, the 50 lowest energy structures were all within 3.0 kJ mol^{-1} with the same backbone conformation forming two intramolecular H-bonds in each structure. For the **ADDA** 4-mer, the 50 lowest energy structures were all within 32 kJ mol^{-1} with the same backbone conformation forming two intramolecular H-bonds in each structure within 26 kJ mol^{-1} from the global minimum. For the **DAAAD** 5-mer, the 50 lowest energy structures were all within 6.0 kJ mol^{-1} with the same backbone conformation forming two intramolecular H-bonds in each structure. For the **ADDDA** 5-mer, the 50 lowest energy structures were all within 24 kJ mol^{-1} with the same backbone conformation forming two intramolecular H-bonds in each structure within 18 kJ mol^{-1} from the global minimum.

Duplexes

For each duplex, multiple conformational searches were performed using distance constraints to fix H-bonding interactions between all possible pairwise combinations of donor and acceptor. For each H-bond, the distance between the phenol hydrogen and phosphine oxide oxygen was constrained to 2 ± 1 Å. In addition for each H-bond arrangement, three different starting conformations were used. For each duplex, the results from all of the conformational searches were compared, and the lowest energy structure was selected. For the **DAAD•DAAD** and **ADDA•ADDA** duplexes, the lowest energy conformation corresponds to a structure with 4 intermolecular H-bonds in a criss-cross conformation. For the **DAAAD•DAAAD** and **ADDDA•ADDDA** duplexes, the lowest energy conformation corresponds to a structure with 4 intermolecular H-bonds between terminal groups in a criss-cross conformation, and the recognition unit in the middle remaining unpaired. For the **ADA•DAD**, **ADDA•DAAD** and **ADDDA•DAAAD** duplexes, the lowest energy conformation corresponds with a fully H-bonded duplex with all the phenols and phosphine oxides paired and the oligomers adopting a zig-zag conformation.
